# Ubiquitin Ser65 phosphorylation affects ubiquitin structure, chain assembly and hydrolysis

**DOI:** 10.15252/embj.201489847

**Published:** 2014-12-20

**Authors:** Tobias Wauer, Kirby N Swatek, Jane L Wagstaff, Christina Gladkova, Jonathan N Pruneda, Martin A Michel, Malte Gersch, Christopher M Johnson, Stefan MV Freund, David Komander

**Affiliations:** Medical Research Council Laboratory of Molecular BiologyCambridge, UK

**Keywords:** deubiquitinase, Parkin, phosphorylation, PINK1, ubiquitin

## Abstract

The protein kinase PINK1 was recently shown to phosphorylate ubiquitin (Ub) on Ser65, and phosphoUb activates the E3 ligase Parkin allosterically. Here, we show that PINK1 can phosphorylate every Ub in Ub chains. Moreover, Ser65 phosphorylation alters Ub structure, generating two conformations in solution. A crystal structure of the major conformation resembles Ub but has altered surface properties. NMR reveals a second phosphoUb conformation in which β5-strand slippage retracts the C-terminal tail by two residues into the Ub core. We further show that phosphoUb has no effect on E1-mediated E2 charging but can affect discharging of E2 enzymes to form polyUb chains. Notably, UBE2R1- (CDC34), UBE2N/UBE2V1- (UBC13/UEV1A), TRAF6- and HOIP-mediated chain assembly is inhibited by phosphoUb. While Lys63-linked poly-phosphoUb is recognized by the TAB2 NZF Ub binding domain (UBD), 10 out of 12 deubiquitinases (DUBs), including USP8, USP15 and USP30, are impaired in hydrolyzing phosphoUb chains. Hence, Ub phosphorylation has repercussions for ubiquitination and deubiquitination cascades beyond Parkin activation and may provide an independent layer of regulation in the Ub system.

## Introduction

The covalent modification of Lys residues with the 76 amino acid protein ubiquitin (Ub) constitutes one of the most important cellular signals, most commonly targeting the substrate protein for proteasomal degradation (Hershko & Ciechanover, 1998). The last decade has revealed that ubiquitination is much more versatile and affects virtually all cellular processes, including transcription, translation, protein kinase, cytokine and DNA damage signaling, intracellular trafficking and most forms of protein degradation, such as ERAD, autophagy and mitophagy (Chen & Sun, 2009; Komander & Rape, 2012; Shaid *et al*, 2013; Randow & Youle, 2014). With such wide-ranging roles, it is not surprising that imbalances in the Ub system lead to disease, and many proteins involved in assembly, binding or disassembly of Ub conjugates are mutated in human disorders. A prime example is Parkinson's disease, a neurodegenerative disorder characterized by loss of dopaminergic neurons, which affects 1–2% of the human population especially at an older age. Mutations in the Ub E3 ligase Parkin predispose individuals to autosomal recessive juvenile Parkinsonism (AR-JP), a form of the disease where neurological symptoms show at an early age. Heterozygous mutations of Parkin have also been implicated in the more common, late-onset form of the disease (Sun *et al*, 2006; Wang *et al*, 2008; Corti *et al*, 2011). Parkin belongs to the family of RBR E3 ligases (Wenzel & Klevit, 2012), and recent structural data revealed that Parkin is autoinhibited and requires activation (Riley *et al*, 2013; Trempe *et al*, 2013; Wauer & Komander, 2013). Parkin is activated by the protein kinase PINK1, which is stabilized on depolarized mitochondria upon mitochondrial damage (Youle & Narendra, 2011). Once activated, Parkin ubiquitinates numerous mitochondrial and cytosolic proteins (Chan *et al*, 2011; Sarraf *et al*, 2013) including mitofusins and Miro, eventually triggering mitophagy (Youle & Narendra, 2011).

Many substrates of PINK1 and mechanisms for PINK1-mediated Parkin activation have been postulated (Wang *et al*, 2011; Okatsu *et al*, 2012; Chen & Dorn, 2013). Most recently, based on mass spectrometry and following from earlier findings that PINK1 phosphorylates the Parkin Ub-like (Ubl) domain (Kondapalli *et al*, 2012), three groups showed that Ub itself is a substrate for PINK1, which phosphorylates Ub Ser65 exclusively, *in vitro* and in cells (Kane *et al*, 2014; Kazlauskaite *et al*, 2014; Koyano *et al*, 2014). Moreover, purified phosphoUb directly activated Parkin in the absence of PINK1, suggesting a new model of Parkin activation (Sauvé & Gehring, 2014; Zheng & Hunter, 2014) which is still being refined (Ordureau *et al*, 2014).

Aside from this gain-of-function role of phosphoUb in Parkin activation, it is unclear whether phosphorylation has structural consequences for Ub and whether phosphorylation affects other Ub-mediated processes. These fundamental questions have not been addressed to date.

We here show that PINK1 is a Ub and polyUb kinase and reveal wide-ranging consequences of Ub Ser65 phosphorylation. These include significant structural changes within phosphoUb wherein the protein interconverts between two conformations in solution, one of which has a retracted C-terminal tail; this Ub^retraCT^ conformation has not been observed to date. While phosphoUb is still activated by E1 and charged onto E2 enzymes, discharging and polyUb chain assembly is impaired in several E2 enzymes and E2/E3 complexes. Moreover, 10 out of 12 tested deubiquitinases (DUBs) hydrolyze phosphoUb chains with significantly lower activity.

In summary, we show that PINK1-mediated phosphorylation of Ub generates a functionally altered Ub that would be incompetent in various Ub-regulated processes, consistent with a model that Ser65-phosphorylated Ub is an independent signal with altered functions in cells.

## Results

### PINK1 is a Ub kinase

**Figure 1:**
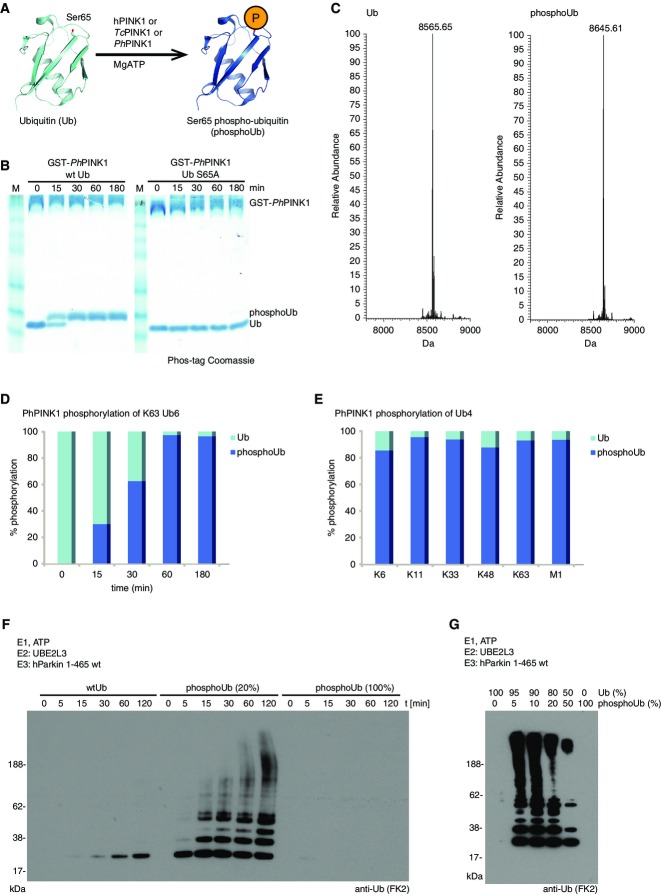
Generation of Ser65-phosphoUb
Schematic representation of PINK1 variants phosphorylating Ub at Ser65. hPINK1, human PINK1; *Tc*PINK1, *Tribolium castaneum*PINK1; *Ph*PINK1, *Pediculus humanus corporis*PINK1.Phos-tag gel following complete conversion of Ub to phosphoUb, using GST-*Ph*PINK1. A Ser65 mutant Ub is not modified.ESI-MS analysis of pure Ub (left, mass 8,565.65 Da, reference 8,565.80 Da) and purified phosphoUb (right, mass 8,645.61 Da, reference 8,645.80 Da). The difference of 80 Da corresponds to one phosphate group.AQUA analysis of *Ph*PINK1-phosphorylated Lys63-linked hexaUb. The Lys63 peptide (K63) is modified by Ser65 phosphorylation during the time course, as observed by rise of the Lys63/pSer65 peptide (K63pS65).AQUA analysis after 180 min as in (D) with Lys6-, Lys11-, Lys33-, Lys48-, Lys63-, and Met1-linked tetraUb (K6, K11, K33, K48, K63, M1). For all tetraUb chains, Ser65 is phosphorylated to greater than 80% after incubation with *Ph*PINK1 for 180 min.Time course analysis of autoubiquitination reactions with unphosphorylated full-length human Parkin (hParkin) and Ub (left), a 80% Ub/20% phosphoUb mixture (middle) and 100% phosphoUb (right).Analysis of different ratios of Ub:phosphoUb in a 120-min ubiquitination reaction of full-length hParkin. Parkin is activated by a mixture of Ub and phosphoUb, and is much less active in a reaction with no phosphoUb as well as with 100% phosphoUb.

Like others (Kane *et al*, 2014; Kazlauskaite *et al*, 2014; Koyano *et al*, 2014), we had also found that PINK1 is a Ub kinase while investigating the mechanism of Parkin activation (Fig1A). In an attempt to reconstitute Parkin-mediated ubiquitination of a reported substrate, Miro (Wang *et al*, 2011; Birsa *et al*, 2014), we performed a coupled kinase/ligase reaction with *Tribolium castaneum* (*Tc*) PINK1 (Kondapalli *et al*, 2012), human Parkin lacking the Ubl domain (Wauer & Komander, 2013), GST-Miro (residues 1–580), E1, UBE2L3 (UBCH7) and wild-type (wt) Ub. Tandem mass spectrometry on the reaction was searched for peptides containing phosphate incorporation. In these reactions, PINK1 phosphorylated Ub and targeted exclusively Ser65.

We confirmed that *in vitro*, *Pediculus humanus corporis (Ph)*PINK1 [related to *Tc*PINK1 (Kondapalli *et al*, 2012)] mediated phosphorylation of Ub proceeds to completion, while a Ub S65A mutant is not modified, as assessed by Phos-tag gels (Fig1B). Reactions with *Tc*PINK1 showed similar results and depended on active *Tc*PINK1 (Supplementary Fig S1A).

To generate large amounts of Ser65-phosphorylated Ub, 10 mg (1.15 μmol) of Ub was phosphorylated with enzymatic amounts of *Ph*PINK1. Phosphorylation proceeds to completion as assessed by electrospray ionization mass spectrometry (ESI-MS). Ub phosphorylation changes its isoelectric point from 6.6 to 5.7, enabling purification by anion exchange chromatography (see Materials and Methods). Purity of phosphoUb was confirmed by ESI-MS (Fig1C).

### PINK1 is a polyUb kinase

While this demonstrated that PINK1 phosphorylates monoUb, it was not clear whether PINK1 could also phosphorylate polyUb chains. Phos-tag gels of reactions using Lys63-linked triUb as substrate resulted in multiple bands and diffuse signals, suggesting that more than one Ub molecule can be targeted by *Ph*PINK1 in this chain type (Supplementary Fig S1B, C and E). For further analysis and quantification, we used AQUA-based mass spectrometry (Kirkpatrick *et al*, 2006; Ordureau *et al*, 2014). Digestion of a ubiquitinated protein with trypsin leads to a signature mass addition of 114 Da on the modified Lys, corresponding to the C-terminal GlyGly sequence of Ub. Spiking of tryptic digest mixtures with isotope-labeled GlyGly-modified standard peptides derived from each linkage site allows quantification of all chain types (Kirkpatrick *et al*, 2006). To account for the presence of phosphorylation at Ub Ser65, AQUA peptides incorporating pSer65 as well as (GG)Lys63/pSer65 were also included (see Supplementary Materials and Methods and Supplementary Table S1). With this, we followed phosphorylation of Lys63 hexaUb over time, revealing that all Ub molecules in the chain were phosphorylated by *Ph*PINK1 within 1 h (Fig1D). Lys63 chains adopt “open” conformation, while other chain types are more compact (Kulathu & Komander, 2012) and may not expose Ser65 for PINK1 phosphorylation. Still, we found that *Ph*PINK1 phosphorylated all Ub moieties in tetraUb with Met1, Lys6, Lys11, Lys33, Lys48 and Lys63 linkages (Fig1E and Supplementary Fig S1C–F), consistent with the dynamic nature of polyUb in solution (Ye *et al*, 2012). Hence, PINK1 is not only a Ub but also a polyUb kinase.

### PhosphoUb activates and inhibits Parkin

We confirmed reported roles for phosphoUb in Parkin activation. While full-length Parkin is inactive, addition of 20% purified phosphoUb led to robust activation in an *in vitro* auto-ubiquitination reaction (Fig1F). Assembled polyUb chains were detected with anti-Ub antibody (FK2), which recognizes Ub and phosphoUb similarly (Supplementary Fig S1G).

Surprisingly, when phosphoUb was used as the only source of Ub in the reaction, Parkin was hardly active and did not form polyUb. Parkin showed very low activity with 0 or 100% phosphoUb, but polyUb chain generation was triggered by small amounts of phosphoUb in the reaction. Increasing the phosphoUb ratio diminished Parkin activity (Fig1G). This unexpected behavior has also been reported by the Harper lab (Ordureau *et al*, 2014). It is known that the phosphoUb-mediated activation of Parkin proceeds with a Ub mutant that cannot proceed through E1/E2/E3 cascades, pointing toward an allosteric mechanism (Koyano *et al*, 2014). The fact that phosphoUb is seemingly unable to be assembled into polyUb by Parkin *in vitro*, suggests that phosphorylation affects Ub structure and/or affects its ability to be passed through the ubiquitination cascade.

### Ser65 phosphorylation changes Ub structure

**Figure 2:**
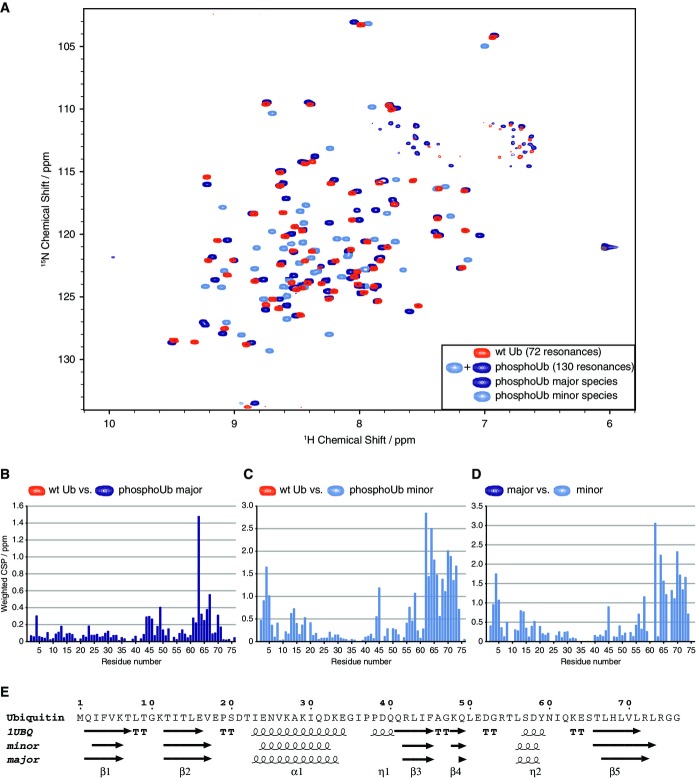
NMR analysis of phosphoUb
A ^1^H, ^15^N-BEST-TROSY spectrum of phosphoUb (shades of blue) overlaid with wild-type (wt) Ub (orange). The phosphoUb spectrum contains 130 non-sidechain resonances that have been colored in dark blue for the major species and light blue for the minor species based on the assignment of each species in Supplementary Fig S2B–D.B-D Weighted chemical shift perturbation (CSP) graphs for (B) wt Ub versus major phosphoUb species, (C) wt Ub versus minor phosphoUb species, (D) major versus minor phosphoUb species.E Comparison of Ub secondary structure [derived from pdb-id 1ubq (Vijay-Kumar *et al*, 1987)] with the secondary structure predictions of major and minor phosphoUb forms as calculated from backbone chemical shifts using TALOS+ (Supplementary Fig S5).

We first assessed biophysical and structural properties of phosphoUb. Ub is dynamic in solution yet generates excellent high-resolution NMR spectra with typically ∽72 resonances (Lange *et al*, 2008; Vögeli *et al*, 2009). To understand whether and how Ser65 phosphorylation affects Ub structure, we performed a phosphorylation reaction *in situ*, by adding *Ph*PINK1/MgATP to ^15^N-labeled Ub in a time-dependent NMR experiment, which led to complete and specific Ser65 phosphorylation as confirmed by ESI-MS (see Supplementary Materials and Methods). To our astonishment, the 2D-NMR spectrum of phosphoUb showed 72 resonances that are similar to wt Ub and in addition 58 new resonances (Fig2A). This suggested that phosphorylation of Ub at Ser65 generates two non-identical Ub conformations. These results were corroborated using purified ^13^C, ^15^N-labeled phosphoUb and it was possible to assign all resonances (Supplementary Fig S2). PhosphoUb in solution exists as a major species (70% as determined by relative peak intensities) that resembles wt Ub with resonances perturbed in the vicinity of pSer65 (Fig2B). In addition, we observed a minor species (30%) with significant chemical shift perturbations (CSPs) as compared to wt Ub (Fig2C) or to the major phosphoUb species (Fig2D). ZZ-exchange measurements revealed that the two species are in a slow equilibrium with a conversion rate of ∽2 per second (Supplementary Figs S3 and S4).

The second, minor species of phosphoUb has to our knowledge not been observed before. Secondary structure calculations for the major and minor species using TALOS+ (Fig2E, Supplementary Fig S5) revealed a similar secondary structure to wt Ub, indicating no global conformational changes or significant unfolding of the Ub molecule (Fig2E) (see below).

To test whether the conformational change was restricted to monoUb, we characterized phosphorylated, ^13^C, ^15^N-labeled Lys63-linked polyUb, which due to its open conformation displays BEST-TROSY spectra that are unperturbed by interface formation and are almost identical to Ub (72 peaks; Thr9, Glu24 and Arg74 resonances are exchange-broadened, while Lys63 and Glu64 resonances are split due to proximal and distal differences for these residues, and one extra peak for the isopeptide bond between the distal Gly76 and the side chain of the proximal Lys63). We found that phosphorylation also generated a minor conformation in uniformly labeled Lys63-linked di-, tri- and tetraUb (Supplementary Fig S6 and data not shown) with 128 peaks in phosphorylated Lys63-linked diUb. This indicates that phosphorylation-induced conformational changes can take place in the context of polyUb. Future work will need to assess whether the conformational changes exist in distal, proximal or both Ub moieties, whether it occurs in all chain types, and whether this leads to changes to polyUb structure.

### Phosphorylation affects Ub stability

Ub is highly stable and structurally unaffected by low pH, SDS or high temperatures [see e.g. (Ibarra-Molero *et al*, 1999)]. However, a novel conformation of Ub and the observed conformational transition could result in a less stable molecule. Indeed, we found that melting temperatures were decreased by 10°C in differential scanning calorimetry (DSC) experiments, from 93°C for Ub to 83°C for phosphoUb (Supplementary Fig S7). This is consistent with an alternative, less stable conformation being present, but also confirms that neither of these conformations is unfolded at the temperatures of NMR measurements. However, it does suggest that thermal denaturation of phosphoUb occurs earlier, and standard protocols of Ub purification should be used with care (Koyano *et al*, 2014).

### Crystal structure of phosphoUb

**Figure 3:**
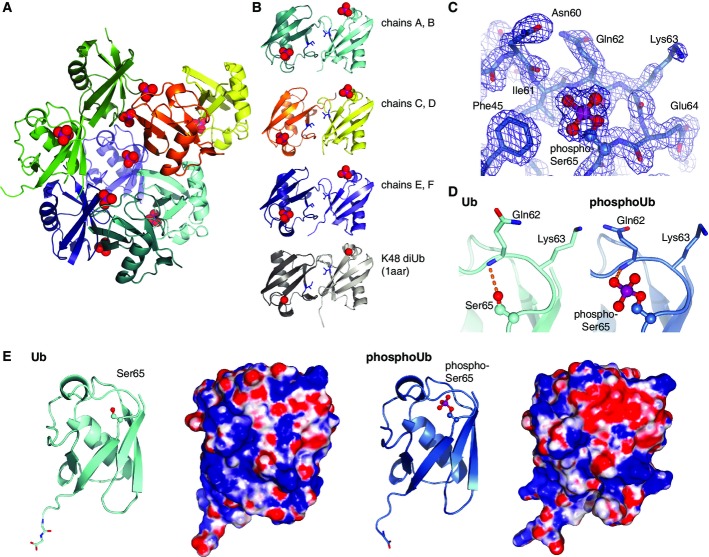
Crystal structure of Ser65-phosphoUb
Structure of phosphoUb in space group *P*1 with 8 molecules in the asymmetric unit (Table1). Ub molecules are colored differently, and atoms for pSer65 are shown as spheres with red oxygen and purple phosphorous atoms.Six of eight phosphoUb molecules form similar dimer interactions, where phosphoUb molecules interact via their Ile44 patch. This resembles the crystal structure of Lys48-linked diUb [pdb-id 1aar (Cook *et al*, 1992)]. Atoms for pSer65 are shown as in (A), and the Ile44 side chain is shown as blue sticks.Representative 2|F_o_|-|F_c_| electron density, contoured at 1 σ, covering the phosphorylation site. pSer65 is shown in ball-and-stick representation, and neighboring side chains are shown in stick representation with red oxygen and blue nitrogen atoms.In wt Ub (left), Ser65 forms a hydrogen bond with the backbone amide of Gln62. Upon phosphorylation (right), an oxygen atom from the phosphate forms the same backbone hydrogen bond.Cartoon representation and electrostatic surface potential of Ub (left) and phosphoUb (right) in identical orientations. Electrostatics were calculated using CHARMM (www.charmm-gui.org) and are colored from red (negative potential) to blue (positive potential).

The two conformations of Ub were analyzed in more detail. We obtained crystals of phosphoUb and determined a structure to 1.9 Å resolution (Table1). The new Ub crystal form (space group *P*1) contains eight phosphoUb molecules in the asymmetric unit (Fig3A). Six of the molecules interact similarly via their Ile44 patches, resembling structures of Lys48-linked diUb [pdb-id 1aar (Cook *et al*, 1992)] (Fig3B). Unambiguous electron density is observed for phospho-Ser65 in all molecules (Fig3C). The phosphate group forms a hydrogen bond with the backbone amide of Gln62, but no other contacts (Fig3D). In wt Ub, the same hydrogen bond is formed via the hydroxyl group of Ser65 (Fig3D). The small CSP for Gln62 in the major NMR species compared to wt Ub (Fig2B) is consistent with the conservation of the hydrogen bond of this backbone amide. This and the fact that there are no significant conformational differences between the phosphoUb structures in the crystal and a Ub reference structure [pdb-id 1ubq, RMSDs 0.45–0.69 Å (Vijay-Kumar *et al*, 1987)] indicated that the major phosphoUb NMR species had crystallized. While there are no conformational changes, phosphorylation of Ser65 leads to significant changes in the electrostatic surface potential of Ub (Fig3E), which may alter interactions with other proteins (see below and Discussion).

**Table 1:** Data collection and refinement statistics

	Ser65-phosphoUb
Data collection	
Space group	*P*1
Cell dimensions	
*a*, *b*, *c* (Å)	49.56, 53.29, 56.87
α*,* β*,* γ (°)	96.86, 104.94, 110.96
Resolution (Å)^a^	32.28–1.90 (1.94–1.90)
* R*_merge_^a^	0.124 (0.425)
* I*/σ*I*^a^	4.6 (2.1)
Completeness (%)^a^	97.3 (96.3)
Redundancy^a^	1.8 (1.8)
Refinement	
Resolution (Å)	32.28–1.90
No. reflections/test set	39,071/1,904
* R*_work_/*R*_free_	0.193/0.238
No. atoms	
Protein	4,579 (587 aa)
Ligand/ion	35
Water	356
*B*-factors	
Protein	27.3
Ligand/ion	45.8
Water	35.4
R.m.s. deviations	
Bond lengths (Å)	0.007
Bond angles (°)	1.00

a Values in parentheses are for highest resolution shell.

### Characterization of the minor phosphoUb species

As crystallization of phosphoUb did not provide information on the more intriguing minor species of phosphoUb, this was further investigated using a variety of solution NMR techniques. While the significant CSPs of the minor species compared to wt Ub and major phosphoUb species suggest substantial changes, the secondary structure prediction showed only small changes (Fig2E). It was unlikely that N- or C-terminal regions of Ub were unfolded, as this would lead to loss of β-sheet character in TALOS+ calculations (Supplementary Fig S5). Additionally, hetNOE measurements were collected to observe changes in local stability of the secondary structure (Supplementary Fig S8). Differences in motion on the pico- to nano-second timescale are usually seen at the termini of proteins, resulting in reduced or negative hetNOE values due to increased flexibility and motion. Resonances of the first β-strand (aa 2–6) have lower hetNOE values in the minor species as compared to the major species, indicating increased flexibility. Interestingly, Arg74 is significantly stabilized in the minor species. In wt Ub, this residue is part of the Ub C-terminal tail (aa 74–76) and does not interact with the Ub core but is flexible in solution (Lange *et al*, 2008). The observed stabilization of Arg74 in the minor species is consistent with our secondary structure prediction that calculates the β5-strand to be extended by one residue (Fig2E).

**Figure 4:**
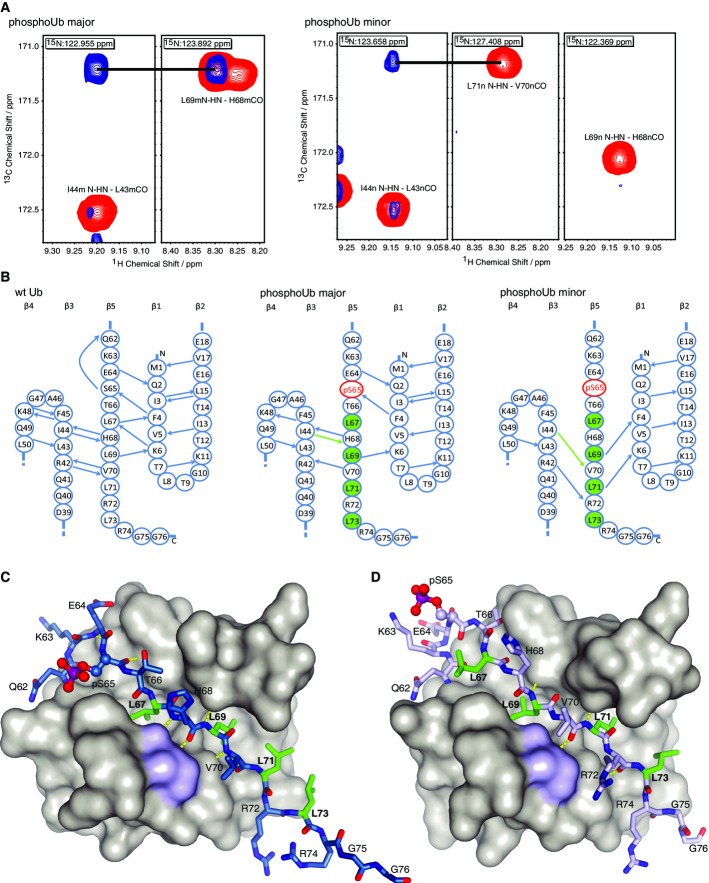
The minor conformation of phosphoUb
Long-range HNCO experiments showing differences of hydrogen bonding patterns for major and minor phosphoUb species. Through H bond correlations (blue) recorded for residue Ile44 in the major and minor species of phosphoUb with reference to a control HNCO spectrum (red). In the major form of phosphoUb (left), the H_N_ of Ile44 is a H bond donor to His68, as seen in wt Ub. In the minor form of phosphoUb (right), the H_N_ of Ile44 does not have a cross peak to His68 but instead has a correlation to Val70.Comparison of hydrogen bonding patterns for the β-sheet portion of wt Ub [left, according to Nisius and Grzesiek (2012)], the major form of phosphoUb (middle) and the minor form of phosphoUb (right). Four Leu residues (green) undergo a two-residue strand slippage to satisfy the observed hydrogen bonding pattern in the minor species. The green arrows indicate the resonances exemplarily shown in (A).Structure of the major form of phosphoUb, where phosphoUb residues 1–61 are shown under a surface, and the β5 strand is shown in stick representation with red oxygen, blue nitrogen and purple phosphorus atoms. The four core Leu residues are shown in green.Model of the phosphoUb minor species as in (C) in which the β5 strand is retracted into the protein core by two residues. This extends the β4–β5 loop and significantly shortens the C-terminal tail of Ub.

One explanation for the significant CSPs in the minor form and overall intact Ub structure could be dramatic changes in the hydrogen bonding pattern, which would affect the chemical environment of the entire Ub core. This was investigated using long-range HNCO experiments (Cordier *et al*, 2008; Nisius & Grzesiek, 2012). In a standard HNCO spectrum, the HN of a residue is correlated with the C′ resonance of the preceding residue via direct one-bond ^1^*J*_NC′_ coupling. By adjusting the HNCO pulse sequence for prolonged N-C′ magnetization transfer (total transfer time increased to 133 ms), weak ^h3^*J*_NC′_ couplings across hydrogen bonds can be recorded and assigned with reference to a standard HNCO spectrum (Fig4A). This analysis yielded a hydrogen bonding network for the β-sheet region of both phosphoUb species (Fig4B). The major form of phosphoUb has a similar pattern to that of wt Ub published previously (Nisius & Grzesiek, 2012) although some correlations could not be resolved (Fig4B, compare left and middle). The minor species of phosphoUb maintains similar contacts between β-strands β1 and β2 and partially between strands β3 and β4, suggesting that these core interactions are unaffected by Ub phosphorylation. Strikingly, the hydrogen bonding pattern of the β-strand β5 to both β3 and β1 is entirely different (Fig4B, compare middle and right). None of the contacts of wt Ub or phosphoUb major species are maintained, but a distinct set of contacts are formed between Ile44-Val70, Arg42-Arg72, Leu69-Phe4 and Leu71-Lys6. These contacts are beyond hydrogen bonding distance in any Ub molecule structurally resolved to date, suggesting that the β5-strand has shifted with respect to the Ub core to form the observed hydrogen bonds.

These results are corroborated by through space NOE analysis (Supplementary Fig S9), which confirmed that the major conformation had contacts that were consistent with the crystal structure and that the minor conformation had a shifted β5 strand as suggested by the long-range HNCO experiment.

### Ser65 phosphorylation induces β5-strand slippage in phosphoUb

Closer inspection of Ub structure and sequence reveals that such a β-strand slippage is entirely feasible. The β5-strand has four Leu residues (Leu67, Leu69, Leu71 and Leu73), and Leu67 and Leu69 are integral to the Ub core (Fig4B and C). The intermittent residues His68, Val70 and Arg72 are solvent-exposed (Fig4C). Modeling of a β-strand slippage of two residues, that is, replacing Leu67 with Leu69, Leu69 with Leu71, etc., reveals a model for the minor phospho-Ub species in which the C-terminal tail of Ub has retracted into the Ub core by two amino acids (Fig4D). The generated model is consistent with all data: (i) The four measured long-range HNCO contacts are now satisfied (Fig4A, B and D), (ii) the through space NOE contacts are reconciled (Supplementary Fig S9), (iii) retraction of the C-terminal tail would lead to core contacts and stabilization of Leu73 and Arg74 as observed in the hetNOE experiment (Supplementary Fig S8), (iv) the C-terminal tail would extend the β5-sheet, as was predicted in the secondary structure analysis (Fig2E, Supplementary Fig S5) and (v) all residues in β5, the preceding loop (aa 62–66), in β1 (aa 2–7) and in β3 (Ile44 and Phe45) have changed their environment significantly, explaining the large chemical shift perturbations (Fig2A–D). The largest CSP in the minor species occurs for Gln62 (Fig2C), indicating a drastically altered environment around the Gln62 amide, possibly due to loss of the conserved hydrogen bond of the backbone amide (see above).

Retracting the Ub C-terminus would extend the phospho-Ser65 containing loop (aa 62–66), which in the hetNOE data appears to be rigid, suggesting that pSer65 forms new interactions on the Ub surface. Likewise, retraction of the important Ub C-terminus would affect interactions with most enzymes in the Ub system. Clearly, this model for a new Ub conformation requires further structural validation, which could be obtained by identifying binding partners or mutations that stabilize this species (see Discussion).

Together, the data show that phosphorylation has dramatic effects on Ub structure. It changes the surface properties of the major species, and, more intriguingly, generates a previously unobserved Ub conformation (which we will refer to as the Ub^retraCT^ conformation). Ser65 phosphorylation leads to a β-strand slippage by two residues, which is enabled by the sequence of the β5-strand. These changes in Ub structure may have many repercussions on the Ub system, which relies on highly conserved and regulated interactions of Ub with the many enzymes facilitating its attachment and removal.

### Effects of phosphoUb on the Ub system

Ub is almost invariant in evolution, is identical in higher eukaryotes and differs only by one non-conservative and two conservative mutations between *Saccharomyces cerevisiae* and humans. PINK1 from all species tested to date phosphorylates the invariant Ub Ser65, suggesting that the gain-of-function of phosphoUb in Parkin activation and mitophagy is evolutionarily conserved. However, Ub plays many roles, and it is currently unclear whether phosphoUb has other, potentially detrimental effects on the Ub system. The structural data described above (Figs4) show that phosphoUb is structurally distinct and could potentially adopt new functions. For example, we posit that the minor species could be recognized by as-yet unknown phosphoUb binding proteins. It is also clear that in the major phosphoUb species, the main Ub interactions sites, the Ile44 and the Ile36 patches, are structurally intact and extensively utilized in crystal lattice interfaces (Fig3). Nonetheless, all surfaces of Ub are highly conserved, and most have been implicated in protein interactions during Ub chain assembly, binding and/or disassembly.

### Consequences of phosphoUb on E2 charging and discharging

**Figure 5:**
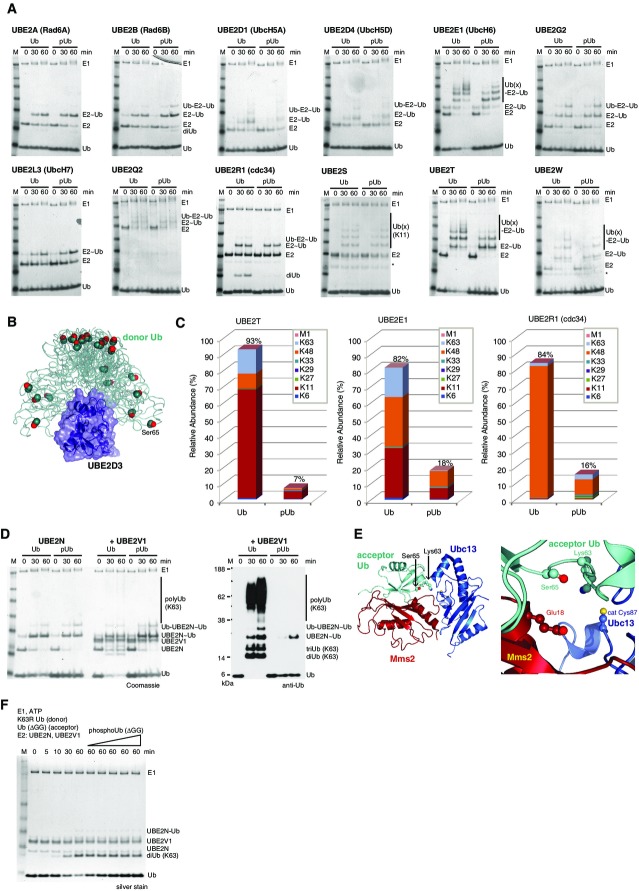
Impact of Ub phosphorylation on E1 and E2 enzymes PhosphoUb is abbreviated to pUb in this Figure.
E1-mediated charging of E2 enzymes by Ub and phosphoUb in a time-course analysis. Non-reducing Coomassie-stained 4–12% SDS–PAGE gradient gels are shown, and bands are labeled. “˜Ub” refers to generation of a thioester, while “Ub-“refers to additional covalent ubiquitination of the E2 enzyme. * contaminant from E2 purification.SAXS-derived ensemble of UBE2D3 (Pruneda *et al*, 2011), where the E2 is shown under a blue surface and the Ub is shown as a green ribbon. Ser65 is shown in sphere representation. Ser65 does not contact the E2 enzyme in any orientation of the Ub at the E2 enzyme. PDB files were downloaded from the Klevit-lab website (http://depts.washington.edu/klvtlab/).Quantitative mass spectrometry analysis for UBE2T, UBE2E1 and UBE2R1 with Ub and phosphoUb using Ub AQUA peptides reveals impaired chain formation with phosphoUb (see also Supplementary Fig S10).Charging of UBE2N, and UBE2N/UBE2V1-mediated Lys63 chain assembly with Ub and phosphoUb. Charging of UBE2N as in (A) proceeds identically with both Ub and phosphoUb (left 6 lanes), while UBE2V1-mediated Lys63 chain assembly only proceeds with Ub but not phosphoUb (right 6 lanes and Western blot).Structure of *Saccharomyces cerevisiae* Ubc13-Mms2 (blue/red, Mms2 is a homolog of UBE2V1) with the acceptor Ub (cyan) bound non-covalently to Mms2 [pdb-id 2gmi (Eddins *et al*, 2006)]. Ser65 of the acceptor Ub interacts with Mms2, and Ser65 phosphorylation would prevent binding due to Mms2 Glu18.PhosphoUb competition assays for UBE2N/UBE2V1-mediated Lys63 diUb formation. Time course with K63R Ub (donor) and Ub ΔGG (acceptor) generates Lys63 diUb and is not inhibited upon increasing concentrations of phosphoUb, revealing phosphoUb does not compete with Ub for UBE2N/UBE2V1-mediated Lys63 diUb formation.

The E1-mediated charging of E2 enzymes is a sophisticated multi-step reaction in which Ub is engaged in a variety of ways (Schulman & Harper, 2009). We screened 13 of the 19 human E2 enzymes that are charged by the Ub E1 UBA1, to find that all tested enzymes were charged by Ub and phosphoUb similarly (Fig5).

The E1-generated E2∽Ub conjugate is relatively stable until discharged by a ligase. In NMR and SAXS ensembles of charged E2 enzymes, Ub is flexibly attached to the E2 and can take many conformations (Pruneda *et al*, 2011). Importantly, Ser65 is remote from the active site and does not interact with the enzyme (Fig5B, Supplementary Fig S10A), suggesting that the E2∽phosphoUb conjugate may behave similarly to an E2∽Ub conjugate.

A subset of E2 enzymes ubiquitinated themselves on one or multiple sites *in vitro*, and this was in most cases independent of Ub phosphorylation (Fig5A). We also noted considerable auto-polyubiquitination of UBE2E1 and UBE2T in our assays. UBE2S and UBE2R1 (cdc34) assemble free Ub chains linked via Lys11 and Lys48, respectively. Interestingly, while UBE2S assembled Lys11-linked chains regardless of whether Ub was phosphorylated or not, UBE2R1, UBE3E1 and UBE2T were significantly impaired in discharging and chain formation when phosphoUb was used in the reaction (Fig5A and C, Supplementary Fig S10B–D). This was quantified by AQUA mass spectrometry indicating GlyGly-modified Ub peptides in E2 reactions with phosphoUb were significantly reduced (Fig5C, Supplementary Fig S10B–D). Hence, while UBE2R1, UBE2T and UBE2E1 were charged with phosphoUb (Fig5A), they were unable to generate significant levels of phosphoUb chains.

The most dramatic effects of phosphoUb on E2 chain elongation were observed with UBE2N (UBC13), which in complex with the inactive E2-fold proteins UBE2V1 (UEV1A) or UBE2V2 (MMS2) assembles free Lys63-linked polyUb. UBE2N was charged identically with Ub or phosphoUb as observed for the remaining E2s (Fig5A and D). Addition of UBE2V1 led to robust Lys63 chain assembly with Ub, but the enzyme complex was inactive with phosphoUb (Fig5D). This finding can be explained structurally from the complex of *S. cerevisiae* Ubc13∽Ub with Mms2 (Eddins *et al*, 2006). The acceptor Ub is bound by Mms2, which positions it such that Lys63 points toward the active site. Ser65 of the acceptor Ub interacts directly with Mms2, and although the side chain does not make any contacts, phosphorylation would clash with Glu18 of Mms2 (Fig5E). All functionally important residues in Ubc13/Mms2 are invariant in human UBE2N/UBE2V1. A reaction with Ub K63R as a donor Ub and Ub lacking the C-terminus (Ub ΔGG) as an acceptor Ub assembled Lys63-linked diUb specifically (Fig5F). Increasing amounts of phosphoUb ΔGG did not affect diUb generation (Fig5F), suggesting that this species was unable to compete with Ub ΔGG for UBE2V1 binding. Hence, the defect in UBE2N/UBE2V1 chain assembly is due to blocked acceptor Ub binding, consistent with the structure.

In summary, E2 charging with phosphoUb is unaffected for the majority of E2 enzymes, showing that E1 does not differentiate between Ub and phosphoUb. Importantly, some E2 enzymes are impaired in polyUb chain formation with phosphoUb, and, for example, the UBE2N/UBE2V1-mediated assembly of Lys63-linked chains is switched off by phosphoUb.

### Effects of phosphoUb on E3-mediated ubiquitination

E2 enzymes are discharged with the help of E3 ligases, which either induce a high-energy state of the E2∽Ub complex (RING, U-box E3 ligases) or accept Ub to form a second thioester intermediate (HECT, RBR E3 ligases) (Berndsen & Wolberger, 2014). Recent structural work has revealed how RING E3 ligases generate a spring-loaded E2∽Ub complex (Dou *et al*, 2012; Plechanovova *et al*, 2012; Pruneda *et al*, 2012), and Ser65 of Ub is not involved in E2 or E3 interactions in any of the studied trimeric complexes (Supplementary Fig S11A). Likewise, Ub Ser65 is not contacted in HECT and RBR Ub complex structures (Supplementary Fig S11B and C). However, as seen with E2 enzymes, a switch from Ub to phosphoUb could lead to differences in ligase activity due to effects on acceptor Ub binding or may change linkage composition during chain formation.

**Figure 6:**
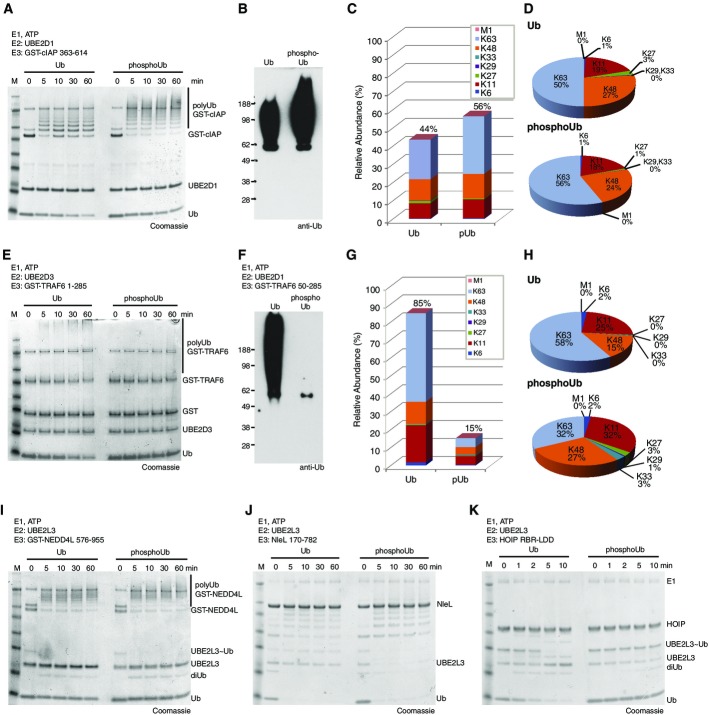
E3-mediated autoubiquitination with Ub and phosphoUb
A Time course for a ligase reaction with GST-tagged cIAP1 (aa 363–614), UBE2D1 and Ub or phosphoUb. See Supplementary Fig S11D for a reaction with UBE2D3.B Anti-Ub Western blot of an identical reaction run for 3 h.C Comparison of the number of Ub chain linkages in the reaction in (B).D Ub chain linkage profile for the reaction in (B).E Reaction of GST-tagged TRAF6 (aa 1–285) with UBE2D3 as in (A). See Supplementary Fig S11E and F for additional TRAF6 reactions.F Anti-Ub Western blot of GST-TRAF6/UBE2D1 run for 3 h.G Comparison of the number of Ub chain linkages in the reaction in (F).H Linkage composition in GST-TRAF6-UBE2D1 reactions reveals 58% Lys63 chains with wt Ub and UBE2D1. Significantly smaller quantities of linkages are assembled with phosphoUb.I-K Chain assembly as in (A) with UBE2L3 and (I) GST-tagged NEDD4L (aa 576–955) (Mund & Pelham, 2009), (J) NleL (aa 170–782) (Hospenthal *et al*, 2013) and (K) HOIP RBR-LDD (aa 699–1072) (Smit *et al*, 2012). While the HECT E3 ligases NEDD4L and NleL are active with Ub and phosphoUb, HOIP is significantly less active with phosphoUb. See also Supplementary Fig S11G and H.

We tested GST-tagged RING domains of cIAP1 (aa 363–614) and TRAF6 (aa 1–285 and 50–285) in autoubiquitination reactions with Ub- or phosphoUb-charged UBE2D1 and UBE2D3 (Yang *et al*, 2005). UBE2D1 and UBE2D3 work with most E3 ligases, are non-specific for substrate modification and assemble multiple linkage types *in vitro* (Kim *et al*, 2007; Dynek *et al*, 2010). Autoubiquitination of GST-cIAP1 proceeded with Ub and phosphoUb similarly with either E2 enzyme, leading to a depletion of monoUb and to qualitatively similar chain laddering on GST-cIAP1 (Fig6A and B, Supplementary Fig S11D). AQUA mass spectrometry revealed that GST-cIAP1/UBE2D1 assembled chains to a similar extent (Fig6C) and with similar composition with Ub and phosphoUb, namely Lys63 (∽50%), Lys48 (∽25%), Lys11 (∽18%), and Lys6 and Lys27 linkages in smaller amounts (Fig6D). The high amounts of Lys63 linkages (> 50% in both samples) were interesting, as this contrasts the finding of UBE2N/UBE2V1 where this linkage type was no longer assembled (Fig5D). Hence, phosphoUb has no impact on cIAP1-mediated chain assembly.

Surprisingly, with TRAF6, the same E2 enzymes, UBE2D1 and UBE2D3, assembled chains processively with Ub but not with phosphoUb (Fig6E and F). This was observed for several GST-TRAF6 constructs that include the RING domain as well as zinc finger modules (Fig6E and F, Supplementary Fig S11E). AQUA mass spectrometry confirmed this result, showing that only a small fraction of phosphoUb is involved in chain linkages (Fig6G and H). The low activity with phosphoUb was TRAF6 dependent (Supplementary Fig S11F). These results are unexpected as they suggest that TRAF6 is able to recognize the Ser65 phosphorylation status.

To understand whether phosphoUb affects other ligase classes, we tested NEDD4L, a Lys63-specific enzyme (Kamadurai *et al*, 2009) with the E2 enzyme UBE2L3 that specifically transfers Ub onto the catalytic Cys of HECT and RBR E3 ligases (Wenzel *et al*, 2011). NEDD4L/UBE2L3 assembled Ub chains with Ub and phosphoUb identically (Fig6I). Also, the bacterial effector HECT-like enzyme NleL (Hospenthal *et al*, 2013) assembled chains similarly with Ub and phosphoUb (Fig6J, Supplementary Fig S11G).

Finally, we tested another member of the RBR E3 ligase family, HOIP, which is part of the linear Ub chain assembly complex (LUBAC) (Kirisako *et al*, 2006). The HOIP RBR domain extended C-terminally by a Ub binding domain assembles Met1-linked chains processively *in vitro* (Smit *et al*, 2012; Stieglitz *et al*, 2012). Interestingly, this reaction proceeded with Ub, but was inhibited with phosphoUb (Fig6K, Supplementary Fig S11H). The structural basis for this is unclear. A structure of a minimal HOIP construct sufficient for Met1-chain assembly bound to two Ub molecules (Stieglitz *et al*, 2013) showed that Ser65 is solvent-exposed in both donor and acceptor Ub (Supplementary Fig S11C). This suggests that the extended construct used in the assay forms additional Ub interactions involving Ser65 of donor or acceptor Ub that cannot be formed when Ser65 is phosphorylated. Alternatively, the phosphate affects either access to or the *pK*_*A*_ of the acceptor α-amine of Met1.

In summary, although testing only a small subset of E3 ligases, we uncovered that phosphoUb can lead to marked defects in polyUb chain generation.

### Attributing phosphoUb ligation defects to the major conformation

Known structures of the ubiquitination machinery are incompatible with the observed minor phosphoUb^retraCT^ conformation. An interesting finding with phosphomimetic Ub mutants confirmed that the surface charge changes of the major conformation in phosphoUb accounted for the defects in the ligation deficiency in TRAF6/UBE2D and UBE2N/UBE2V1.

Parkin activation in cells could be mimicked by phosphomimetic Ub mutants, where the phosphorylated Ser65 is replaced with Asp or Glu (Kane *et al*, 2014; Koyano *et al*, 2014). We tested whether phosphomimetic mutants displayed two conformations in NMR experiments, by analyzing spectra of ^15^N-labeled Ub S65E and S65D (Supplementary Fig S12A). Both mutants did not show the additional peaks of the Ub^retraCT^ conformation, but revealed a spectrum with 69 resonances (Thr9, Glu24 and Ala46 were exchange-broadened as compared to Ub) resembling the major conformation of phosphoUb (Supplementary Fig S12B). This is important as it suggests that phosphomimetic Ub mutants may not reconcile all effects of Ub Ser65 phosphorylation, and results from cell biology studies should be interpreted with care.

Still, this result was useful as it enabled us to understand whether the phosphoUb^retraCT^ conformation or the major phospho-Ub conformation accounted for the observed defects in chain assembly. Indeed, consistent with structural considerations (Fig2E), Ub S65E is unable to perform ubiquitination with UBE2N/UBE2V1 (Supplementary Fig S13A). Similarly, for TRAF6/UBE2D, Ub S65E significantly inhibited chain assembly, although not to the same extent as phosphoUb (Supplementary Fig S13B). There could be multiple reasons for the difference, and it is possible that the conformational equilibrium of phosphoUb may exaggerate the observed loss of activity.

### Lys63-linked phosphoUb chains are recognized by TAB2

**Figure 7:**
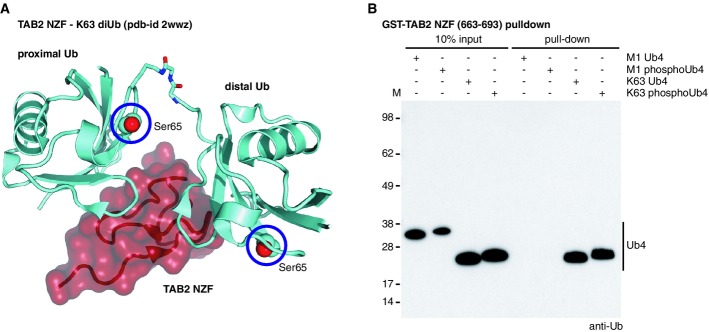
Binding of TAB2 NZF to phosphoUb chains
Structure of TAB2 NZF (red) in complex with Lys63 diUb (cyan) [pdb-id 2wwz (Kulathu *et al*, 2009)]. Ser65 for both Ub moieties is shown as spheres and highlighted with a blue circle.Pull-down assays with the Lys63-specific TAB2 NZF against different tetraUb (Ub4) species are shown. Phosphorylation of K63 Ub4 does not prevent binding to TAB2 NZF. Met1-linked tetraUb does not interact with TAB2 NZF, independent of Ser65 phosphorylation.

Ubiquitin binding domains (UBDs) decode Ub signals, and some preferentially interact with Ub polymers (Husnjak & Dikic, 2012). As mentioned above, the principle UBD recognition site on Ub, the Ile44 patch, is not grossly affected in the major conformation of phosphoUb, and we would expect small UBDs to interact similarly with Ub and phosphoUb. The case may be different for chain interacting UBDs. The TAB2 NZF domain “bends” a Lys63-linked diUb to interact with its two Ile44 patches simultaneously (Kulathu *et al*, 2009; Sato *et al*, 2009), yet Ser65 of either molecule is solvent-exposed (Fig7A).

To test whether TAB2 binds poly-phosphoUb, we performed pull-down experiments using GST-tagged TAB2 NZF domain (Kulathu *et al*, 2009), revealing strong interactions with Lys63-linked but not Met1-linked tetraUb regardless of phosphorylation status (Fig7B). TAB2 has previously been used to sense Lys63-linked chains on mitochondria after depolarization (van Wijk *et al*, 2012), and it can be assumed that the detected chains were partly phosphorylated.

### DUBs hydrolyze phosphoUb chains with lower activity

**Figure 8:**
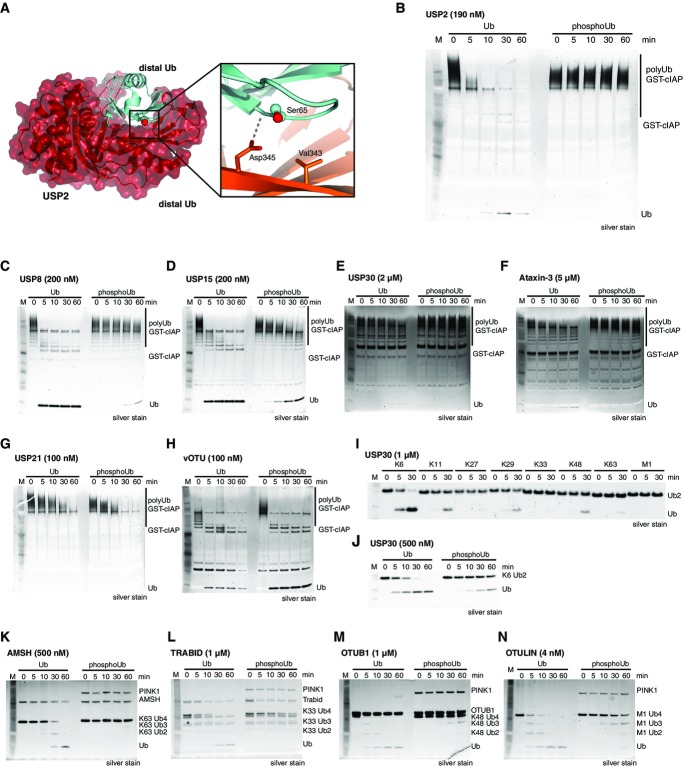
Defects in hydrolyzing phosphoUb chains
A Structure of USP2 (red) bound to Ub (cyan) [pdb-id 2hd5 (Renatus *et al*, 2006)], with a close-up on Ub Ser65 in the distal Ub. A negative charge at the USP2 Asp345 position is conserved in most USP enzymes (Ye *et al*, 2009).B-H Ubiquitination reactions from Fig6A were used as input for deubiquitinase (DUB) analysis. Silver-stained time-course gels are shown. All DUBs tested (USP2, USP8, USP15, USP30, Ataxin-3, USP21) hydrolyze Ub chains but have significantly lower activity against phosphoUb chains, with the exception of vOTU and USP21.I USP30 activity was significantly lower for cIAP polyUb as compared to other USP DUBs. Cleavage assays against the diUb panel reveals preference for Lys6-linked chains.J USP30 activity is decreased against phosphorylated Lys6 diUb.K-N Deubiquitinase assays against phosphorylated tetraUb. The activity of AMSH (K), TRABID (L), OTUB1 (M) and OTULIN (N) are inhibited with Lys63, Lys33, Lys48 and Met1 phosphorylated tetraUb, respectively.

Finally, we analyzed the ability of DUBs to hydrolyze phosphoUb chains. The human genome encodes ∽80 active DUBs, > 50 of which contain Ub-specific protease (USP) domains. The remaining enzymes belong to four structurally distinct families (Komander *et al*, 2009; Clague *et al*, 2013). USP enzymes such as USP2 (Fig8A) engulf ∽40% of the solvent accessible surface of a bound distal Ub (Komander *et al*, 2009). Importantly, Ser65 is involved in these interactions, and phosphorylation may affect DUB recognition (Fig8A). Indeed, when we tested USP2 activity against ubiquitinated GST-cIAP1 (Fig6A) as a substrate in DUB assays, we found that USP2 was markedly less active against poly-phosphoUb (Fig8B) and only cleaved phosphoUb chains at later time points or at higher enzyme concentration (data no shown). Identical behavior was found for USP8, USP15 and USP30 (Fig8C–E). Also, the Josephin-family DUB Ataxin-3 showed less activity against poly-phosphoUb (Fig8F). Interestingly, USP8, USP15, USP30 and Ataxin-3 have all been implicated in Parkin regulation (Durcan *et al*, 2012, 2014; Bingol *et al*, 2014; Cornelissen *et al*, 2014). In contrast, known non-specific enzymes USP21 and the small viral OTU domain DUB vOTU (Akutsu *et al*, 2011; Ye *et al*, 2011) were able to cleave polyUb and poly-phosphoUb similarly (Fig8G and H).

USP30 was significantly less active toward cIAP polyUb chains as compared to other USP DUBs. To test whether this was due to a different chain preference, we performed a specificity analysis against a panel of differently linked diUb, revealing that USP30 preferred Lys6-linked chains (Fig8I). This is interesting as Lys6-linkages are enriched in Parkin substrates (Durcan *et al*, 2014; Ordureau *et al*, 2014). USP30 was still less active toward phosphorylated Lys6-linked diUb (Fig8J).

Finally, we tested the activity of linkage-specific DUBs against defined phosphorylated substrates. The JAMM-family DUB AMSH is Lys63-specific (McCullough *et al*, 2004), but is less active against phosphorylated chains (Fig8K). Similarly, Lys29/Lys33-specific TRABID, Lys48-specific OTUB1 and Met1-specific OTULIN are impaired in cleaving phosphorylated chains (Fig8L–N).

Structurally, some of these results are surprising. While USP Ub binding may explain the weak activity of this enzyme class against poly-phosphoUb, the outlier USP21 shows that subtle differences in the binding site apparently enable a USP domain to target phosphorylated chains (Supplementary Fig S14A). Ser65 in distal or proximal Ub is solvent-exposed in known structures of OTU DUBs, such as OTUB1 (Juang *et al*, 2012; Wiener *et al*, 2012) (Supplementary Fig S14B) and OTULIN (Keusekotten *et al*, 2013; Rivkin *et al*, 2013) (Supplementary Fig S14C). Similarly, Ser65 in the distal Ub is solvent-exposed in the Ataxin-3-like structure (Weeks *et al*, 2011) (Supplementary Fig S14D). In AMSH-LP, Ser65 of the proximal Ub is close to the enzyme (Sato *et al*, 2008) and may account for the observed reduced cleavage activity (Supplementary Fig S14E).

The fact that 10 out of 12 tested DUBs have notably weaker activity against chains that contain phosphoUb suggests that phosphorylated chains are more stable as compared to unphosphorylated chains. Importantly, chains can be phosphorylated by PINK1 (Fig1), and poly-phosphoUb exists in cells (Ordureau *et al*, 2014). This together with the inhibition of ligases could change the dynamics of modified substrates, their degradation rates or signaling capacity. Hence, Ub phosphorylation may have consequences on the dynamics of the entire Ub system.

## Discussion

PINK1-mediated phosphorylation of Ub at Ser65 has dramatic consequences for Ub structure, and key processes in the Ub system, namely Ub attachment and removal.

It could be expected that phosphorylation of Ub would change its surface properties due to the addition of a negative charge. The obtained high-resolution crystal structure and solution studies agree that the majority of phosphoUb is structurally similar to wt Ub. To our amazement, NMR studies showed a second, minor conformation of phosphoUb, which is in slow exchange with the major conformation. Strikingly, the minor conformation shows distinct hydrogen bonding patterns and long-range NOEs for its C-terminal β5-strand, which can only be structurally satisfied when this strand is shifted by two residues. Our phosphoUb^retraCT^ model explains numerous observations and is structurally feasible due to the existence of four Leu-Xaa repeats in the β5-strand that would allow a shift of two residues without significantly disturbing the Ub core. β-strand slippage is a known phenomenon occurring in, for example, Arf family GTPases (Pasqualato *et al*, 2002) and Serpins (Zeraik *et al*, 2014). In these proteins, strand slippage is induced by changes of nucleotide (GDP/GTP) in the binding pocket. While conceptually similar, we are not aware of a phosphorylation-induced β-strand slippage as reported here, and to our knowledge, the Ub^retraCT^ species has not been observed previously.

Structurally, β5-strand slippage would have significant implications: Firstly, shifts of His68 and Val70 would disrupt the important Ile44 hydrophobic patch which is involved in most Ub interactions described to date (Husnjak & Dikic, 2012; Komander & Rape, 2012). Secondly, the Ub^retraCT^ conformation can presumably not be recognized by enzymes of the assembly cascade (E1, E2, E3) as well as DUBs since these proteins rely on interactions with the extended Ub C-terminus. Thirdly, retraction of the C-terminus may change the dynamics and structure of polyUb chains, adding further complexity to the already vast structural landscape adopted by Ub polymers (Kulathu & Komander, 2012).

The relevance and potential roles of the Ub^retraCT^ conformation remain unclear. Its existence raises fundamental questions for Ub and Ubl conformation, dynamics and folding. Biologically, the Ub^retraCT^ conformation could be recognized as a distinct signal. One could speculate that a mitophagy adaptor or other phosphoUb binding proteins may preferentially recognize the Ub^retraCT^ conformation of phosphoUb, and it will be interesting to identify such proteins.

Biochemically, it was not clear whether phosphoUb can be assembled into chains and whether chains incorporating phosphoUb are recognized by UBDs and hydrolyzed by DUBs. Using representative members from all branches of the Ub assembly and disassembly cascades, we demonstrated that, in principle, E1 activation, E2 charging and E3-mediated discharging can progress with phospho-Ub, although we made several surprising observations. Most strikingly, the Lys63-specific assembly complex UBE2N/UBE2V1 was inactivated by phosphoUb, which can be explained structurally. PhosphoUb also affected discharging in other E2 enzymes; the reasons for this are less clear.

We also investigated two RBR, two HECT and two RING E3 ligases, uncovering surprising differences for the latter. With identical E2 enzymes, the well-studied RINGs of cIAP1 and TRAF6 processed Ub and phosphoUb differently, and our results suggest that TRAF6 recognizes Ub and is unable to bind phosphoUb. The two tested HECT E3 ligases worked similarly with Ub and phosphoUb, while the RBR enzyme HOIP and Parkin are less active with phosphoUb.

Ub phosphorylation severely affects DUB activity, and 5 out of 6 USP enzymes, 4 out of 5 OTU enzymes and the tested JAMM and Josephin DUBs were significantly less active against phosphoUb. We were surprised that all DUBs implicated in Parkin biology and mitophagy were less active with phosphoUb; we wonder whether a phosphoUb-specific DUB exists. Since phosphoUb chains are more resistant to DUB activity, they likely constitute a more stable Ub signal.

Our work also revealed some insights for the PINK1/Parkin system. We show that, at least *in vitro*, Ub tetramers comprising six of the eight linkage types can be phosphorylated at every Ub in the chain. Harper and colleagues showed that this likely holds true *in vivo*, where PINK1 phosphorylates polyUb of various chain linkages on mitochondria (Ordureau *et al*, 2014). This is interesting from a kinase perspective since, apparently, PINK1 is able to access each Ub molecule within chains to phosphorylate Ser65. Moreover, Ser65 is in a folded and structurally stable environment in Ub, not exposing any stretch of primary sequence. This indicates that PINK1 likely recognizes a three-dimensional motif present in Ub and the Parkin Ubl domain, rather than, as more common for kinases, a peptide motif.

According to proteomic analysis, PINK1 phosphorylates 2.5–3% of total Ub and 10–20% of mitochondrial Ub upon mitochondrial depolarization (Koyano *et al*, 2014; Ordureau *et al*, 2014). Different models have been proposed for phosphoUb-mediated Parkin activation, and the sequence of events is still debated (Zheng & Hunter, 2013; Kane *et al*, 2014; Kazlauskaite *et al*, 2014; Koyano *et al*, 2014; Ordureau *et al*, 2014). We here show that for phosphoUb to activate Parkin, unphosphorylated Ub must be present. This was also shown by Harper and colleagues, although in their case, the effects of pure phosphoUb appear less dramatic (50% reduction in chain formation) (Ordureau *et al*, 2014). The mechanistic basis for this is not clear and will need to be revealed in structural studies.

**Figure 9:**
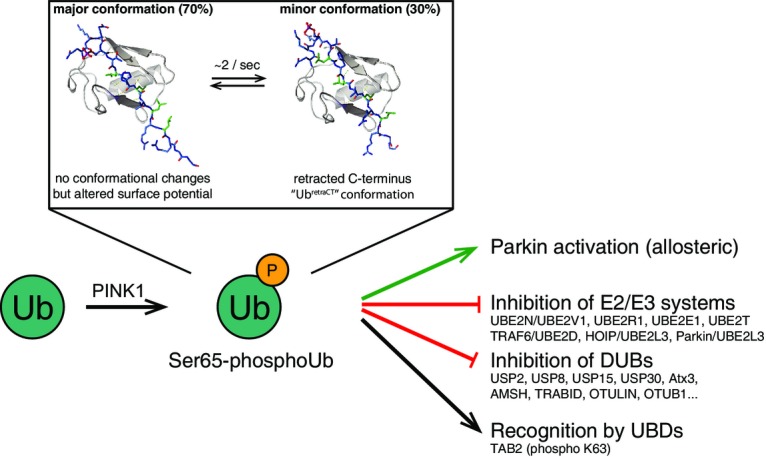
New roles for Ser65-phosphoUb Phosphorylation of Ub by PINK1 has multiple consequences. Structurally, it alters the electrostatic potential of Ub, but also generates a new Ub^retra^^CT^ conformation with a retracted C-terminus. The Ub^retra^^CT^ conformation is in slow exchange with the common Ub conformation. Functionally, Ub phosphorylation leads to a gain-of-function of Ub, as it becomes an allosteric activator of Parkin. We here show that phosphoUb also has loss-of-function effects, as it inhibits some assembly and disassembly systems. Additionally, it is possible that phosphoUb is recognized specifically by Ub receptors.

In summary, we show that phosphoUb, beyond the described gain-of-function in Parkin activation, has primarily detrimental effects on the Ub system, partly due to significant structural perturbations (Fig9). Many issues remain, in particular regarding specific recognition of phosphoUb by (novel?) UBDs, specific reversal of phosphoUb signals by DUBs, and regarding functional consequences from inactivating E2/E3 ligase systems. Structurally, it is of prime importance to understand how phosphoUb is recognized by Parkin, how PINK1 can phosphorylate Ub specifically, and whether the Ub^retraCT^ conformation has additional binding partners.

It is also clear that the identification of Ub phosphorylation by PINK1 is the tip of the iceberg with regard to Ub modifications. Several Ser/Thr/Tyr residues of Ub, including Thr7, Thr12, Ser57 and Tyr59, are known to be phosphorylated by as-yet unknown kinases (http://www.phosphosite.org), and all may have dedicated functions and pathways. This vastly increases the complexity in the Ub system and requires careful systematic future studies.

## Materials and Methods

Please find complete Materials and Methods in the Supplementary Information online.

### Data deposition

Crystallographic data for Ser65-phoshoUb have been deposited with the protein data bank, accession number 4wzp.

## Supporting Information

Supplementary Information

Review Process File
