# Supplementary material for: Ubiquitin Ser65 phosphorylation affects ubiquitin structure, chain assembly and hydrolysis
Source: EMBO J. 2014 Dec 20;34(3):307–25. doi: 10.15252/embj.201489847 (PMC4339119; doi:10.15252/embj.201489847)
Supplement: Supplementary file 1 [file embj0034-0307-sd1.pdf]

Ubiquitin Ser65 phosphorylation affects ubiquitin structure, chain assembly and hydrolysis

Supplementary Material

Tobias Wauer[#], Kirby N. Swatek[#], Jane L. Wagstaff[#], Christina Gladkova, Jonathan N. Pruneda, Martin A. Michel, Malte Gersch, Christopher M. Johnson, Stefan M.V. Freund and David Komander*

Medical Research Council Laboratory of Molecular Biology, Francis Crick Avenue, Cambridge, CB2 0QH, UK.

* Corresponding author: David Komander, dk@mrc-lmb.cam.ac.uk

[#] These authors contributed equally to this work

Running title: Ser65-phosphorylated ubiquitin

Supplementary Figure 1

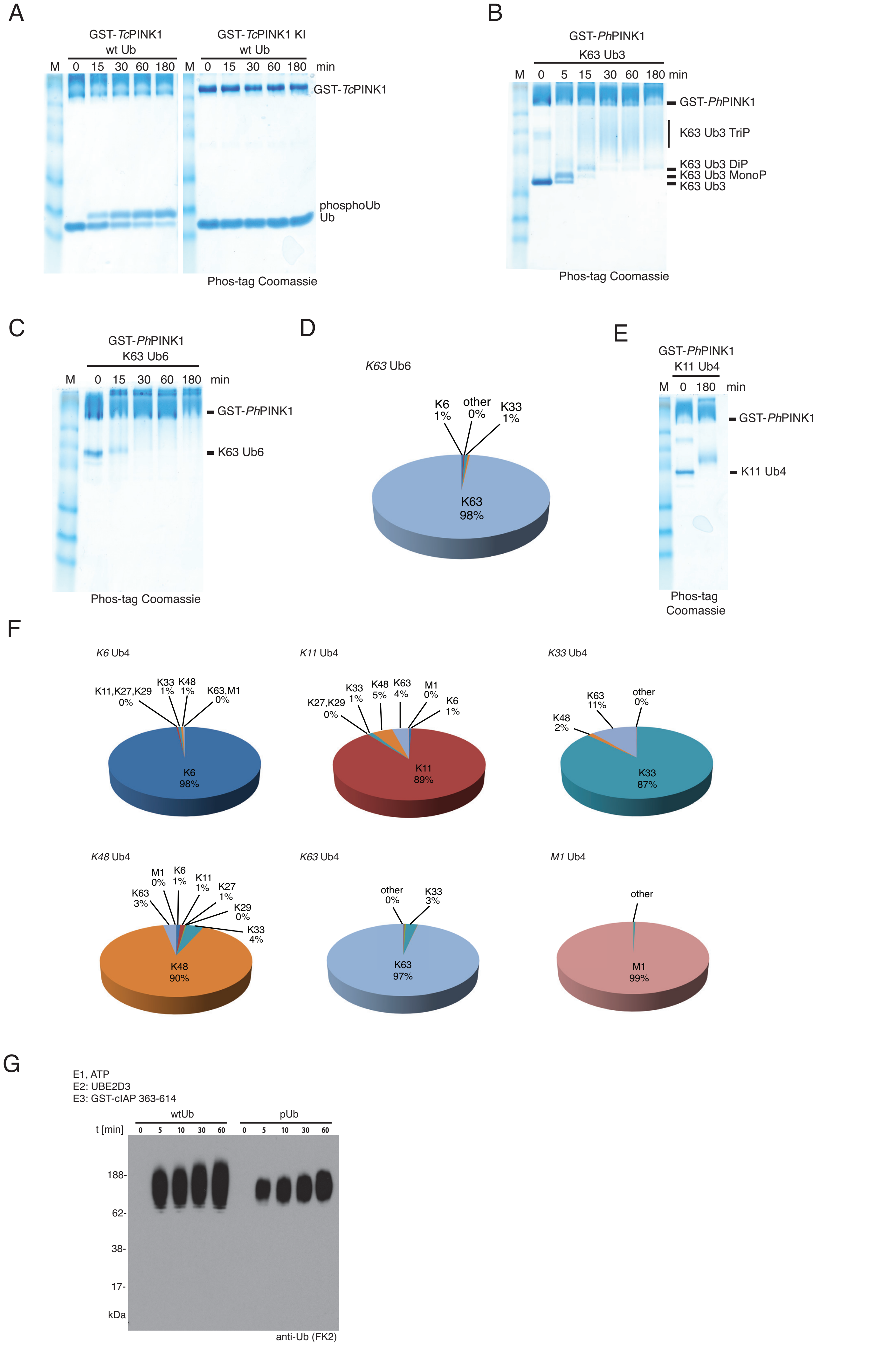

Supplementary Figure 1. Phosphorylation of Ub and polyUb.

A) Phos-tag gel as in **Figure 1B**, but in this case active (*left*) and kinase inactive D359A (*right*) forms of GST-tagged *Tc*PINK1 were used in a time course. **B)** Phosphorylation of Lys63-linked triUb with GST-tagged *Ph*PINK1, followed by Phos-tag gel in a time course. Distinct mono-phosphorylated (monoP) and di-phosphorylated (diP) forms are labeled while presumably tri-phosphorylated (triP) chains appear as a smear. **C)** Phosphorylation time course of Lys63-linked hexaUb (K63 Ub6) with GST-tagged *Ph*PINK1 on Phos-tag gel. **D)** Reaction from **C** analyzed by AQUA mass spectrometry as in **Figure 1D**. **E)** Phosphorylation of Lys11-linked tetraUb (K11 Ub4) on Phos-tag gel. **F)** AQUA mass spectrometry analysis on reactions from **Figure 1E**. **F)** Anti-Ub (FK2) antibody recognizes phosphoUb chains in a chain assembly reaction with cIAP as performed in **Supplementary Figure 11D**. See **Figure 6** below for more details.

Supplementary Figure 2

A

phosphoUb major species

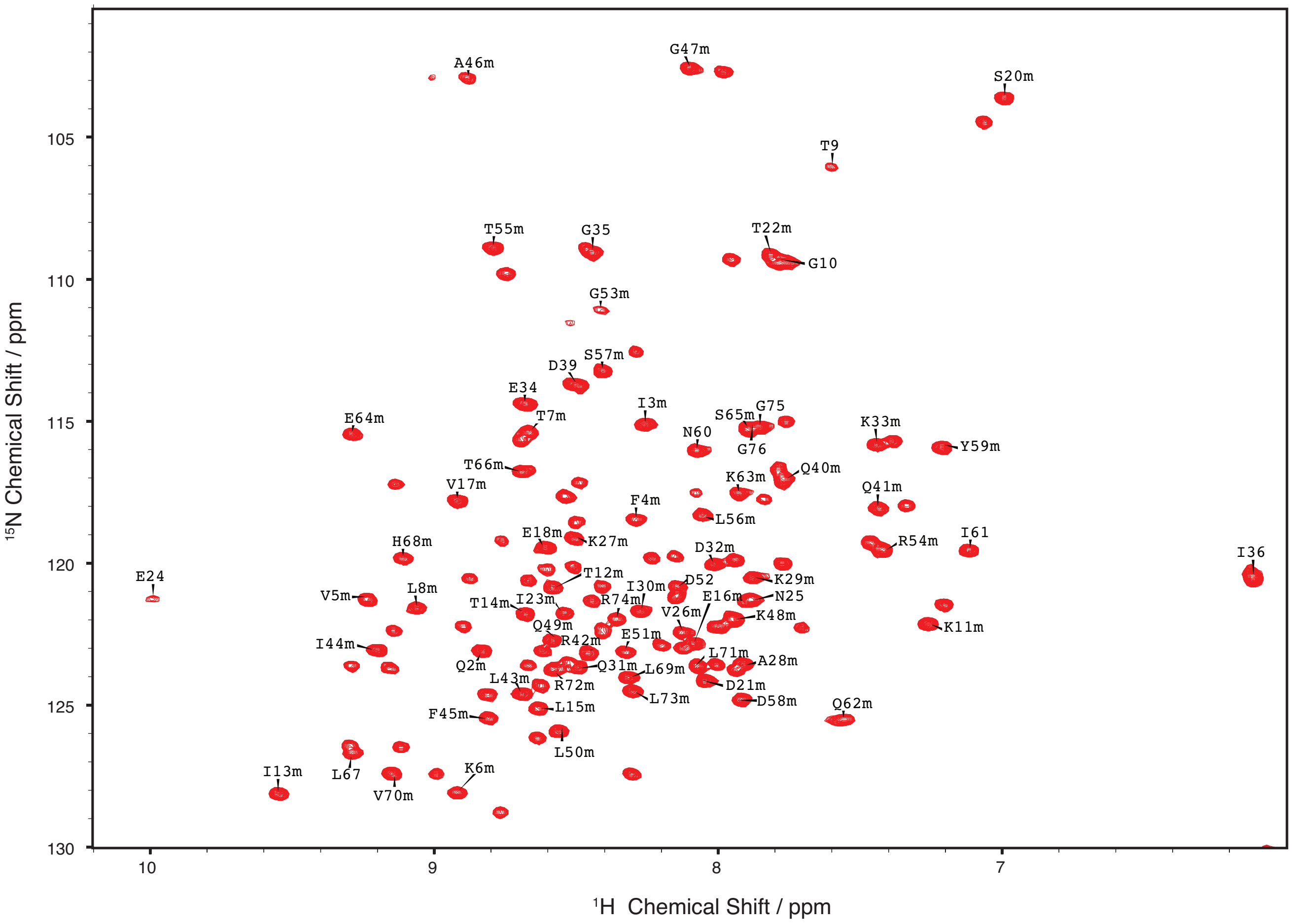

B

phosphoUb minor species

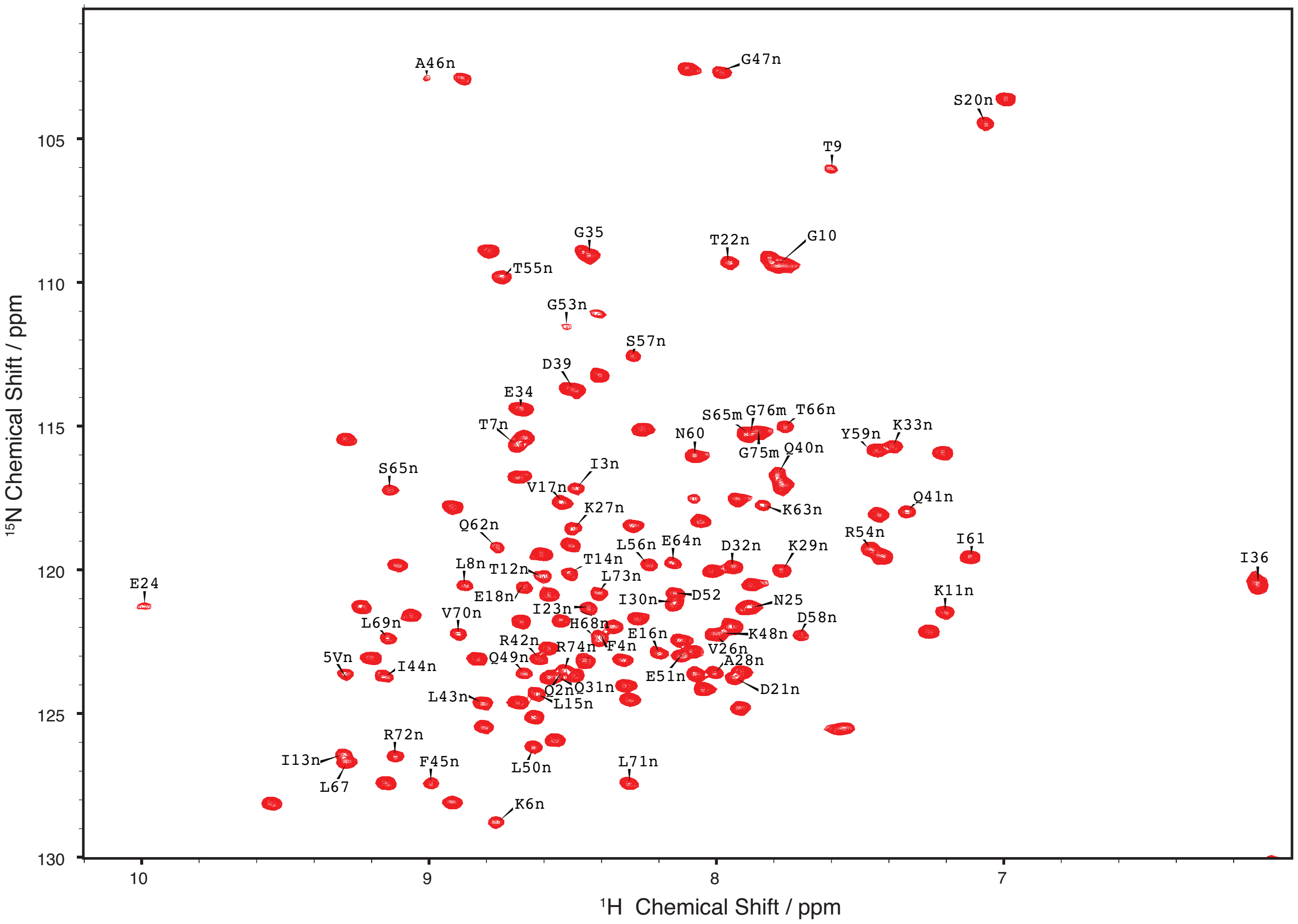

Supplementary Figure 2. Assignment of phosphoUb species.

Complete chemical shift assignment based on 3D triple resonance experiments with ^{13}C, ^{15}N-labeled phosphoUb. Chemical shift positions of the major (m) **(A)** and of the minor (n) **(B)** form of phosphoUb are labeled. Where the two forms share the same chemical shift only the residue type and number is given.

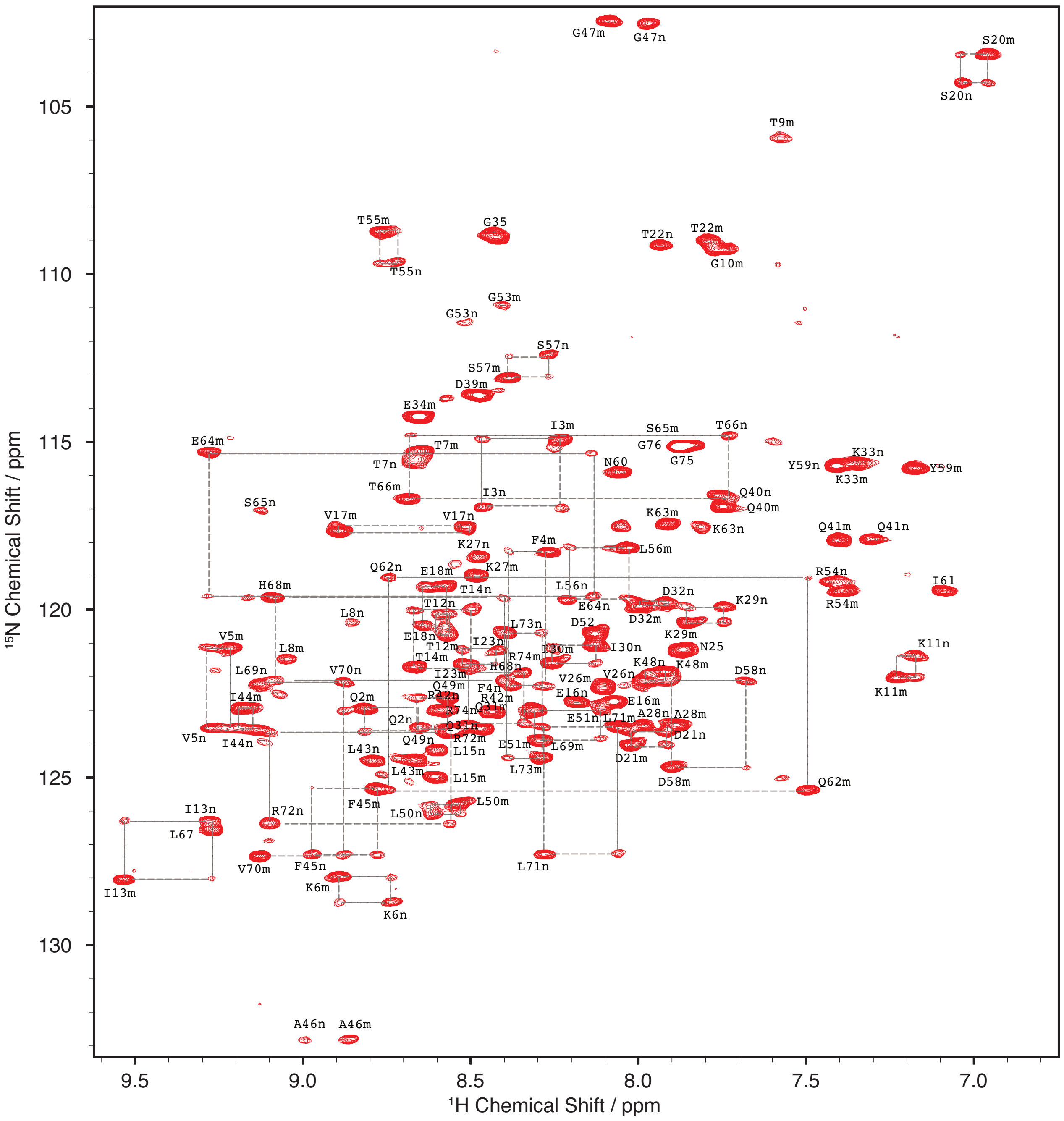

G47m
G47n
S20m
S20n
T9m
T55m
T55n
G35
T22n
T22m
G10m
G53m
G53n
S57n
S57m
D39m
E34m
I3m
S65m
T66n
E64m
T7m
G76
T7n
G75
N60
Y59n
K33n
K33m
Y59m
T66m
I3n
Q40n
Q40m
S65n
K63m
V17m
V17n
K63n
Q41m
Q41n
F4m
L56m
K27n
R54n
Q62n
E18m
K27m
I61
H68m
L56n
T14n
D32n
R54m
E64n
K29n
T12n
D32m
L8n
L73n
D52
E18n
K29m
I23n
V5m
T12m
I30n
N25
L8m
I30m
R74m
T14m
K48n
K48m
L69n
V70n
H68n
I23m
V26m
V26n
D58n
K11n
K11m
Q49m
F4n
E16n
R42n
R42m
I44m
Q2m
E16m
R74n
Q31m
A28n
A28m
Q2n
E51n
L71m
V5n
I44n
Q49n
R72m
Q31n
D21n
E51m
L43n
L15n
L69m
D21m
L43m
L73m
L15m
D58m
Q62m
F45m
L50m
I13n
R72n
L50n
L67
L71n
V70m
F45n
K6m
I13m
K6n
A46n
A46m
105
110
115
120
125
130
15N Chemical Shift / ppm
9.5
9.0
8.5
8.0
7.5
7.0
1H Chemical Shift / ppm

Supplementary Figure 3. ZZ exchange spectroscopy.

ZZ exchange experiment of phosphoUb with a mixing time of 92 ms illustrating the occurrence of exchange (cross) peaks between the (auto) resonances for the major and minor forms.

Supplementary Figure 4

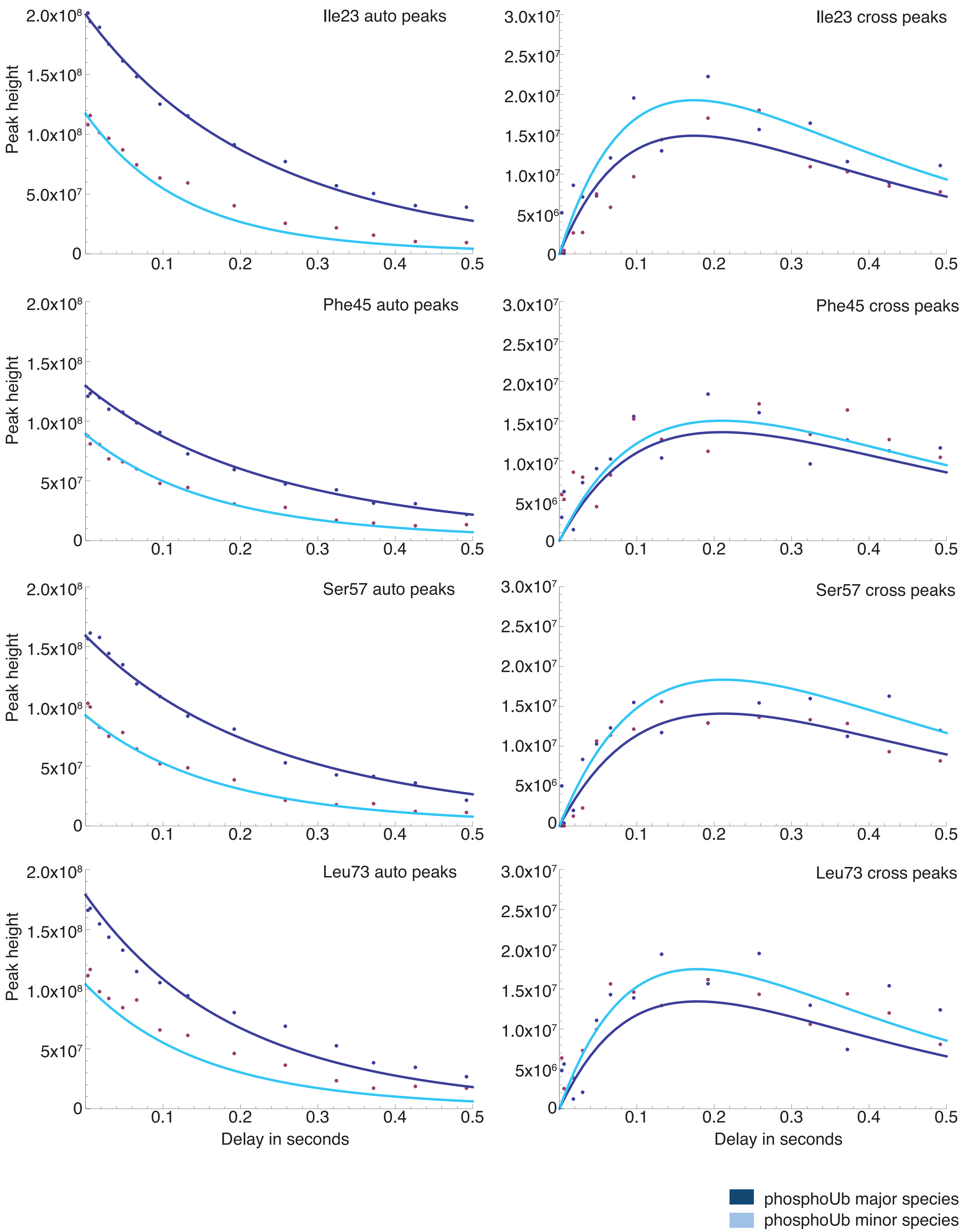

Supplementary Figure 4. ZZ exchange data.

Peak intensities of (auto) signals of the major form (blue) and minor form (lightblue) (*left column*) are fitted simultaneously to cross peaks as result of major to minor exchange (blue) and minor to major exchange (lightblue) (*right column*) for residues Ile23, Phe45, Ser57 and Leu73 using the methods described in (Latham *et al*, 2009). This compensates for the loss of signal intensity due to longitudinal T_1 relaxation (apparent from the decaying auto peaks). The exchange rate was calculated to be $1.76 \pm 0.09\ s^{-1}$.

Supplementary Figure 5

A

phosphoUb major species

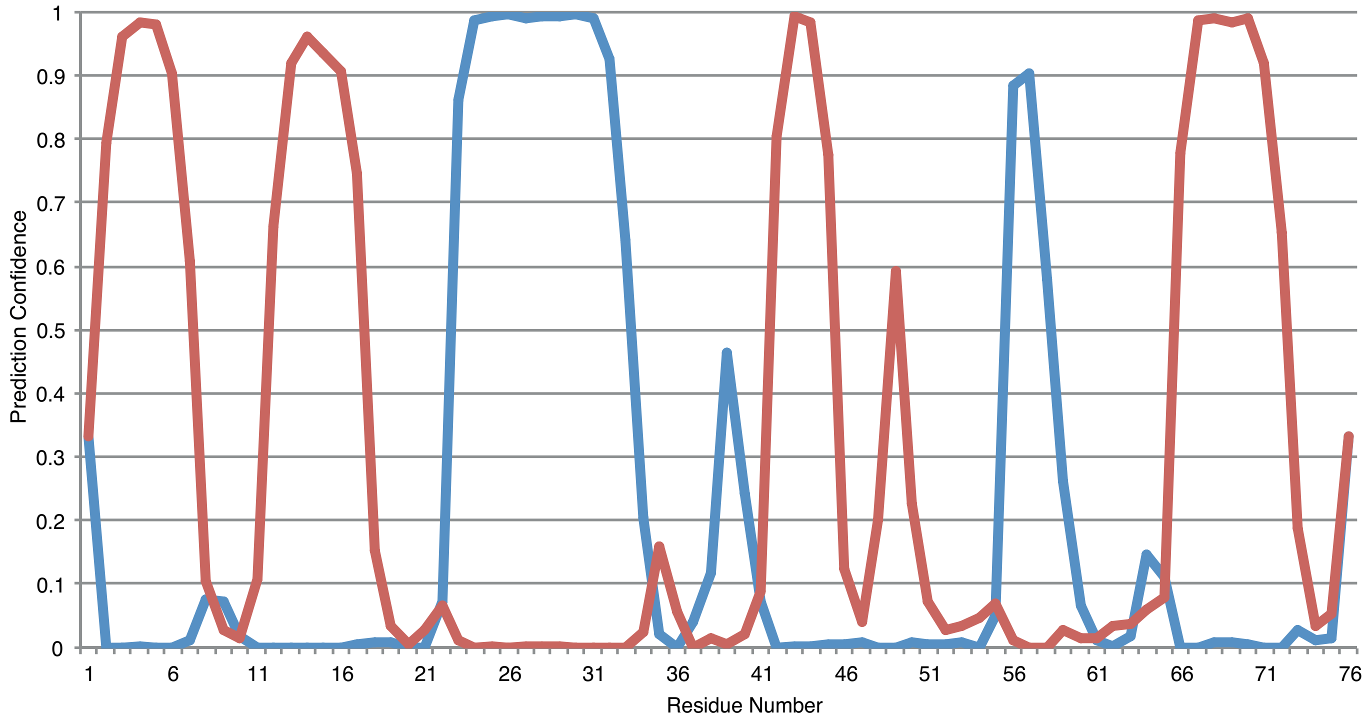

B

phosphoUb minor species

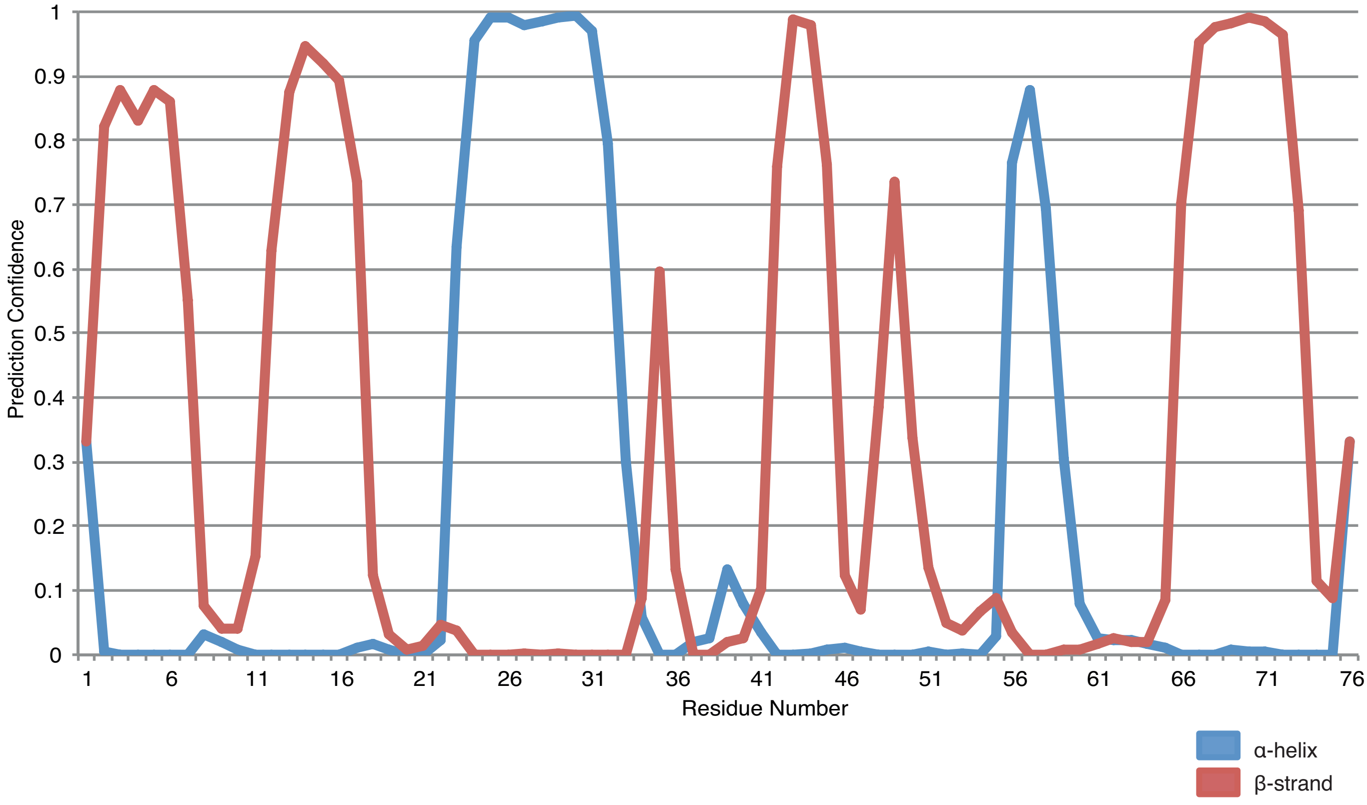

Supplementary Figure 5. PhosphoUb secondary structure prediction. Backbone chemical shifts (HN, N, CA, CB and HA) were submitted to TALOS+ (Shen *et al*, 2009) for secondary structure prediction. Confidence in the prediction of α-helix (blue) or β-sheet (red) is given for the major **(A)** and minor **(B)** phosphoUb species. See **Figure 2E** for annotation.

Supplementary Figure 6

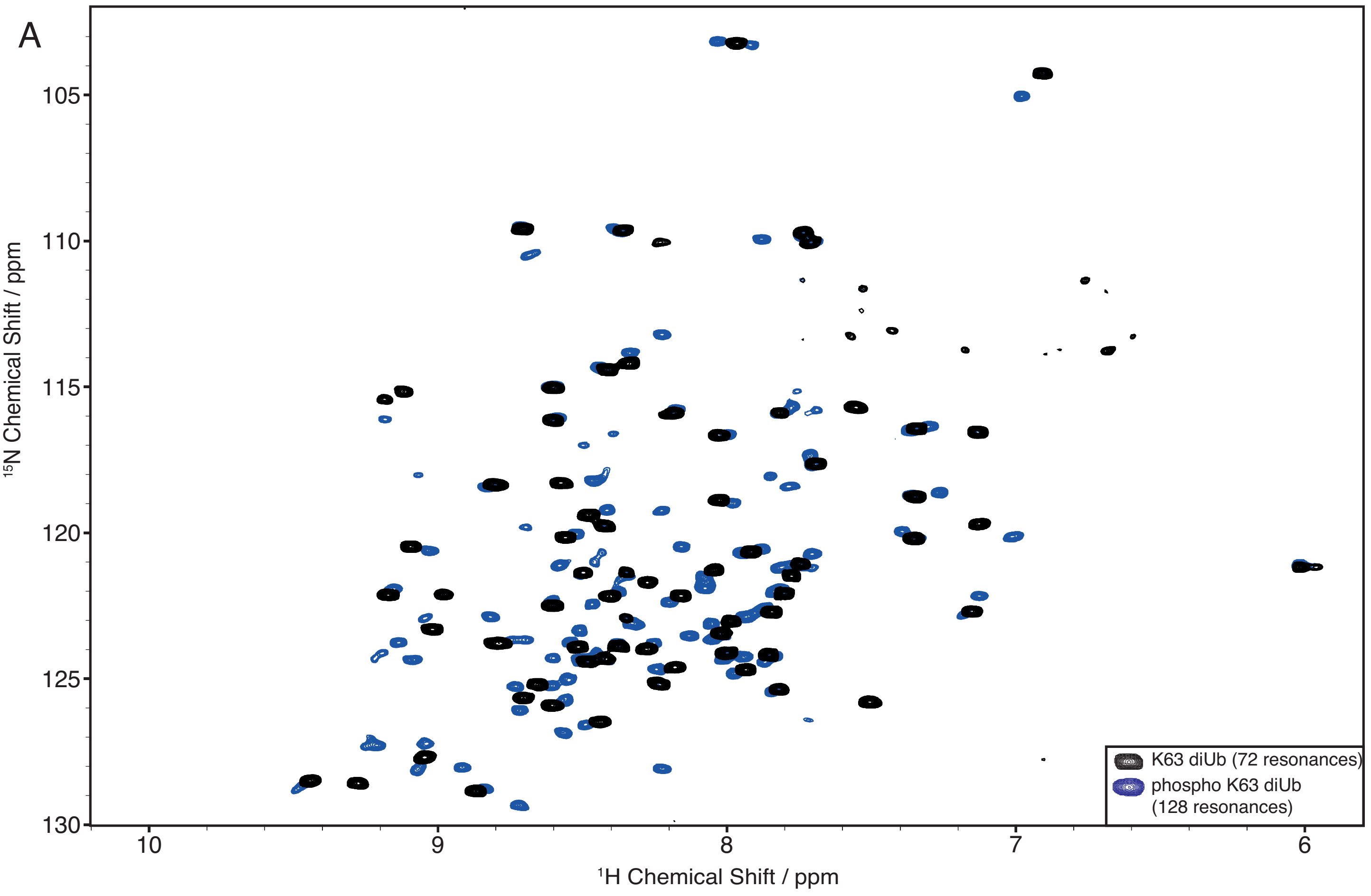

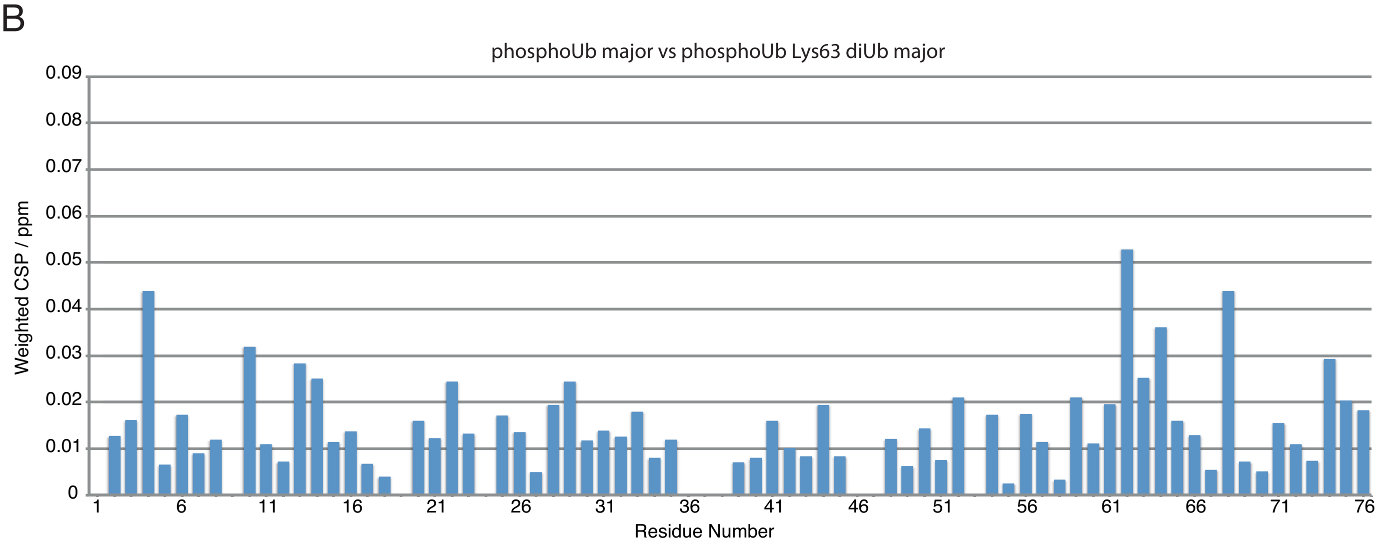

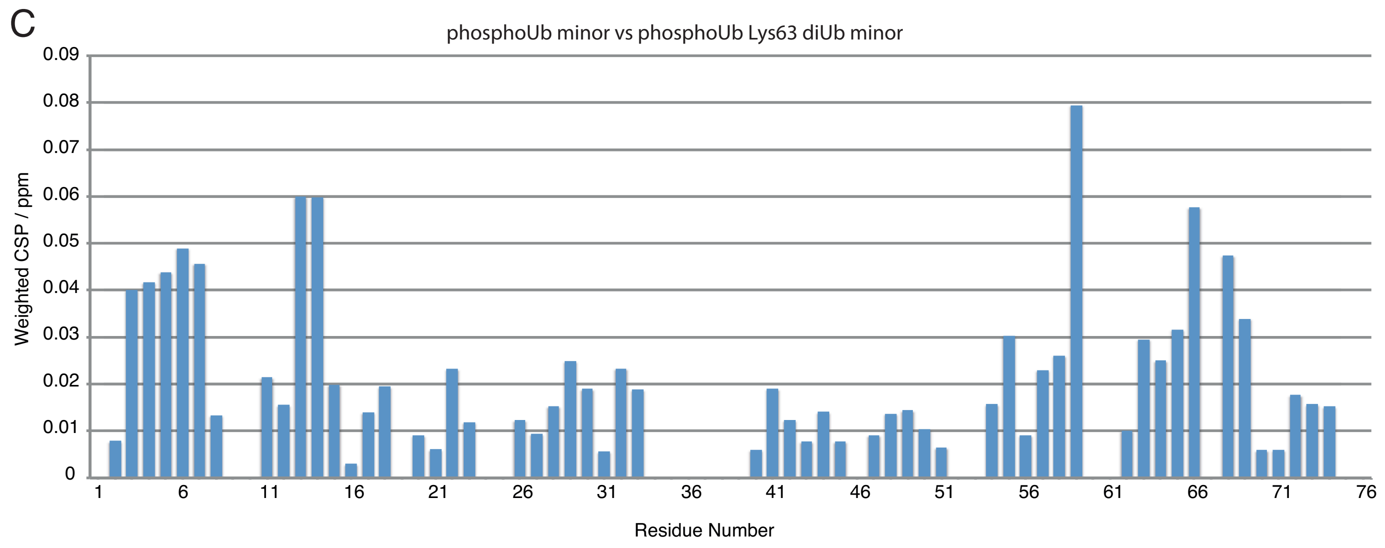

Supplementary Figure 6. Phosphorylation of Lys63 diUb.

A) A comparison of the BEST-TROSY spectrum of wild-type Lys63 diUb (black, 72 peaks) with phosphorylated wild-type Lys63 diUb (blue, 128 peaks). **B)** Weighted chemical shift perturbations of the major species of phosphoUb (**Figure 2A**) against the major species of phosphorylated wild-type Lys63 diUb. **C)** Weighted chemical shift perturbations of the minor species of phosphoUb (**Figure 2A**) against the minor species of phosphorylated wild-type Lys63 diUb.

Supplementary Figure 7

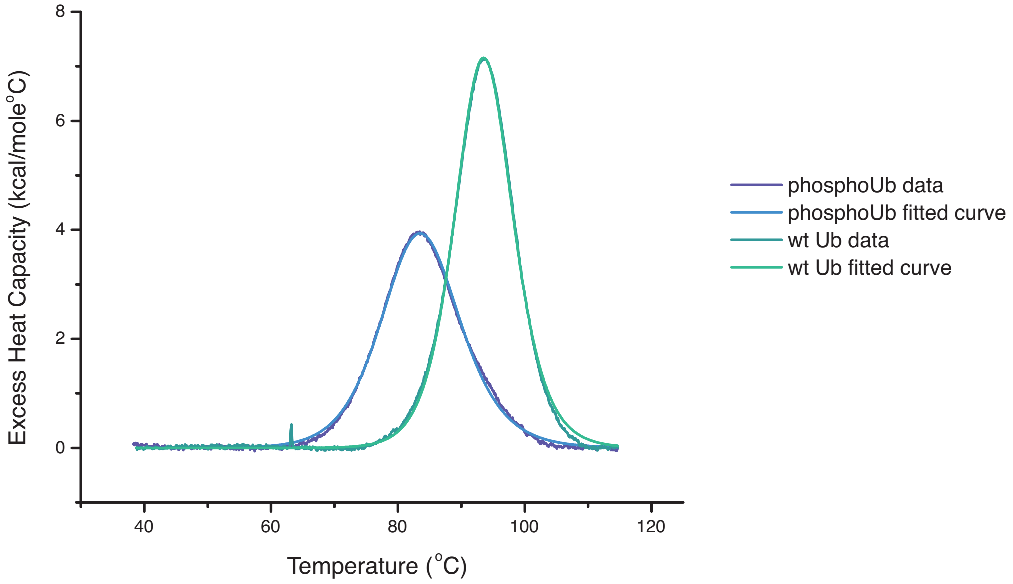

Supplementary Figure 7. Stability measurements of Ub and phosphoUb. DSC endotherms for Ub and phosphoUb (dark cyan / blue lines, respectively) with fits to the data (light cyan / blue lines) for Ub; T_m 93.6 °C, ΔH calorimetric 88 kcal/mol and ΔH van't Hoff 87 kcal/mol, and for phosphoUb T_m 83.7 °C, ΔH calorimetric 65 kcal/mol and ΔH van't Hoff 61 kcal/mol.

Supplementary Figure 8

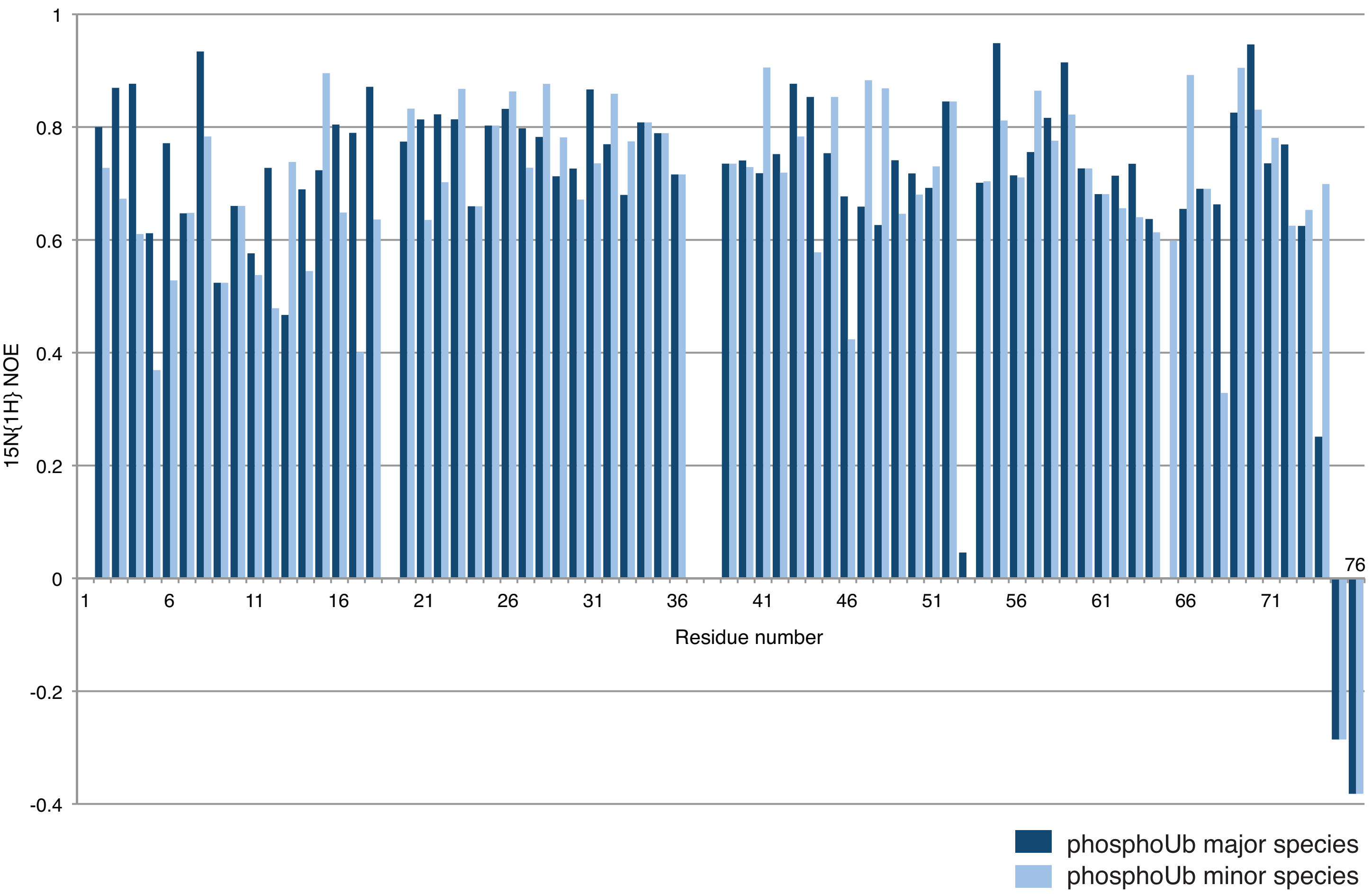

Supplementary Figure 8. HetNOE of phosphoUb.

$^{15}N\{^{1}H\}$hetNOE values for the major (dark blue) and minor (light blue) species of phosphoUb. The value for the major form of pSer65 is omitted as it is artificially suppressed in this experiment due to the proximity of its cross peak to the highly flexible and therefore negative peak of Gly76 (see **Supplementary figure 2A**).

Supplementary Figure 9

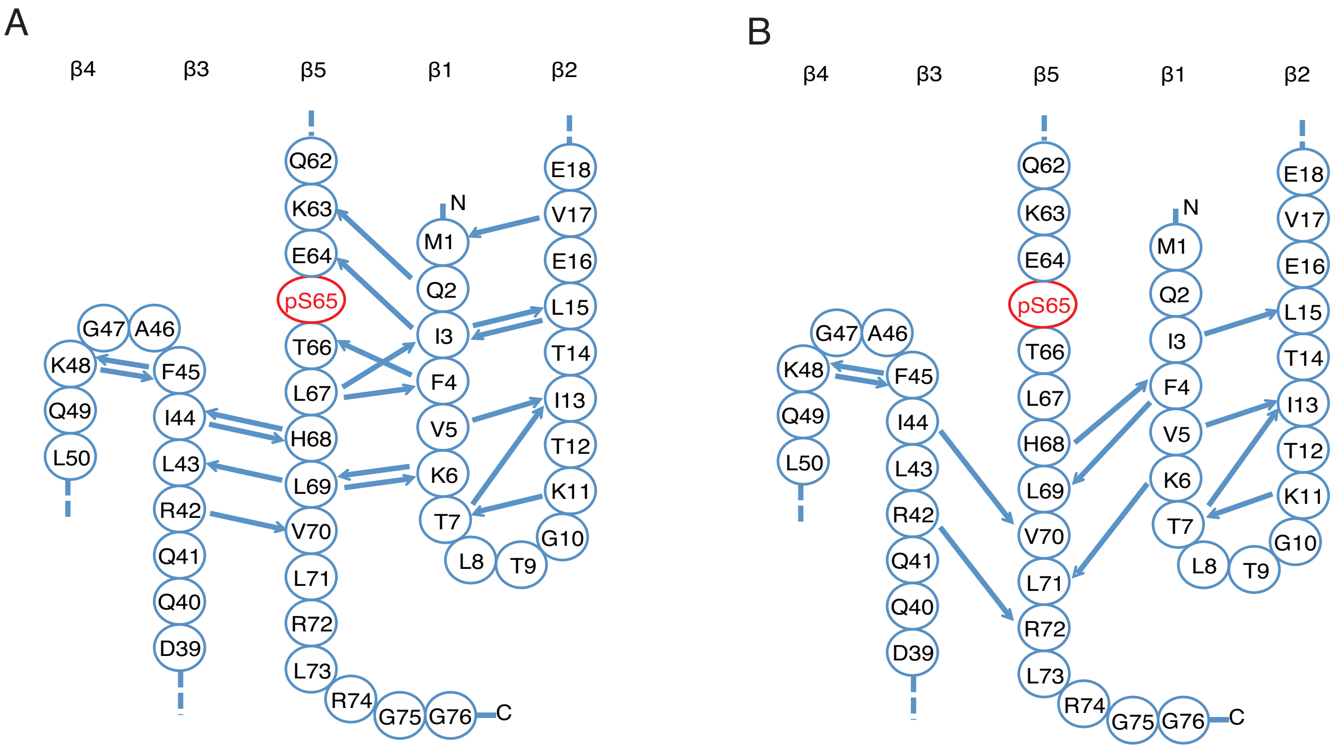

Supplementary Figure 9. Long distance NOE contacts of major and minor species of phosphoUb.

The long distance NOE contacts of **A)** the major species of phosphoUb and **B)** the minor species of phosphoUb. The direction of the arrow indicates the assignment of a NOE cross peak from the HN proton to either an HN, HA or HB for a given residue. As a rule of thumb, NOE cross peaks are observed for resonances up to 5 Å apart. The observed contacts confirm the altered hydrogen-bonding pattern seen for the minor species of phosphoUb (**Figure 4**).

Supplementary Figure 10

A

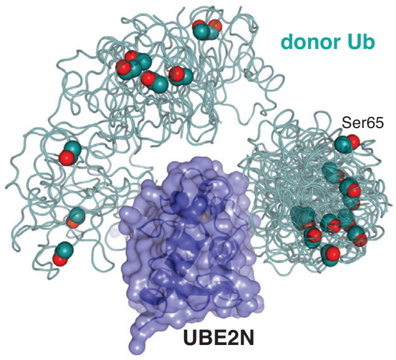

B

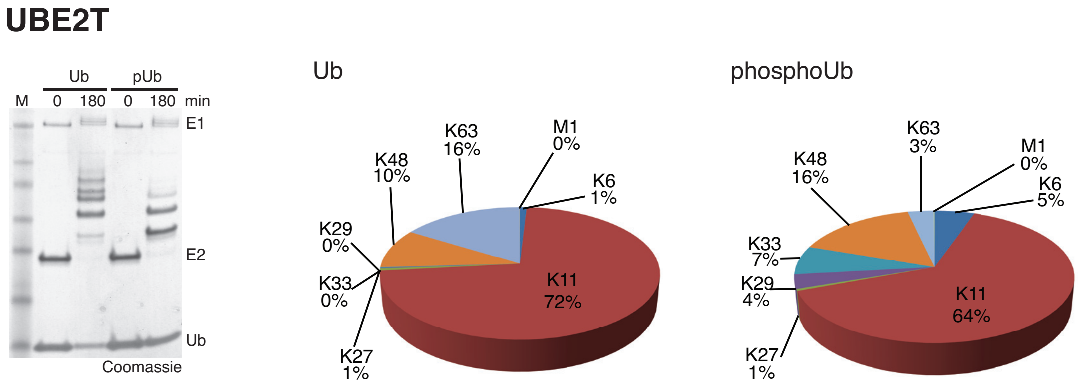

C

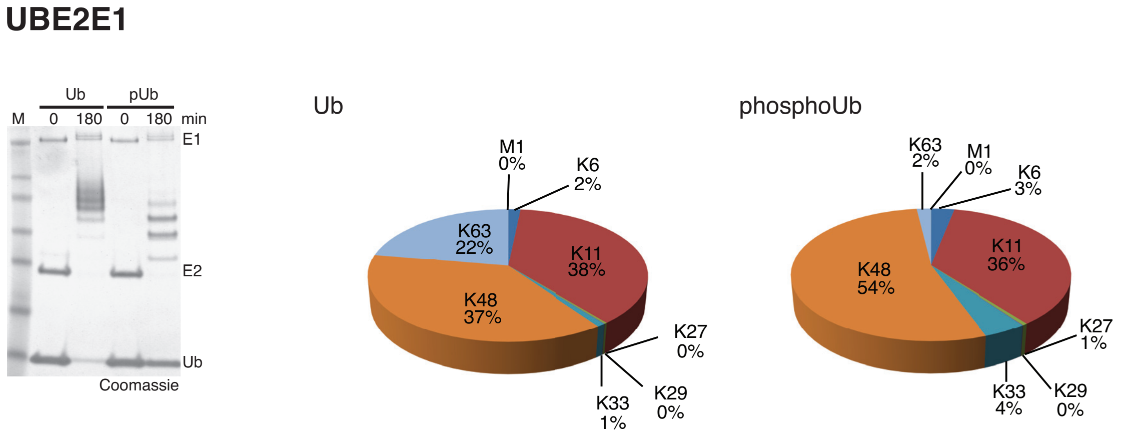

D

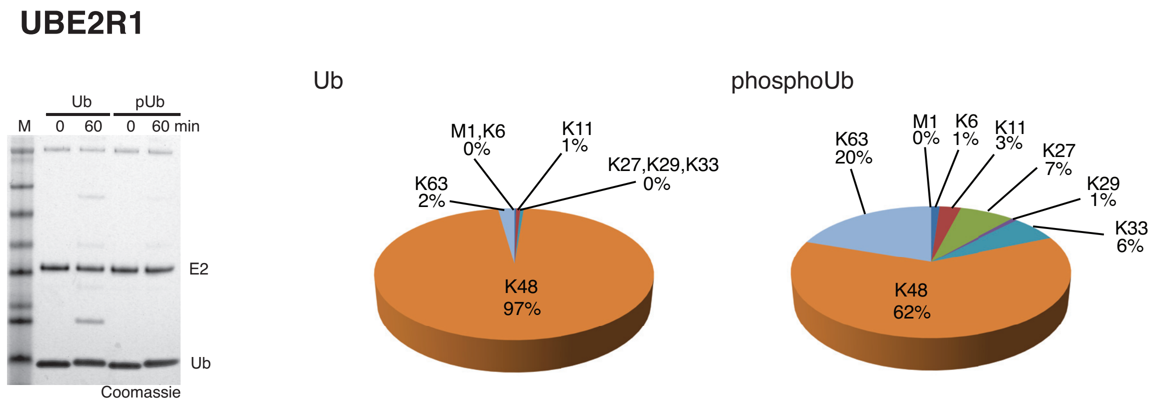

Supplementary Figure 10. Ubiquitin chain composition in E2 autoubiquitination reactions.

A) SAXS-derived ensemble of UBE2N, where the E2 is shown under a blue surface and the Ub is shown as a green ribbon. Ser65 is shown in sphere representation. Ser65 does not contact the E2 enzyme in any orientation of Ub. PDB files were downloaded from the Klevit-lab website (http://depts.washington.edu/klvtlab/). **B-D)** Coomassie gels of a reaction with UBE2T **(B)**, UBE2E1 **(C)** and UBE2R1 **(D)** comparing Ub and phosphoUb, and subsequent AQUA-based chain composition analysis. Quantification of linkages is shown in **Figure 5C**.

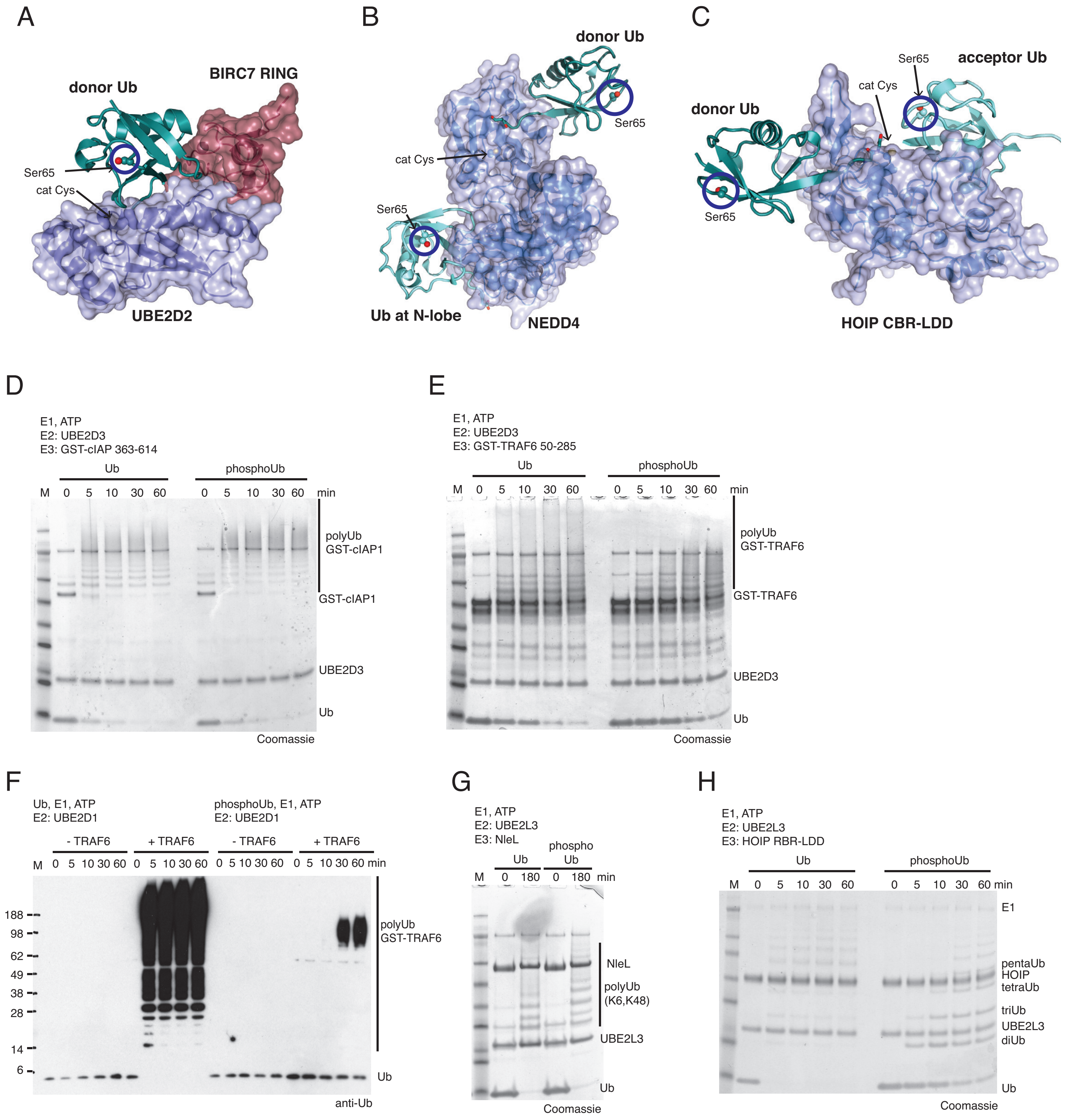
A
donor Ub
BIRC7 RING
Ser65
cat Cys
UBE2D2
B
donor Ub
Ser65
cat Cys
Ser65
Ub at N-lobe
NEDD4
C
Ser65
acceptor Ub
cat Cys
donor Ub
Ser65
HOIP CBR-LDD
D
E1, ATP
E2: UBE2D3
E3: GST-cIAP 363-614
Ub
phosphoUb
M 0 5 10 30 60 0 5 10 30 60 min
polyUb
GST-cIAP1
GST-cIAP1
UBE2D3
Ub
Coomassie
E
E1, ATP
E2: UBE2D3
E3: GST-TRAF6 50-285
Ub
phosphoUb
M 0 5 10 30 60 0 5 10 30 60 min
polyUb
GST-TRAF6
GST-TRAF6
UBE2D3
Ub
Coomassie
F
Ub, E1, ATP
E2: UBE2D1
phosphoUb, E1, ATP
E2: UBE2D1
- TRAF6
+ TRAF6
- TRAF6
+ TRAF6
M 0 5 10 30 60 0 5 10 30 60 0 5 10 30 60 0 5 10 30 60 min
188
98
62
49
38
28
14
6
polyUb
GST-TRAF6
Ub
anti-Ub
G
E1, ATP
E2: UBE2L3
E3: NleL
Ub
phospho Ub
M 0 180 0 180 min
NleL
polyUb
(K6,K48)
UBE2L3
Ub
Coomassie
H
E1, ATP
E2: UBE2L3
E3: HOIP RBR-LDD
Ub
phosphoUb
M 0 5 10 30 60 0 5 10 30 60 min
E1
pentaUb
HOIP
tetraUb
triUb
UBE2L3
diUb
Ub
Coomassie

Supplementary Figure 11. E3 ligase mediated assembly of phosphoUb chains

A) Structure of BIRC7 (red) bound to Ub~UBE2D2 (cyan and blue) (pdb-id 4auq, (Dou *et al*, 2012)). Ser65 is not contacted by the E2 or E3 enzyme. **B)** Composite model that combines the crystal structure of NEDD4 bound to Ub~UBE2D (pdb-id 3jw0, (Kamadurai *et al*, 2009), UBE2D is omitted for clarity) and the crystal structure of NEDD4L with Ub bound to HECT N-lobe (2xbb, (Maspero *et al*, 2011)). In either position of Ub, Ser65 is not contacting the E3 ligase. **C)** Structure of the catalytic core of HOIP, comprising the catalytic IBR (CBR) domain (also known as RING2) and the C-terminal LDD domain (4ljo, (Stieglitz *et al*, 2013)), shown with acceptor and donor Ub (cyan) bound. Ser65 does not contact the CBR-LDD in either Ub. **D)** Ubiquitination reaction as in **Figure 6A** with GST-cIAP1 and UBE2D3. **E)** Ubiquitination reaction with UBE2D3 and GST-TRAF6 50-285. **F)** Ubiquitination reaction as in **E** with or without TRAF6, demonstrating that chain formation is TRAF6-dependent also with phosphoUb. **G)** Ubiquitination reaction with NleL and UBE2L3. This combination makes free Lys6/Lys48-linked polyUb (Hospenthal *et al*, 2013). A longer time point as compared to **Figure 6J** is shown. **H)** Longer time course of the reaction of HOIP RBR-LDD / UBE2L3 as shown in **Figure 6K**, showing that HOIP generates short linear chains at later time points.

Supplementary Figure 12

A

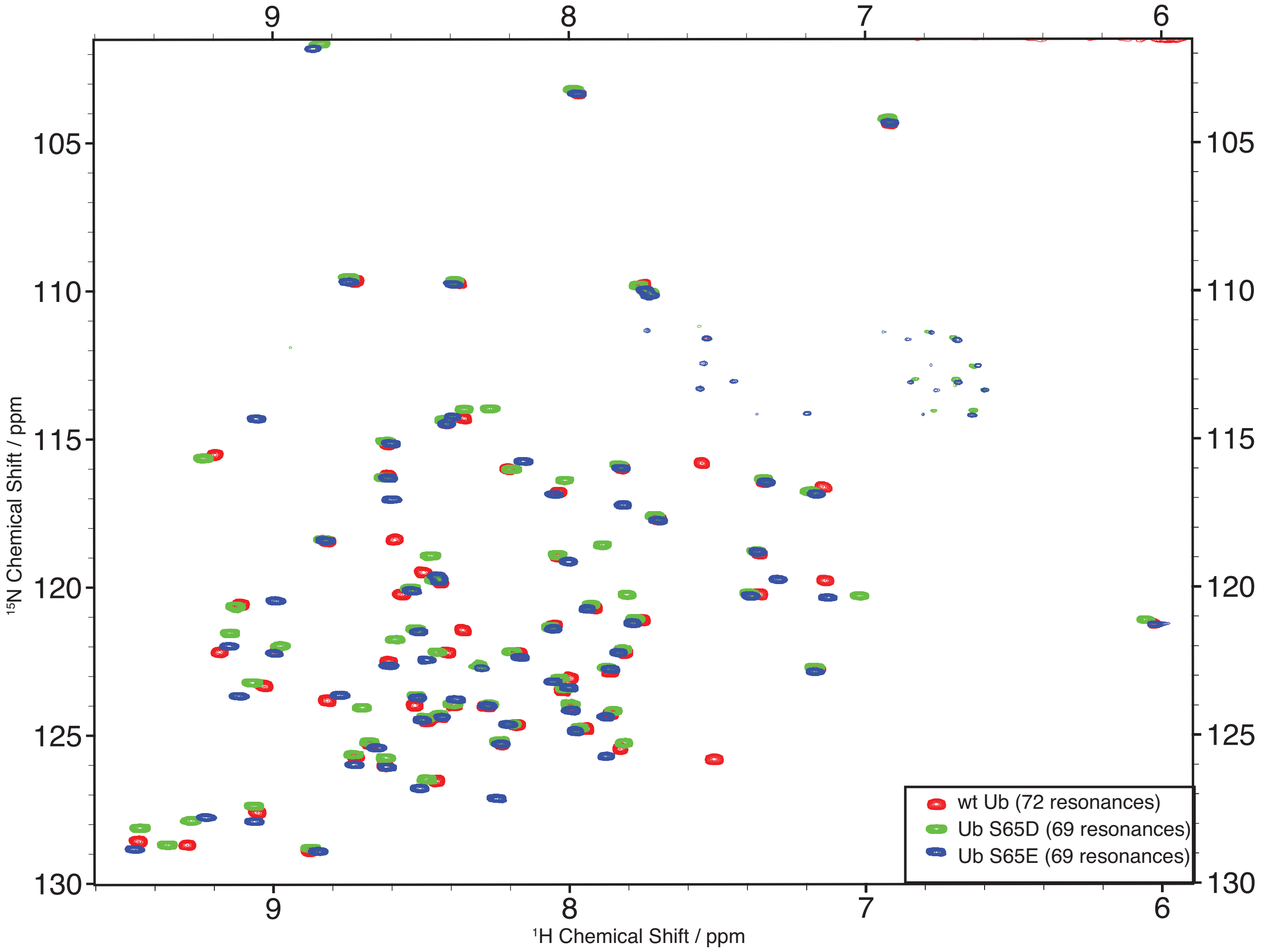

B

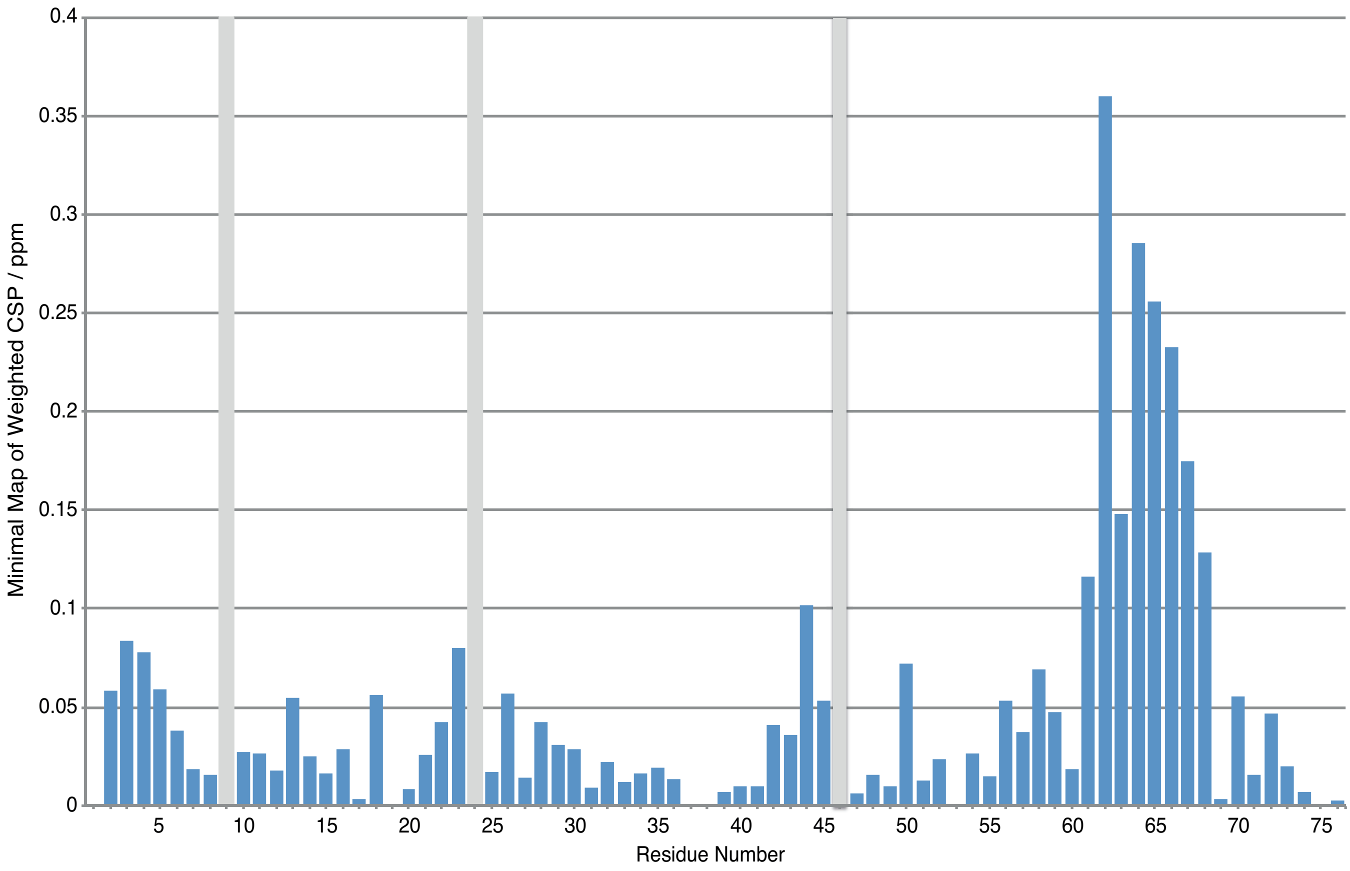

Supplementary Figure 12. Comparison of Ub S65E with wild-type Ub.

A) The BEST-TROSY spectrum of wild-type Ub (red) overlaid with the BEST-TROSY spectrum of Ub S65E (blue) and Ub S65D (green). The phosphomimetic Ub mutants shows no signs of the $Ub^{retraCT}$ conformation as observed for phosphoUb. **B)** A minimal map of the weighted chemical shift perturbations (CSPs) between the peaks of S65E Ub and wild-type Ub. Resonances for Thr9, Glu24 and Ala46 are presumed to be missing due to line broadening.

Supplementary Figure 13

A

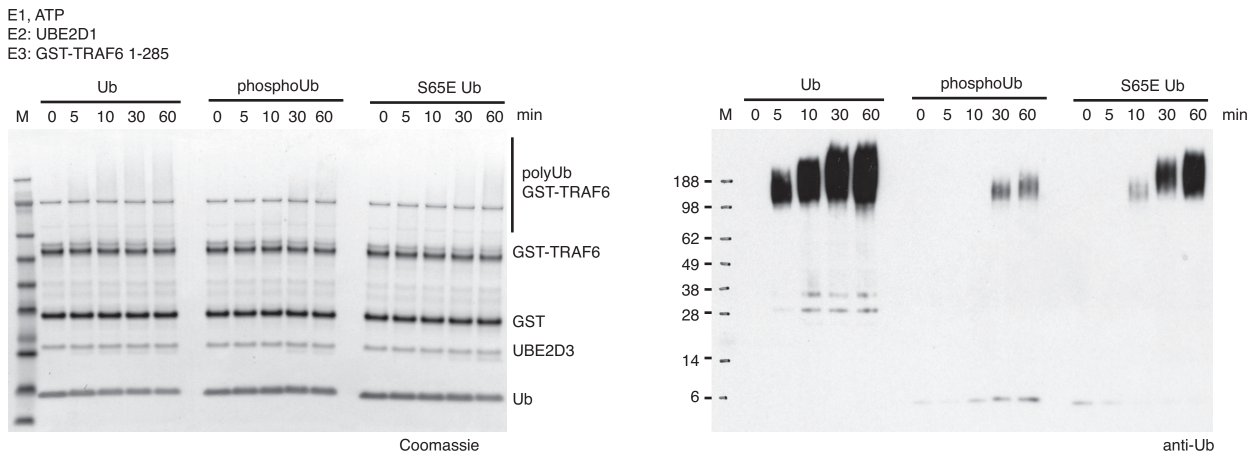

B

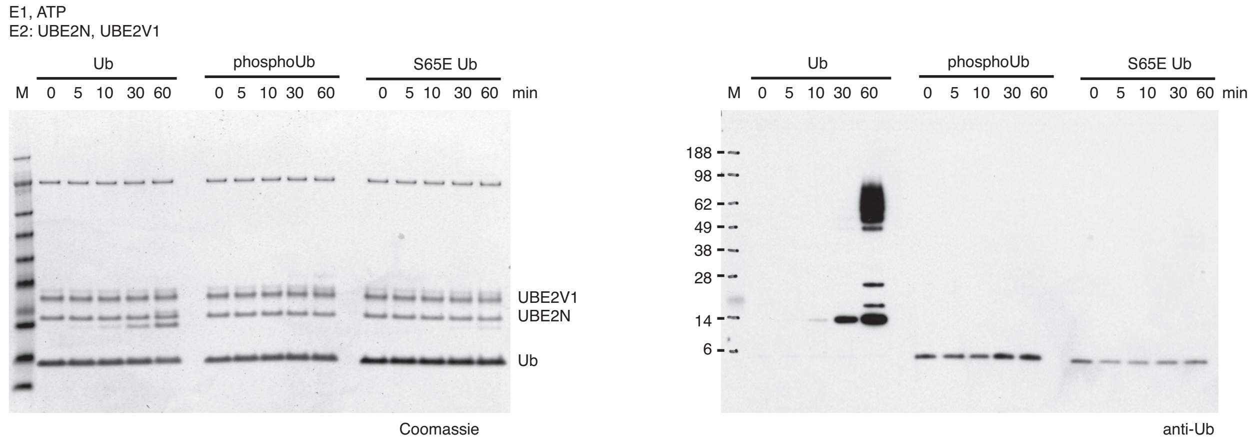

Supplementary Figure 13. Comparison of Ub, phosphoUb , and Ub S65E in chain assembly.

A) Time course for a ligase reaction using GST-TRAF6 (1-285) and UBE2D1 combined with Ub, phosphoUb, or S65E Ub. Proteins were detected with Coomassie and anti-Ub Westerns. **B)** Reactions with UBE2N/UBE2V1 combined with Ub, phosphoUb, or S65E Ub. Proteins were detected as in **A**.

Supplementary Figure 14

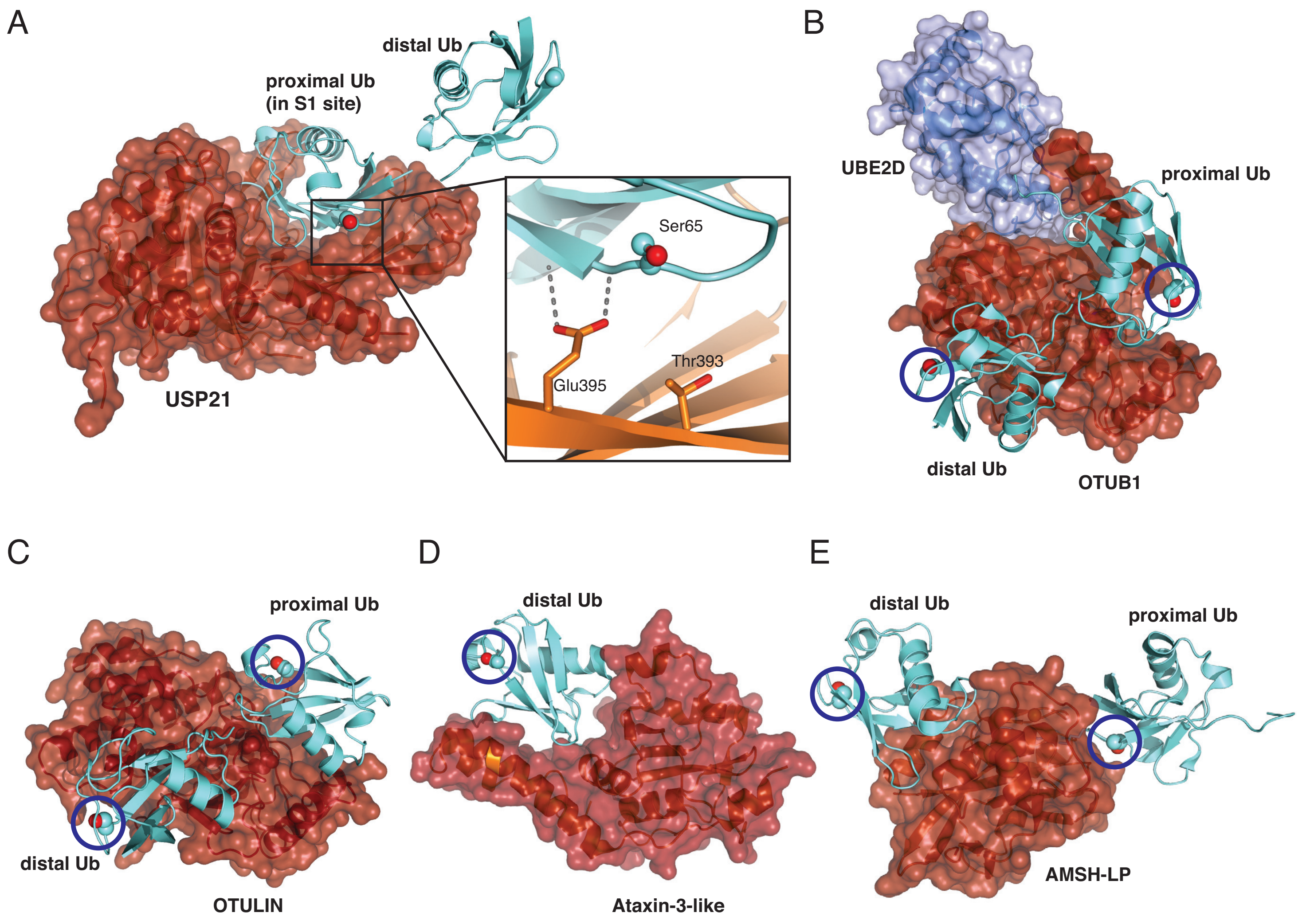

Supplementary Figure 14. PhosphoUb chain disassembly by deubiquitinases.

Structures of DUBs (red under a semi-transparent surface) bound to one or where available two Ub molecules (cyan). Ub Ser65 atoms are shown as spheres and indicated with a blue circle. **A)** Structure of USP21 bound to linear diUb-aldehyde (2yb5, (Ye *et al*, 2011)). The insert shows the distal binding site with USP21 Glu435 contacting the backbone of Ub. **B)** Structure of OTUB1 bound to E2 (blue, here UBE2D) with distal and proximal Ub bound (pdb-id 4ddg, (Juang *et al*, 2012; Wiener *et al*, 2012)). **C)** Structure of inactive OTULIN bound to Met1-linked diUb (3znz, (Keusekotten *et al*, 2013)). **D)** Crystal structure of the MJD-family protein Ataxin-3L with Ub bound in the distal site (3o65, (Weeks *et al*, 2011)). **E)** Crystal structure of the JAMM family protein AMSH-LP with Lys63-linked diUb bound across the active site (2znv, (Sato *et al*, 2008)).

Table S1. Peptides used for parallel reaction monitoring quantitation. Internal standards were isotopically labelled (^{13}C, ^{15}N) and the corresponding residue(s) is underlined. Modified residues with ubiquitination and phosphorylation are indicated by (GG) or p, respectively. Fragment ions used for quantitation are listed.

Name	Peptide Sequence	Light (m/z)	Heavy (m/z)	Fragment Ions
K6	MQIFVK(GG)TLTGK	465.9270+++	468.2661+++	y^5, y^4, y^3, y^2
K11	TLTGK(GG)TITLEVEPSDTIENVK	801.4269+++	803.4315+++	$y^{11}, y^{10}, y^9, y^8, y^7, y^6$
K27	TITLEVEPSDTIENVK(GG)AK	701.0390+++	703.0436+++	$y^{11}, y^7, y^6, y^5, y^3$
K29	AK(GG)IQDK	408.7323++	411.7361++	y^5, y^4, y^3, y^2
K33	IQDK(GG)EGIPPDQQR	546.6129+++	548.6175+++	y^6, y^5, y^3
K48	LIFAGK(GG)QLEDGR	487.6001+++	489.9391+++	y^6, y^5, y^4, y^3, y^2
K63 (+3)	TLSDYNIQK(GG)ESTLHLVLR	748.7376+++	751.0767+++	$y^{10}, y^9, y^8, y^5, y^4, y^3$
K63 (+4)		561.8050++++	563.5593++++	
M1	GGMQIFVK	448.2389++	451.2458++	y^6, y^5, y^4, y^3, y^2
TLS	TLSDYNIQK	541.2798++	544.7884++	y^7, y^5, y^4, y^3, y^2
EST	ESTLHLVLR	356.5451+++	358.8842+++	y^5, y^4, y^3, y^2
TITLE	TITLEVEPSDTIENVK	894.4673++	897.4742++	$y^{12}, y^{11}, y^{10}, y^9, y^4$
TL-EpST (+3)	TLSDYNIQKEpSTLHLVLR	737.3787+++	739.7178+++	$y^{10}, y^9, y^8, y^5, y^4, y^3$
TL-EpST (+4)		553.2859++++	555.0402++++	
K63pS65 (+3)	TLSDYNIQK(GG)EpSTLHLVLR	775.3931+++	777.7321+++	$y^{10}, y^9, y^8, y^5, y^4, y^3$
K63pS65 (+4)		581.7966++++	583.5509++++	
EpST	EpSTLHLVLR	383.2006+++	385.5396+++	y^5, y^4, y^3, y^2

SUPPLEMENTARY MATERIAL AND METHODS

PINK1 purification

Constructs of GST-tagged *Ph*PINK1 (aa 115-575) and *Tc*PINK1 (aa 128-570) codon-optimized for bacterial expression were expressed in Rosetta2 pLacI cells by inducing with 150 µM IPTG (OD_{600} ~1.0) at 37°C. The cells were grown at 18°C for 12 h and lysed by sonication in 270 mM sucrose, 10 mM glycerol 2-phosphate disodium, 50 mM sodium fluoride, 14 mM β-mercaptoethanol, 50 mM Tris (pH 8.0) with added DNase, Lysozyme and EDTA-free protease inhibitor tablets (Roche). After centrifugation (45000 x g, 30 min, 4°C) the supernatant was applied to Glutathione Sepharose 4B beads (GE Healthcare), agitated for 1 h at 4°C and subsequently washed in high salt buffer (500 mM NaCl, 10 mM DTT, 25 mM Tris pH 8.5) and equilibrated in low salt buffer (200 mM NaCl, 10 mM DTT, 25 mM Tris pH 8.5). The GST-tagged protein was eluted in low salt buffer containing 40 mM glutathione and applied to gel filtration (Superdex 200, GE Life Sciences) in low salt buffer. Untagged protein was cleaved for 12 h at 4°C with GST-3C protease and further purified by gel filtration (Superdex 75, GE Life Sciences) in low salt buffer. Protein-containing fractions were pooled, concentrated using a 30-kDa MWCO spin concentrator (Sartorius) and flash frozen in liquid nitrogen.

Generation of phosphoUb

10 mg of bovine Ub (Sigma-Aldrich, U6253) were incubated with glutathione S-transferase (GST)-tagged *Ph*PINK1 (aa 115-575) in reaction buffer (40 mM Tris pH 7.5, 10 mM $MgCl_2$, 0.6 mM DTT) for 3 h at room temperature. Reaction progress was monitored by ESI-MS. Reactions were stopped with apyrase, PINK1 was removed using glutathione sepharose 4B resin (GE Healthcare), and the reaction mixture was buffer exchanged to 20 mM Tris, pH 8.7. PhosphoUb was purified by anion exchange using a pH gradient from 20 mM Tris pH 8.7 to 50 mM Tris pH 7.4. Phosphorylated but not unphosphorylated Ub binds to MonoQ anion exchange resin at pH 8.7 and elutes at lower pH. Fractions containing phosphoUb were concentrated using

VivaSpin 3.5K concentrators and frozen. For NMR analysis, samples were generated as described below.

Phosphorylation analysis of Ub

Phosphorylation assays were performed by mixing 5 μM GST-PINK1 (species as indicated), 0.2 mg/ml Ub or Ub chains as indicated, 10 mM ATP in reaction buffer (40 mM Tris pH 7.5, 10 mM $MgCl_2$, 0.6 mM DTT) and incubated at room temperature for the specified time. The reaction was quenched in 4x LDS sample buffer and samples were applied on a 12% polyacrylamide gel containing 50 μM Phos-tag acrylamide (Wako Chemicals) and 0.77 mM $ZnCl_2$. MS analysis was performed as described below.

Intact protein MS analysis

Ub and phosphoUb protein stocks were diluted to 1 μM (50% ACN, 0.1% FA) prior to MS analysis. Samples were directly injected into the Q-Exactive (Thermo Fisher Scientific) mass spectrometer at a flow rate of 10 μl min^{-1}. Protein ionization was achieved using Heated Electrospray Ionization (HESI-II probe, Thermo Fisher Scientific). Ionization settings included the following: spray voltage, 6.0 kV; capillary temperature, 320°C; sheath gas, 10; S-lens RF level, 50. Raw spectra were deconvoluted using the Xtract node of Thermo Xcalibur Qual Browser version 2.2. Extract node settings included the following: generated mass mode, MH+; resolution, 140,000; S/N threshold, 3; mass range, 800-2000 *m/z*; fit factor, 44%; remainder, 25%.

Parkin activity assays

For Parkin activation assays a mixture of 0.1 μM E1, 9 μM Parkin, 10 mM ATP and ligation buffer (40 mM Tris pH 7.5, 10 mM magnesium chloride, 0.6 mM dithiothreitol) was pre-incubated for 1 h at 30°C. The total Ub concentration was kept at 0.5 mg/ml with the indicated mass percentages of phosphoUb supplemented. The ubiquitination reaction was started by adding 0.1 μM E1 and 3 μM UBE2L3 and samples were taken at the indicated time points by quenching 5 μl of the reaction with 5 μl LDS loading buffer (Invitrogen). Disulfide bridges were reduced by adding 2 μl of 200 mM DTT and prevented from reforming by acetylation with 1 μl of 0.5 M iodoacetamide.

Proteins were separated on NuPAGE 4-12% gradient Bis-Tris gels and Western blotting was performed by transfer on a nitrocellulose membrane and detection using a monoclonal anti-Ub FK2 antibody (Millipore).

PhosphoUb preparation for NMR analysis

Isotope labeled Ub was expressed and purified as described previously (Hospenthal *et al*, 2013). The phosphorylation of double labeled ^{15}N, ^{13}C-Ub was monitored in the spectrometer by ^{1}H,^{15}N 2D BEST-TROSY at room temperature containing 100 µM ^{15}N, ^{13}C-Ub, 2.5 µM *Ph*PINK1, 10 mM ATP, 25 µl 20 x reaction buffer (400 mM Tris pH 7.5, 100 mM $MgCl_2$, 6 mM DTT) which was adjusted to 500 µl with NMR buffer (18 mM Na_2HPO_4, 7 mM NaH_2PO_4 pH 7.2, 150 mM NaCl). 25 µl D_2O was added as an internal reference. After disappearance of the wt Ser65 signal of ^{15}N, ^{13}C-Ub, 21 µl of a 500 mM EDTA solution was added to quench the reaction.

For the long range HNCO and ^{15}N-edited NOESY experiments, 1.7 mM of ^{15}N, ^{13}C-Ub was phosphorylated with 17 µM GST-PhPINK1 in a 6 h reaction containing 10 mM ATP, 10 mM Tris pH 7.5, 10 mM $MgCl_2$, 0.6 mM DTT. After 6 h at room temperature the sample was applied to 600 µl equilibrated Glutathione Sepharose 4B beads (GE Healthcare). After 1 h agitation the beads were removed by gravity flow filtration and the flow through was applied to gel filtration (Superdex 75, GE Life Sciences) equilibrated in NMR buffer. Protein containing fractions were pooled and concentrated to 1 mM in a 3-kDa MWCO spin concentrator (Sartorius).

For Lys63 ^{15}N, ^{13}C-diUb generation the ^{15}N, ^{13}C-Ub was purified on an gel filtration column (Superdex 75, GE Life Sciences) and Lys63 chain assembly initiated in a volume of 1 ml by incubating 1.4 mM ^{15}N, ^{13}C-Ub with 2 µM E1, 16 µM UBE2N and 16 µM UBE2V1 in the presence of 10 mM ATP and ligation buffer (40 mM Tris pH 7.5, 10 mM magnesium chloride, 0.6 mM dithiothreitol). After 3 h at 37 °C the reaction was diluted with 50 mM sodium acetate pH 4.5, applied on a MonoS cation exchange column (GE Life Sciences) and eluted with a linear gradient to 50 mM sodium acetate pH 4.5, 1000 mM NaCl. The fractions containing Lys63 ^{15}N, ^{13}C-diUb were pooled, concentrated and flash frozen. The phosphorylation of 80 µM Lys63 ^{15}N, ^{13}C-diUb was performed with 2.5 µM *Ph*PINK1 as described above and monitored

in ^{1}H, ^{15}N-2D BEST-TROSY experiments. Upon disappearance of the wt Ser65 signal the reaction was quenched by adding 20 µM EDTA. Longer chains (triUb, tetraUb) were purified and phosphorylated identically.

Solution studies of phosphoUb

All NMR experiments were performed in phosphate buffered saline (18 mM Na_2HPO_4, 7 mM NaH_2PO_4 pH 7.2, 150 mM NaCl).

NMR acquisition was carried out at 298 K on Bruker Avance III 600 MHz and Avance2+ 700 MHz spectrometers equipped with cryogenic triple resonance TCI probes. Topspin (Bruker) and Sparky (Goddard & Kneller, UCSF; http://www.cgl.ucsf.edu/ home/sparky/) software packages were used for data processing and analysis, respectively. ^{1}H,^{15}N 2D BEST-TROSY experiments (Favier & Brutscher, 2011) were acquired with in-house optimized Bruker pulse sequences incorporating a recycling delay of 400ms and 512*64 complex points in the ^{1}H,^{15}N dimension, respectively. High quality 2D data sets were acquired in ~8 min.

Backbone chemical shift assignments were completed using Bruker triple resonance pulse sequences. CBCACONH and HNCACB spectra were collected with 1024*32*55 complex points in the ^{1}H, ^{15}N and ^{13}C dimensions. HNCO and HNCACO experiments were collected with Non Uniform Sampling (NUS) at a rate of 25% of 1024*50*47 complex points in the ^{1}H, ^{15}N and ^{13}C dimensions, respectively. HA and HB proton shifts were obtained from an HBHACONH spectrum collected with 50% NUS and 512*40*80 in the ^{1}H, ^{15}N and indirect ^{1}H dimensions, respectively. These data sets were processed with Multi-Dimensional Decomposition or Compressed Sensing using the MddNMR software package (Orekhov & Jaravine, 2011; Kazimierczuk & Orekhov, 2011).

Weighted chemical shift perturbation calculations were completed using the equation $\sqrt{(\Delta^{1}H)^2+((\Delta^{15}N)^2/5)}$.

Secondary structure calculations were completed using TALOS+ (Shen *et al*, 2009) incorporating HN, N, CA, CB and HA shifts.

$^{15}N\{^{1}H\}$-heteronuclear NOE (hetNOE) measurements were carried out using a Bruker pseudo 3D pulse program, applying a 120° ^{1}H pulse train with a 5 ms interpulse delay for a total of 5 s interleaved on- or off-resonance saturation.

The hetNOE values were calculated from peak intensities according the equation I_{on}/I_{off}.
The rate of exchange between the major and minor forms of phosphoUb was established using ZZ exchange spectroscopy. Mixing times of 6, 18, 30, 48, 66, 96, 132, 192, 258, 324, 372, 426 and 492 ms were used in the pseudo 3D data set. Peak intensities of the major and minor forms (auto) and their exchange peaks (cross) of Ile23, Phe45, Ser57 and Leu73 were fitted in Mathematica 9 (Wolfram) using the methods described in (Latham *et al*, 2009).
Differences in the hydrogen bonding network were established using the long range TROSY-based HNCO (trHNCO) experiment described by (Cordier *et al*, 2008). A 1 mM sample of ^{15}N,^{13}C phosphoUb was used to collect a 3D version of the long range trHNCO (133 ms N-C' magnetization transfer time) with 128 scans, 25% NUS and 1024*32*55 complex points in the ^{1}H, ^{15}N and ^{13}C dimensions (~6 days). Cross peaks were assigned with reference to a standard trHNCO data set, with 8 scans and 33 ms N-C' magnetization transfer time, and processed as above.
The altered hydrogen bonding network was further confirmed by analysis of a ^{15}N-edited 3D NOESY, collected on the 1 mM sample with 30% NUS and 1024*48*128 complex points in the direct ^{1}H, ^{15}N and indirect ^{1}H dimensions respectively.
Minimal maps of chemical shift perturbations (CSP) were created to compare the unassigned BEST-TROSY spectra of phosphomimetic ^{15}N-labelled Ub S65E and wild type Ub. The weighted chemical shift difference was calculated for each assigned peak of wild type Ub and all peaks in the Ub S65E spectrum, using the equation $\sqrt{(\Delta^{1}H)^2+((\Delta^{15}N)^2/5)}$. The smallest value was then reported.

Stability measurements of Ub and phosphoUb

Differential scanning calorimetry (DSC) was performed using a Microcal Capillary DSC instrument. Samples of Ub and phosphoUb were dialyzed into standard NMR buffer (25 mM phosphate, 150 mM NaCl, pH 7.2) and scanned at a heating rate of 90 °C / hour in mid feedback mode. Sample rescans

indicated significant levels of reversibility for thermal denaturation with > 50% of signal recovered despite heating initial runs to 115 °C. Data were corrected for instrumental baseline using buffer scans recorded immediately before Ub runs. After concentration normalization the intrinsic protein baseline between pre and post transitional levels was corrected using the progress function in the Origin software supplied with the instrument. Corrected endotherms were fit to a non-two state model allowing T_m, ΔH calorimetric and ΔH van't Hoff to vary independently.

Crystallographic analysis of phosphoUb

PhosphoUb for crystallization was generated by setting up a phosphorylation reaction at room temperature with 10 mg/ml recombinant Ub and 23.3 μM GST-*Ph*PINK1 in 10 mM ATP, 40 mM Tris pH 7.5, 10 mM $MgCl_2$, 0.6 mM DTT (total volume 250 μl). After 4 h the reaction was applied to 400 μl Glutathione Sepharose 4B beads (GE Healthcare) and agitated for 1 h. The flow through was collected and buffer exchanged into water with a PD-10 desalting column (GE Healthcare) and applied on a MonoQ anion exchange column (GE Life Sciences). Pure phosphoUb eluted in 50 mM Tris pH 7.4 and, was concentrated in a 3-kDa MWCO spin concentrator (Sartorius), and crystallized at 3 mg/ml using the vapor diffusion method in sitting drop experiments. Crystals grew in 30% (w/v) PEG 8000, 0.2 M ammonium sulfate, and were vitrified in mother liquor containing 25.5% (w/v) PEG 8000, 0.17 M ammonium sulfate, 15% (v/v) glycerol.

Data were collected at the Diamond Light Source (Harwell, UK) beamline I-03. The structure was solved by molecular replacement in Phaser (McCoy *et al*, 2007), using truncated Ub (pdb-id 1UBQ, residues 1-71) as a search model. Subsequent rounds of model building in coot (Emsley *et al*, 2010) and refinement in Phenix (Adams *et al*, 2011) generated a final model with statistics shown in **Supplementary Table 1**. All structure figures were done in PyMol (www.pymol.org) and electrostatics were calculated with CHARMM (http://www.charmm-gui.org).

Ubiquitin chain composition mass spectrometry analysis

Chain assembly reactions were resolved on NuPAGE 4-12% gradient Bis-Tris gel prior to in-gel digestion and the addition of 400 fmoles AQUA peptide standards according to (Kirkpatrick *et al*, 2006) and (Ordureau *et al*, 2014). **Supplementary Table 1** contains a list of all AQUA peptide standards. Isotopically labeled AQUA peptide standards were synthesized and purchased from Cell Signalling Technology®. Extracted peptides were lyophilized and stored at -80°C. Prior to MS analysis, peptides were resuspended in 30 µl of reconstitution buffer (7.5% ACN, 0.5% TFA, 0.01% H_2O_2). Oxidation of methionine-containing peptides was performed according to (Phu *et al*, 2010). 10 µl was directly loaded onto an EASY-Spray reverse-phase column via partial loop injection (C18, 3µm, 100Å, 75µm x 15µm) using a Dionex UltiMate 3000 HPLC system (Thermo Fisher Scientific). Peptides were eluted using a 25 min ACN gradient (2.5-35%) at a flow rate of 1.4 µl min^{-1}and flowmeter pressure of ~6,500 psi. Peptides were analyzed on a Q-Exactive mass spectrometer (Thermo Fisher Scientific) using parallel reaction monitoring (PRM), similar to (Tsuchiya *et al*, 2013). For PRM assays, monoisotopic precursor masses were isolated (2 *m/z* window) and fragmented at predetermined chromatographic retention times. Precursor masses were fragmented using the following settings: resolution, 17,500; AGC target, 1E5; maximum injection time, 120 ms; normalized collision energy, 28. Raw files were searched and fragment ions quantified using Skyline version 2.5.0.6157© (MacLean *et al*, 2010). The fragment ions used for quantitation are listed in **Supplementary Table1**. Data generated from Skyline was exported into a Microsoft Excel spread sheet for further analysis according to (Kirkpatrick *et al*, 2006).

Ubiquitin chain assembly studies

For E2 charging assays, 250 nM E1 were mixed with 4 µM E2 enzymes and 15 µM Ub or phosphoUb in ligation buffer (40 mM Tris pH 7.5, 10 mM magnesium chloride, 0.6 mM dithiothreitol) and incubated at 37 ºC. At indicated timepoints, 9 µl samples were mixed with 9 µl 4xLDS loading buffer (Invitrogen) without reducing agent, and resolved on 4-12% gradient SDS

PAGE gels in MES buffer (Invitrogen). Gels were Coomassie-stained with InstantBlue (Expedeon).

For ligase reactions, 5-10 µM of respective E3 ligases were added to the E2 mixture. Western blotting was performed using rabbit polyclonal anti-Ub antibody (Millipore).

UBD pull-down assay

Pull-down assays were essentially performed as previously described(Kulathu *et al*, 2009). 30 µg of GST-tagged TAB2 NZF was immobilized on 25 µl of Glutathione Sepharose 4B (GE Life Sciences) and washed three times with pull-down assay buffer (PDAB; 50 mM Tris pH 7.4, 150 mM NaCl, 2 mM β-mercaptoethanol, 0.1 % NP-40). Then, 1.5 µg of the indicated tetraUb species (see section above; Generation of phosphoUb) was incubated with the immobilized TAB2 NZF overnight at 4 ºC in a total volume of 450 µl pull-down assay buffer containing 0.2 mg/ml BSA. The beads were then washed five times with PDAB prior to separation by SDS-PAGE. Proteins were transferred to PVDF and blotted using a polyclonal rabbit anti-Ub antibody (Millipore).

Disassembly of phosphorylated polyubiquitin

DUBs were either kind gifts from Marc Pittmann, purchased, or purified according to published procedures (Mevissen *et al*, 2013). Polyubiquitinated cIAP substrate was generated from a ligase reaction with GST-tagged cIAP1 and UBE2D1, which was stopped with 0.1 U apyrase. 10 µl of this reaction were used in a 30 µl DUB reaction, that contained 3 µl 10 x DUB buffer (500 mM sodium chloride, 500 mM Tris pH 7.5, 50 mM dithiothreitol) and DUBs at indicated concentrations. During incubation at 37 ºC, aliquots of 6 µl of the reaction were taken at the time points indicated and mixed with 6 µl 4 x LDS loading buffer (Invitrogen) to stop the reaction. Samples (10 µl) were resolved by SDS-PAGE as above and silver stained using the Bio-Rad Silver Stain Plus kit according to the manufacturer’s protocol.

DiUb and tetraUb chains were phosphorylated with GST-*Ph*PINK1 and Ser65 phosphorylation efficiency was assessed by either AQUA MS (tetraUbs) or ESI-MS (K6 diUb). GST-*Ph*PINK1 was removed by Glutathione 4B Sepharose beads. Ub chains were diluted in PBS + 5 mM DTT at 1 µM (1.5x, tetraUb) or

3 µM (1.5x, diUb), DUBs were diluted to their respective 3x concentrations in PBS + 5 mM DTT and both solutions were incubated at 37 ºC for 10 min prior to start of the reaction. Samples were taken after the indicated time points and quenched by addition 4xLDS loading buffer (Invitrogen), and analyzed by SDS-PAGE and silver staining as described above.

SUPPLEMENTARY REFERENCES

Adams PD, Afonine PV, Bunkóczi G, Chen VB, Echols N, Headd JJ, Hung L-W, Jain S, Kapral GJ, Grosse Kunstleve RW, McCoy AJ, Moriarty NW, Oeffner RD, Read RJ, Richardson DC, Richardson JS, Terwilliger TC & Zwart PH (2011) The Phenix software for automated determination of macromolecular structures. *Methods* **55:** 94–106

Cordier F, Nisius L, Dingley AJ & Grzesiek S (2008) Direct detection of N-H[...]O=C hydrogen bonds in biomolecules by NMR spectroscopy. *Nat Protoc* **3:** 235–241

Dou H, Buetow L, Sibbet GJ, Cameron K & Huang DT (2012) BIRC7-E2 ubiquitin conjugate structure reveals the mechanism of ubiquitin transfer by a RING dimer. *Nat Struct Mol Biol* **19:** 876–883

Emsley P, Lohkamp B, Scott WG & Cowtan K (2010) Features and development of Coot. *Acta Crystallogr D Biol Crystallogr* **66:** 486–501

Favier A & Brutscher B (2011) Recovering lost magnetization: polarization enhancement in biomolecular NMR. *J Biomol NMR* **49:** 9–15

Hospenthal MK, Freund SMV & Komander D (2013) Assembly, analysis and architecture of atypical ubiquitin chains. *Nat Struct Mol Biol* **20:** 555–565

Juang Y-C, Landry M-C, Sanches M, Vittal V, Leung CCY, Ceccarelli DF, Mateo A-RF, Pruneda JN, Mao DYL, Szilard RK, Orlicky S, Munro M, Brzovic PS, Klevit RE, Sicheri F & Durocher D (2012) OTUB1 Co-opts Lys48-Linked Ubiquitin Recognition to Suppress E2 Enzyme Function. *Mol Cell* **45:** 384–397

Kamadurai HB, Souphron J, Scott DC, Duda DM, Miller DJ, Stringer D, Piper RC & Schulman BA (2009) Insights into ubiquitin transfer cascades from a structure of a UbcH5B approximately ubiquitin-HECT(NEDD4L) complex. *Mol Cell* **36:** 1095–1102

Kazimierczuk K & Orekhov VY (2011) Accelerated NMR spectroscopy by using compressed sensing. *Angew Chem Int Ed Engl* **50:** 5556–5559

Keusekotten K, Elliott PR, Glockner L, Fiil BK, Damgaard RB, Kulathu Y, Wauer T, Hospenthal MK, Gyrd-Hansen M, Krappmann D, Hofmann K & Komander D (2013) OTULIN antagonizes LUBAC signaling by specifically hydrolyzing Met1-linked polyubiquitin. *Cell* **153:** 1312–1326

Kirkpatrick DS, Hathaway NA, Hanna J, Elsasser S, Rush J, Finley D, King RW & Gygi SP (2006) Quantitative analysis of in vitro ubiquitinated cyclin B1 reveals complex chain topology. *Nature Cell Biology* **8:** 700–710

Kulathu Y, Akutsu M, Bremm A, Hofmann K & Komander D (2009) Two-sided ubiquitin binding explains specificity of the TAB2 NZF domain. *Nat Struct Mol Biol* **16:** 1328–1330

Latham MP, Zimmermann GR & Pardi A (2009) NMR chemical exchange as a probe for ligand-binding kinetics in a theophylline-binding RNA aptamer. *J Am Chem Soc* **131:** 5052–5053

MacLean B, Tomazela DM, Shulman N, Chambers M, Finney GL, Frewen B, Kern R, Tabb DL, Liebler DC & MacCoss MJ (2010) Skyline: an open source document editor for creating and analyzing targeted proteomics experiments. *Bioinformatics* **26:** 966–968

Maspero E, Mari S, Valentini E, Musacchio A, Fish A, Pasqualato S & Polo S (2011) Structure of the HECT:ubiquitin complex and its role in ubiquitin chain elongation. *EMBO Rep* **12:** 342–349

McCoy AJ, Grosse Kunstleve RW, Adams PD, Winn MD, Storoni LC & Read RJ (2007) Phaser crystallographic software. *J Appl Crystallogr* **40:** 658–674

Mevissen TET, Hospenthal MK, Geurink PP, Elliott PR, Akutsu M, Arnaudo N, Ekkebus R, Kulathu Y, Wauer T, Oualid El F, Freund SMV, Ovaa H & Komander D (2013) OTU Deubiquitinases Reveal Mechanisms of Linkage Specificity and Enable Ubiquitin Chain Restriction Analysis. *Cell* **154:** 169–184

Ordureau A, Sarraf SA, Duda DM, Heo J-M, Jedrychowski MP, Sviderskiy VO, Olszewski JL, Koerber JT, Xie T, Beausoleil SA, Wells JA, Gygi SP, Schulman BA & Harper JW (2014) Quantitative Proteomics Reveal a Feedforward Mechanism for Mitochondrial PARKIN Translocation and Ubiquitin Chain Synthesis. *Mol Cell*

Orekhov VY & Jaravine VA (2011) Progress in Nuclear Magnetic Resonance Spectroscopy. *Progress in Nuclear Magnetic Resonance Spectroscopy* **59:** 271–292

Phu L, Izrael-Tomasevic A, Matsumoto ML, Bustos DJ, Dynek JN, Fedorova AV, Bakalarski CE, Arnott D, Deshayes K, Dixit VM, Kelley RF, Vucic D & Kirkpatrick DS (2010) Improved quantitative mass spectrometry methods for characterizing complex ubiquitin signals. *Mol Cell Proteomics*

Sato Y, Yoshikawa A, Yamagata A, Mimura H, Yamashita M, Ookata K, Nureki O, Iwai K, Komada M & Fukai S (2008) Structural basis for specific cleavage of Lys 63-linked polyubiquitin chains. *Nature* **455:** 358–362

Shen Y, Delaglio F, Cornilescu G & Bax A (2009) TALOS+: a hybrid method for predicting protein backbone torsion angles from NMR chemical shifts.

J Biomol NMR **44:** 213–223

Stieglitz B, Rana RR, Koliopoulos MG, Morris-Davies AC, Schaeffer V, Christodoulou E, Howell S, Brown NR, Dikic I & Rittinger K (2013) Structural basis for ligase-specific conjugation of linear ubiquitin chains by HOIP. *Nature* **503:** 422–426

Tsuchiya H, Tanaka K & Saeki Y (2013) The parallel reaction monitoring method contributes to a highly sensitive polyubiquitin chain quantification. *Biochem Biophys Res Commun* **436:** 223–229

Weeks SD, Grasty KC, Hernandez-Cuebas L & Loll PJ (2011) Crystal structure of a Josephin-ubiquitin complex: evolutionary restraints on ataxin-3 deubiquitinating activity. *J Biol Chem* **286:** 4555–4565

Wiener R, Zhang X, Wang T & Wolberger C (2012) The mechanism of OTUB1-mediated inhibition of ubiquitination. *Nature* **483:** 618–622

Ye Y, Akutsu M, Reyes-Turcu F, Enchev RI, Wilkinson KD & Komander D (2011) Polyubiquitin binding and cross-reactivity in the USP domain deubiquitinase USP21. *EMBO Rep* **12:** 350–357
