# Supplementary material for: Ubiquitin Ser65 phosphorylation affects ubiquitin structure, chain assembly and hydrolysis
Source: EMBO J. 2014 Dec 20;34(3):307–25. doi: 10.15252/embj.201489847 (PMC4339119; doi:10.15252/embj.201489847)
Supplement: Supplementary file 2 [file embj0034-0307-sd2.pdf]

THE EMBO JOURNAL

Manuscript EMBO-2014-89847

Ubiquitin Ser65 phosphorylation affects ubiquitin structure, chain assembly and hydrolysis

Tobias Wauer, Kirby N. Swatek, Jane Wagstaff, Christina Gladkova, Jonathan N. Pruneda, Martin A. Michel, Malte Gersch, Christopher M. Johnson, Stefan M.V. Freund and David Komander

Corresponding author: David Komander, MRC Laboratory of Molecular Biology

Review timeline:

Submission date:	18 August 2014
Editorial Decision:	10 September 2014
Revision received:	13 November 2014
Accepted:	21 November 2014

Editor: Hartmut Vodermaier/Andrea Leibfried

Transaction Report:

(Note: With the exception of the correction of typographical or spelling errors that could be a source of ambiguity, letters and reports are not edited. The original formatting of letters and referee reports may not be reflected in this compilation.)

1st Editorial Decision 10 September 2014

Thank you again for submitting your manuscript on ubiquitin phosphorylation, and please excuse the delay in contacting you with a decision (Andrea is on vacation and I am myself also currently away from the office). We have now obtained three referee reports, which you will find copied below.

As you will see, the referees find your study interesting and timely, and we would therefore remain interested in considering a revised manuscript further for publication. Nevertheless, it is apparent that all referees raise some concerns regarding the depth of the biochemical analyses, and the level of insight into the functional significance of some of the findings. As you will see, they offer some straightforward suggestions and avenues for strengthening the respective aspects of the paper, which we would invite you to address in a revised version of the study. Furthermore, we would also encourage the inclusion of at least some further data addressing the significance of your findings in a physiologically relevant context such as parkin function/mitochondrial depolarization.

Since we understand that addressing every single point with further experiments might not be realistic within the scope of the present manuscript, we would in this case appreciate if you would send us a brief proposal (in the form of a tentative response letter) on how you might be able to address the referees' comments, so that we could discuss which experiments and changes would be feasible, and what would be key for eventual acceptance in The EMBO Journal. Please note that we would also be open to discussing an extended revision period, during which time the publication of any competing work elsewhere would have no negative impact on our final assessment of your own study.

Thank you again for the opportunity to consider this work for publication, and we look forward to hearing from you regarding your suggestions for revising the manuscript.

© European Molecular Biology Organization

REFEREE REPORTS:

Referee #1:

The manuscript by Wauer and colleagues reports a wide-ranging series of experiments with phosphoubiquitin and phosphoubiquitin chains. Among the key findings are that Lys63-linked hexaUb and Lys11-linked tetraUb and by phosphorylated by the kinase PINK1. Using X-ray crystallography and NMR spectroscopy, the authors determine the structures of two conformations of phosphoubiquitin, one of which is significantly different from unphosphorylated ubiquitin. The authors conclude by showing that while most enzymes of the ubiquitin system do not distinguish between phosphoubiquitin and ubiquitin, several are strongly inhibited by phosphoubiquitin. Among a panel of 13 E2 enzymes, UBE2E1, UBE2N and UBE2T, showed decreased activity for forming polyubiquitin chains from phosphoubiquitin. In a similar vein, the E3 ligase cIAP was found to be non-specific while the E3s TRAF6 and HOIP were inactive with phosphoubiquitin. The authors also tested two deubiquitinating enzymes USP2 and USP21 and showed that the former was inactive in hydrolyzing phosphoubiquitin chains on cIAP.

The paper follows the recent, high profile discovery by three groups of the activation of the E3 ligase parkin by phosphoubiquitin and addresses several key questions such as the activity of PINK1 on polyubiquitin chains and the ability of phosphoubiquitin to be assembled into polyubiquitin chains. The significance of the structural finding of a second form of phosphoubiquitin is less clear. The authors show that the two forms interconvert and suggest that the ability of phosphoubiquitin to participate in many ubiquitination reactions implies the major form is present. However, which form binds and activates parkin remains an open question - the identification of the minor conformation is certainly intriguing.

The manuscript is a significant body of work and generally appears suitable for publication. The only possible issue is the failure of the authors to characterize phosphoubiquitin for parkin activity. Neither cIAP nor TRAF6 have been linked to phosphoubiquitin signalling and given the very small amount of phosphoubiquitin present in cells, the relevance of their activity (or inactivity) with phosphoubiquitin is unclear. While Koyano have shown that parkin activates discharging of phosphoubiquitin from the E2 UBCH7, little is known about how phosphobubiquitin might be incorporated into polyubiquitin chains by parkin. Similarly, the hydrolysis of phosphoubiquitin chains by deubiquitinases such as USP15 and USP30 that are involved in Parkinson's disease would be more relevant than the reported results with USP2 and USP21. Characterization of the effect of phosphoubiquitin on enzymes that are physiologically relevant for mitophagy or Parkinson's disease would improve the focus the manuscript.

Minor issues:

The abstract requires minor edits.

"The protein kinase PINK1 was recently shown to phosphorylate ubiquitin (Ub) Ser65, and Ser65 phospho-ubiquitin (phosphoUb) activates the E3 ligase Parkin." => "The protein kinase PINK1 was recently shown to phosphorylate ubiquitin (Ub) on Ser65, which directly activates the E3 ligase Parkin."

"We here show that PINK1 can phosphorylate polyUb chains and that Ub Ser65 phosphorylation alters Ub structure, generating two independent conformations of phosphoUb in solution." => "Here, we show that PINK1 can phosphorylate polyUb chains and that Ub Ser65 phosphorylation alters Ub structure, generating two conformations in solution."

"We further show that phosphoUb has no effect on E1-mediated E2 charging, however can affect discharging of E2 enzymes to form polyUb chains." => "We further show that phosphoUb has no effect on E1-mediated E2 charging; however, it can affect discharging of E2 enzymes to form polyUb chains."

pg 3. "...dopaminergic neurons that affects" => "...dopaminergic neurons which affects"

pg 3 "Mutations in the Ub E3 ligase Parkin predisposes" => "Mutations in the Ub E3 ligase Parkin predispose"

pg 3. "... autosomal recessive juvenile Parkinsonism (AR-JP) where neurological symptoms show at an early age. Heterozygous mutations of Parkin have also been implicated in the sporadic, late-onset form of the disease"

=> "... autosomal recessive juvenile Parkinsonism (AR-JP) a form of the disease where neurological symptoms show at an early age. Heterozygous mutations of Parkin have also been implicated in the more common, late-onset form of the disease"

pg 3. It would seem appropriate to cite NC Chan (2011) Hum Mol Gen for the earlier identification of parkin substrates: "Once activated, Parkin ubiquitinates numerous mitochondrial and cytosolic proteins (Sarraf et al, 2013) including mitofusins and Miro1, eventually triggering mitophagy (Youle & Narendra, 2011)."

pg 4. "These include significant structural changes within phosphoUb that adopts two conformations in solution, one of which has a retracted C-terminal tail and has not been observed to date." => "These include significant structural changes within phosphoUb wherein the protein adopts two conformations in solution, one of which has a retracted C-terminal tail and has not been observed to date.""

pg 4. Who is "together"? The authors? Suggest changing "Together, we show" => "Together, our results show" or "In summary, we show"

pg 4. "In these reactions and as seen by others, Ub was phosphorylated, and phosphorylation occurred exclusively at Ser65." => "In these reactions and as seen by others, Ub was phosphorylated exclusively at Ser65."

pg 6. "However, secondary structure calculations for the major and minor species using TALOS+". Could the authors comment about the changes observed for residues 39 and 40 in the minor form?

pg 6. "This is consistent with having two distinct conformations, i.e., a major native-like and a minor species of phosphoUb, in slow exchange via a less stable intermediate." I do not see why the reduced stability of the phosphorylated form is relevant for the observation of two forms or their exchange.

pg 8. "Interestingly, Arg74 is significantly stabilized in the minor species." The largest change in Supplementary Figure 7 is at residue 65. Could the authors speculate why pS65 is less mobile in the extended loop of the minor form?

pg 10. "Ubiquitin is almost invariant in evolution, is identical in higher eukaryotes, and differs only by two conservative mutations between Saccharomyces cerevisiae and humans." The statement is incorrect. There are three substitutions. Either say "three mutations" or "two conservative and one non-conservative mutation"

pg 10. "The structural data described above (Figure 2-4) shows that phosphoUb is indeed structurally enabled to perform alternative functions," What functions? What does "structurally enabled" mean in the absence of any clear function?

pg 18. "whereby ~2.5% of total Ub and 10-20% of mitochondrial Ub is being phosphorylated after mitochondrial depolarization (Alban Ordureau and Wade Harper, personal communication)." Rather than citing unpublished work, it would be better to use the published value of 3% reported by Koyano (2014) Nature.

pg 18. "Nonetheless, the pool of free phosphoUb may be charged onto E2s, and affect other processes, potentially affecting other Parkin dependent biology" This seems like speculation given that the levels of free phosphoubiquitin are vanishingly small (0.05% in the absence of

overexpressed PINK1) and that there is no evidence so far that any enzyme preferentially uses phosphoubiquitin.

Fig 6C & D. I didn't see the justification for using different colors for K63 chains and K63pS65 chains. I understand that the peptides are distinct but while reporting for the intact protein, the same logic would suggest that K48 chains should be colored differently between the Ub and phosphoUb experiments. Having blue and green gives the impression that there is a difference in chain types when in fact there is not.

Fig 6H. Are the chains generated in the presence of phophoUb significant? Are the 4% of chains due to TRAF6 activity?

Referee #2:

Recently, phosphorylation of Ser65 in ubiquitin by PINK1 was shown to be a critical step in Parkin E3 Ub ligase activation. These findings also called attention to the function of phosphorylation of Ub. Here, the authors provide some very interesting and timely information on how Ser65 phosphorylation could affect Ub function from a structural and biochemical perspective. Importantly, they showed that PINK1 can phosphorylate K63- and K11-linked polyUb chains as well as free Ub. Based on NMR analysis, pSer65 Ub appears to have two independent conformations in solution. The major pSer65 Ub conformation is similar to that of unphosphorylated ubiquitin but has altered surface charge properties. The minor form has a two-residue 5-strand slippage that pulls the C-terminal tail back into the Ub core structure, and exposes pS65 on a loop. The authors further showed that phosphoUb did not affect E1-mediated E2 charging, but did have effects on discharging of a subset E2~Ub enzymes to form polyUb chains. Interestingly, UBE2N/UEV1A and TRAF6/UBE2D-mediated K63-chain assembly and HOIP-mediated M1 linear chain assembly were all inhibited by pSer65 Ub. Moreover, phosphoUb K63 chains were resistant to the cleavage by the USP2 deubiquitylase, but not the closely related USP21 DUB. The authors conclude that phosphorylation of S65 can have major functional consequences on specific ubiquitin pathway functions, and particular K63 Ub chain formation by certain E3 ligases.

Overall, the work is technically outstanding, and the observed effects on Ub structure and K63 chain formation will be enlightening to the ubiquitin and Parkin communities. Unfortunately, they were not able to demonstrate the functional significance of the minor form of pS65 Ub they found, although this will be challenging due to the equilibrium between the two states. It would have strengthened their story, if they had been able to show that pS65-K63 chains accumulated in association with damaged mitochondria in response to depolarization, using MS and their pS65-K63 AQUA standard peptide (see minor point 1 below). If such pS65 K63-Ub chains do accumulate on damaged mitochondria and they are partially resistant to DUB activity, as shown here in vitro, this could serve as a means to amplify the signal for recognition by autophagy receptors (however, if this recognition involves a K63-specific Ub binding domain, this interaction would have to be unaffected by phosphate at S65 (see point 2).

Some consideration of the following additional points would strengthen the paper.

1. Can Pink1 also phosphorylate K48-linked Ub chains?

2. In terms of the possible functional consequences of assembly/phosphorylation of K63 Ub chains, one would like to know whether phosphorylation of K63-linked Ub chains affects the binding of K63-linkage specific ubiquitin-binding domains?

3. Does pS65 Ub affect the linkage type of Parkin-mediated ubiquitin chains assembled in vitro? Since Ub phosphorylation by PINK1 is key to Parkin function in mitophagy, this seems like an obvious experiment to do.

4. Can pSer65Ub adopt the minor conformation when present in chains?

5. Do the authors think that E3's and DUBs will in principle be able to act on pSer65 Ub when it is in the minor conformation or does it have to flip back to the major conformation?

6. Were any molecular dynamics simulations carried out to study the transition between the two pS65 Ub conformations? MD simulation might also indicate if unphosphorylated Ub can also adopt this altered conformation? This could be relevant to the ability of PINK1 to phosphorylate S65, since S65 is better exposed in the minor conformation for PINK1 phosphorylation. In the major Ub conformation S65 does not appear to be readily accessible for phosphorylation by PINK1, which is relevant because ePK catalytic domains generally require the peptide backbone around the substrate hydroxyamino acid to be in an extended conformation.

Minor points: 1. Figure 1: In this regard, the authors need to indicate exactly what pS65/K63 AQUA peptide was used and how it was made. For anything other than pS65 K63-linked Ub chains a Ser65 phosphorylated T55-K63 peptide would be the control standard, but for pS65 K63-linked Ub chains they would need a pS65-K63(GG) AQUA peptide, which is what would be obtained upon tryptic digestion. The authors need to clarify exactly what was done to quantify the extent of Ser65 phosphorylation in the experimennts where they phosphorylated pre-assembled Ub chains.

2. Figure 3: How far does the K63 -NH2 group "move" in the pSer65 structure?

3. Figure 6: It looks as though GST-cIAP1 utilized pSer65 Ub for chain formation more efficiently than unphosphorylated Ub.

4. Does pSer65 Ub compete with unphosphorylated Ub for K63 chain formation by E3 ligases such as UBE2N/UEV1A and TRAF6/UBE2D?

5. With regard to the solution dynamics of the Ub fold the authors could also cite Vˆgeli et al. (J. Am. Chem. Soc. 131:17215).

Referee #3:

Wauer et al. present a structural and biochemical study of ubiquitin phosphorylated at Ser65 - a modification recently described as sufficient for activation of the parkin E3 ligase. In vivo, Ub is phosphorylated by the PINK1 kinase, and the authors reconstitute this activity in vitro to explore how Ub phosphorylation alters its structure via NMR and crystallography and its ability to be recognized by Ub conjugation and deconjugation machinery. While phosphoUb is recognized by the E1 enzyme, most E2 enzymes and some E3s, some E2 enzymes cannot form Ub chains using phosphoUb. Additionally, one USP DUB (USP2) is shown to have a minimal defect in processing Ub chains formed by phosphoUb. The study appears technically sound, with only a few inconsistencies (see below), and it offers an unexpected finding based on NMR that phosphoUb can adopt two conformations that are in equilibrium, a major conformation that resembles wild-type Ub and an unanticipated minor conformational wherein the last beta-strand slips into the Ub fold, effectively shortening the C-terminal tail. While these observations are of some interest, the study is largely descriptive and offers little biological insight into the role of phosphoUb or the unanticipated minor conformation. Furthermore, the biochemical data, as presented, appear somewhat preliminary, a feature of the manuscript that is somewhat offset by the mass spec analysis.

Major points:

The major/minor NMR solution structures for phosphoUb are surprising, however they are in equilibrium so it remains unclear if the minor conformation contributes to any of the associated biology/biochemistry. As no biological role is offered for the minor conformation the authors can only speculate on the existence of receptors, etc. While some E2s/E3s/DUBs can process phosphoUb, others can't, but there is no connection to whether this is due to the major/minor conformations or if the altered electrostatic surface properties of phosphoUb are responsible. As the latter case is deemed more likely, the authors should augment the biochemical studies using a phosphomimetic of phosphoUb to determine if it recapitulates E1/E2/E3/DUB activities with phosphoUb.

Given the available toolkit of DUBs in the Komander lab, a more thorough analysis of DUBs might be expected. Additionally, USP2 (which the authors claim shows reduced cleavage of phosphUb)

© European Molecular Biology Organization

goes form barely any cleavage (Figure 7B) to respectable cleavage (Suppl Fig 10G) by a two-fold increase in enzyme concentration - a result that warrants further investigation/comments. Suppl Fig 10G is not included in the legend and is not referred to in the text. Perhaps it is a typo/mis-label of the panels?

Minor comments:

Figure 1 - Why has analysis of K48 chains (a relevant chain type for parkin function) not been included? panel Figure 1B, the figure should indicate that it is a phostag gel (not only the legend).

When discussing Figure 3 (page 7), there is no mention of chains G/H in the crystal structure (not utilizing the Ile44 patch). What crystal contacts are they making?

Figure 5, panel A - what does the AQUA profile reveal for Cdc34?

Figure 6, the use of bar graphs and pie charts appears redundant in panels 6C/D and 6G/H

Figure 6, what is the effect of phosphoUb on parkin activity in vitro? This might also provide insight into phosphoUb on K48 chain formation.

Supplementary Figure 10F and 10G - the inputs for the DUB reaction between Ub and phosphoUb are not the same, making comparisons difficult. The position of the bands for different DUBs should be labeled on the gels.

Supplementary Figure 10G - the legend is missing or mislabeled.

Discussion section (page 18, end of top paragraph). It may be relevant to discuss the activities of existing DUB-resistant Ub mutants in the context of phosphoUb.

1st Revision - authors' response 13 November 2014

(see next page)

We would like to thank all three reviewers for their constructive feedback and the overall encouraging evaluation of our data. We do appreciate the point raised by all reviewers that Parkin and related proteins should appear more prominently in our manuscript, and in the revised version, they do. However, the focus of this study was not Parkin, simply because we wanted to take a global perspective on the ubiquitin system and the implications of phosphoubiquitin, and also because we are working on understanding how phosphoubiquitin affects Parkin function which is focus of a separate, unsubmitted manuscript.

Nonetheless, we have addressed all comments by the reviewers, which has significantly improved the manuscript.
New data in the first part of the manuscript now shows that

- PhosphoUb activates Parkin (Fig. 1). However, when only phosphoUb is used in the reaction, Parkin is inactive. Ordureau et al also published the latter result recently (Mol Cell, Oct 2014). We do not have an explanation for it yet.
- PINK1 phosphorylates all available tetraUb molecules (now also K48, K6, linear, K33) in vitro (**Figure 1**).
- S65E Ub does not have a second minor conformation but resembles the major conformation (new **Supp Fig. 12**)
- The minor conformation also exists in context of K63 chains (new **Supp Fig. 6**).
- The structure of the minor conformation with strand slippage is also consistent with new long range NOE data (new **Supp Fig. 9**)

We have also improved the second, biochemical part of the manuscript.

- Prompted by Ref 3, we re-evaluated cdc34 activity with phosphoUb, showing that while it is charged with phosphoUb, its ability to produce free chains in vitro is reduced (**Figure 5**)
- We tested the interaction of a K63-specific UBD, TAB2, which has been used as a Ub chain sensor that localizes to mitochondria upon depolarization (van Wijk et al). K63 tetraUb and tetraphosphoUb bind TAB2 NZF similarly (new **Figure 7**).
- We have tested several additional DUBs including: USP8, USP15, USP30, Ataxin-3 for their ability to cleave poly-phosphoUb, and all are severely inhibited against this substrate. We also used purified and phosphorylated K33, K48, K63 and M1 tetraUb chains and tested the activity of TRABID, OTUB1, AMSH, and Otulin, respectively. Similarly, these DUBs were all inhibited against their preferred substrates when it was phosphorylated at Ser65. This significantly extends the DUB part of the manuscript, allowing us to conclude that phosphoUb care likely more stable as they are not cleaved by most DUBs efficiently.

Together with the improvements generated from addressing minor comments, this has significantly expanded and strengthened the manuscript as a whole.

Below, we discuss how we have addressed individual points.

Referee #1:

The manuscript by Wauer and colleagues reports a wide-ranging series of experiments with phosphoubiquitin and phosphoubiquitin chains. Among the key findings are that Lys63-linked hexaUb and Lys11-linked tetraUb and by phosphorylated by the kinase PINK1. Using X-ray crystallography and NMR spectroscopy, the authors determine the structures of two conformations of phosphoubiquitin, one of which is significantly different from unphosphorylated ubiquitin. The authors conclude by showing that while most enzymes of the ubiquitin system do not distinguish between phosphoubiquitin and ubiquitin, several are strongly inhibited by phosphoubiquitin. Among a panel of 13 E2 enzymes, UBE2E1, UBE2N and UBE2T, showed decreased activity for forming polyubiquitin chains from phosphoubiquitin. In a similar vein, the E3 ligase cIAP was found to be non-specific while the E3s TRAF6 and HOIP were inactive with phosphoubiquitin. The authors also tested two deubiquitinating enzymes USP2 and USP21and showed that the former was inactive in hydrolyzing phosphoubiquitin chains on cIAP.

The paper follows the recent, high profile discovery by three groups of the activation of the E3 ligase parkin by phosphoubiquitin and addresses several key questions such as the activity of PINK1 on polyubiquitin chains and the ability of phosphoubiquitin to be assembled into polyubiquitin chains. The significance of the structural finding of a second form of phosphoubiquitin is less clear. The authors show that the two forms interconvert and suggest that the ability of phosphoubiquitin to participate in many ubiquitination reactions implies the major form is present. However, which form binds and activates parkin remains an open question - the identification of the minor conformation is certainly intriguing.

The manuscript is a significant body of work and generally appears suitable for publication.

We would like to thank the reviewer for their support, and for their constructive feedback and their attempts to make our manuscript more accessible. This is much appreciated.

The only possible issue is the failure of the authors to characterize phosphoubiquitin for parkin activity.
Neither cIAP nor TRAF6 have been linked to phosphoubiquitin signalling and given the very small amount of phosphoubiquitin present in cells, the relevance of their activity (or inactivity) with phosphoubiquitin is unclear. While Koyano have shown that parkin activates discharging of phosphoubiquitin from the E2 UBCH7, little is known about how phosphobubiquitin might be incorporated into polyubiquitin chains by parkin.

We have now included experiments on Parkin in Figure 1, showing that phosphoUb indeed activates Parkin when used in a ratio with Ub of 80:20. This reproduces the results from the previously mentioned papers.
However, we also show that when phosphoUb is used as the sole source of Ub Parkin is catalytically inactive. This result is striking, and has also been published in the recent comprehensive study by Wade Harper's lab (Ordureau et al, Mol Cell, Oct 2014). At current we have no mechanistic explanation for this.
Still, this result warranted the study of further E3 systems, and our data that cIAP is unaffected, and that TRAF6 is also affected may provide insights into how phosphoUb effects E3 function.

Similarly, the hydrolysis of phosphoubiquitin chains by deubiquitinases such as USP15 and USP30 that are involved in Parkinson's disease would be more relevant than the reported results with USP2 and USP21.

We completely agree, and have significantly expanded the DUB figure (new **Figure 8**). We now include: USP8 (Parkin DUB, Durcan et al, EMBO J Sept 2014), USP15 (mitophagy DUB, PNAS 2014), USP30 (mitophagy DUB, Bingol et al, Nature 2014) and Ataxin-3 (Parkin DUB, Durcan et al, JBC 2012). All of which are significantly inhibited against poly-phosphoUb.

For USP30, we saw no activity against the polyUb substrate. We performed a specificity analysis to show that USP30 is most active against K6-linked diUb over other chain types. Since K6 linkages accounts for merely 1% of the total linkage composition in our cIAP assembled polyUb sample, this explains the low activity against this sample. The K6-specificity is consistent with recent data that Parkin generates significant amounts of K6-linked chains (Durcan, EMBO J 2014; Ordureau, Mol Cell 2014). Interestingly, USP30 is less active against phospho-K6-linked chains.

We also analysed a number of other OTU and JAMM DUBs against defined wt and phosphorylated tetraUb substrates. All of which have decreased activity against phosphorylated Ub chains. In total, 10 out of 12 tested DUBs show lower activity towards phosphoUb substrates. We are hence comfortable to conclude that phosphoUb chains are likely more stable in cells.

Characterization of the effect of phosphoubiquitin on enzymes that are physiologically relevant for mitophagy or Parkinson's disease would improve the focus the manuscript.

We agree, and hope that our analysis of new enzymes, especially for the DUBs, has addressed this concern.

Minor issues:

The abstract requires minor edits.

"The protein kinase PINK1 was recently shown to phosphorylate ubiquitin (Ub) Ser65, and Ser65 phospho-ubiquitin (phosphoUb) activates the E3 ligase Parkin." => "The protein kinase PINK1 was recently shown to phosphorylate ubiquitin (Ub) on Ser65, which directly activates the E3 ligase Parkin."
We have fixed this.
"We here show that PINK1 can phosphorylate polyUb chains and that Ub Ser65 phosphorylation alters Ub structure, generating two independent conformations of phosphoUb in solution." => "Here, we show that PINK1 can phosphorylate polyUb chains and that Ub Ser65 phosphorylation alters Ub structure, generating two conformations in solution."
We have fixed this.
"We further show that phosphoUb has no effect on E1-mediated E2 charging, however can affect discharging of E2 enzymes to form polyUb chains." => "We further show that phosphoUb has no effect on E1-mediated E2 charging; however, it can affect discharging of E2 enzymes to form polyUb chains."
We have fixed this.
pg 3. "...dopaminergic neurons that affects" => "...dopaminergic neurons which affects"
We have fixed this.
pg 3 "Mutations in the Ub E3 ligase Parkin predisposes" => "Mutations in the Ub E3 ligase Parkin predispose"
We have fixed this.
pg 3. "... autosomal recessive juvenile Parkinsonism (AR-JP) where neurological symptoms show at an early age. Heterozygous mutations of Parkin have also been implicated in the sporadic, late-onset form of the disease"
=> "... autosomal recessive juvenile Parkinsonism (AR-JP) a form of the disease where neurological symptoms show at an early age. Heterozygous mutations of Parkin have also been implicated in the more common, late-onset form of the disease"
We have fixed this.
pg 3. It would seem appropriate to cite NC Chan (2011) Hum Mol Gen for the earlier identification of parkin substrates: "Once activated, Parkin ubiquitinates numerous mitochondrial and cytosolic proteins (Sarraf et al, 2013) including mitofusins and Miro1, eventually triggering mitophagy (Youle & Narendra, 2011)."
We have fixed this.
pg 4. "These include significant structural changes within phosphoUb that adopts two conformations in solution, one of which has a retracted C-terminal tail and has not been observed to date." => "These include significant structural changes within phosphoUb wherein the protein adopts two conformations in solution, one of which has a retracted C-terminal tail and has not been observed to date.""
We have fixed this.

g 4. Who is "together"? The authors? Suggest changing "Together, we show" => "Together, our results show" or "In summary, we show"
We have fixed this at various points.
pg 4. "In these reactions and as seen by others, Ub was phosphorylated, and phosphorylation occurred exclusively at Ser65." => "In these reactions and as seen by others, Ub was phosphorylated exclusively at Ser65."
We have fixed this.

pg 6. "However, secondary structure calculations for the major and minor species using TALOS+". Could the authors comment about the changes observed for residues 39 and 40 in the minor form?

We have extended the TALOS+ data set with HA secondary shifts and re-run the calculations and with this additional data the residues 39 and 40 of both major and minor forms are now predicted to be random coil. The confidence of this prediction is hampered because TALOS+ requires secondary shifts for three consecutive residues, however positions 37 and 38 in ubiquitin are “NMR silent” prolines, which do not yield secondary structure information. We have amended supplementary figure 5 to reflect this accordingly.

pg 6. "This is consistent with having two distinct conformations, i.e., a major native-like and a minor species of phosphoUb, in slow exchange via a less stable intermediate." I do not see why the reduced stability of the phosphorylated form is relevant for the observation of two forms or their exchange.

The reviewer is correct and we have rewritten this paragraph.

pg 8. "Interestingly, Arg74 is significantly stabilized in the minor species." The largest change in Supplementary Figure 7 is at residue 65. Could the authors speculate why pS65 is less mobile in the extended loop of the minor form?

The hetNOE for the major form of S65 is artificially suppressed because of its proximity to the highly flexible and therefore negative peak of the C-terminal glycine residue 76 (see assignment in **Supp Fig. 2**). We have removed the value for the major form of pS65 from the figure as it is (1) misleading and (2) impossible to deconvolute the contribution of individual residues. The value for G76 is by far the dominating contribution to this overlapping peak so we have therefore chosen to include this value.

pg 10. "Ubiquitin is almost invariant in evolution, is identical in higher eukaryotes, and differs only by two conservative mutations between Saccharomyces cerevisiae and humans." The statement is incorrect. There are three substitutions. Either say "three mutations" or "two conservative and one non-conservative mutation"
We apologise for this mistake. We have fixed this issue in the text.

pg 10. "The structural data described above (Figure 2-4) shows that phosphoUb is indeed structurally enabled to perform alternative functions," What functions? What does "structurally enabled" mean in the absence of any clear function?

We have clarified what we mean by this:

The structural data described above (**Figure 2-4**) shows that phosphoUb is structurally distinct, and could potentially adopt new functions. For example, we posit that the minor species is recognized by as-yet unknown phosphoUb binding proteins.

pg 18. "whereby ~2.5% of total Ub and 10-20% of mitochondrial Ub is being phosphorylated after mitochondrial depolarization (Alban Ordureau and Wade Harper, personal communication)." Rather than citing unpublished work, it would be better to use the published value of 3% reported by Koyano (2014) Nature.

We have included the reference and also the now published study from the Harper lab.

pg 18. "Nonetheless, the pool of free phosphoUb may be charged onto E2s, and affect other processes, potentially affecting other Parkin dependent biology" This seems like speculation given that the levels of free phosphoubiquitin are vanishingly small (0.05% in the absence of overexpressed PINK1) and that there is no evidence so far that any enzyme preferentially uses phosphoubiquitin.

The Harper paper has highlighted a technical issue with the previous mass-spec characterization, such that the levels of phosphoUb are likely higher. However, we have rewritten the discussion and removed this sentence.

Fig 6C & D. I didn't see the justification for using different colors for K63 chains and K63pS65 chains. I understand that the peptides are distinct but while reporting for the intact protein, the same logic would suggest that K48 chains should be colored differently between the Ub and phosphoUb experiments. Having blue and green gives the impression that there is a difference in chain types when in fact there is not.

We completely agree and since fixed all figures accordingly. We now only refer to K63 chains and state whether the source of Ub is phosphorylated or not.

Fig 6H. Are the chains generated in the presence of phosphoUb significant? Are the 4% of chains due to TRAF6 activity?

We have performed this control. UBE2D alone does not make any chains, and hence the background activity is due to TRAF6 (new **Supp Fig 11F**) .

Referee #2:

Recently, phosphorylation of Ser65 in ubiquitin by PINK1 was shown to be a critical step in Parkin E3 Ub ligase activation. These findings also called attention to the function of phosphorylation of Ub. Here, the authors provide some very interesting and timely information on how Ser65 phosphorylation could affect Ub function from a structural and biochemical perspective. Importantly, they showed that PINK1 can phosphorylate K63- and K11-linked polyUb chains as well as free Ub. Based on NMR analysis, pSer65 Ub appears to have two independent conformations in solution. The major pSer65 Ub conformation is similar to that of unphosphorylated ubiquitin but has altered surface charge properties. The minor form has a two-residue β5-strand slippage that pulls the C-terminal tail back into the Ub core structure, and exposes pS65 on a loop. The authors further showed that phosphoUb did not affect E1-mediated E2 charging, but did have effects on discharging of a subset E2~Ub enzymes to form polyUb chains. Interestingly, UBE2N/UEV1A and TRAF6/UBE2D-mediated K63-chain assembly and HOIP-mediated M1 linear chain assembly were all inhibited by pSer65 Ub. Moreover, phosphoUb K63 chains were resistant to the cleavage by the USP2 deubiquitylase, but not the closely related USP21 DUB. The authors conclude that phosphorylation of S65 can have major functional consequences on specific ubiquitin pathway functions, and particular K63 Ub chain formation by certain E3 ligases.

Overall, the work is technically outstanding, and the observed effects on Ub structure and K63 chain formation will be enlightening to the ubiquitin and Parkin communities. Unfortunately, they were not able to demonstrate the functional significance of the minor form of pS65 Ub they found, although this will be challenging due to the equilibrium between the two states.

We thank the reviewer for their kind words and support of our study. So far, it is indeed not clear what role a new ubiquitin conformation has in context of phosphoUb. Our suspicion is that the new form can be stabilized by specific binding partners, but we have yet to identify such Ub binding protein / domain. This will be the focus of future studies.

It would have strengthened their story, if they had been able to show that pS65-K63 chains accumulated in association with damaged mitochondria in response to depolarization, using MS and their pS65-K63 AQUA standard peptide (see minor point 1 below).

We agree with this, but since we received this feedback, the published paper by the Harper lab has addressed this comprehensively. We have hence not focused on this issue.

If such pS65 K63-Ub chains do accumulate on damaged mitochondria and they are partially resistant to DUB activity, as shown here in vitro, this could serve as a means to amplify the signal for recognition by autophagy receptors (however, if this recognition involves a K63-specific Ub binding domain, this interaction would have to be unaffected by phosphate at S65 (see point 2).

Absolutely! This is exactly our line of thinking – phosphorylation of ubiquitin chains on mitochondria makes them more DUB resistant (now proven more comprehensively in **Figure 8**). Also, a perfect mitophagy adaptor should recognize phosphoUb.
We addressed whether the known K63-binding domain of TAB2, which has previously been used to label mitochondria in vivo after depolarization (van Wijk et al, Mol Cell 2012) still interacted with phosphorylated K63 chains, and it does (new **Figure 7**). Hence, this UBD does not distinguish between phosphorylated and non-phosphorylated chains.

Some consideration of the following additional points would strengthen the paper.

1. Can Pink1 also phosphorylate K48-linked Ub chains?

We have now performed in vitro phosphorylation of all tetraUb chains we have available in the lab: M1, K6, K11, K33, K48, and K63 (K11 and K63 hexaUb were in the previous version). PINK1 can access every ubiquitin in all polyubiquitin chains. This is done under native conditions, where we would expect all chains to have some kind of tertiary structure. Hence, PINK1 is a polyUb kinase (see **Figure 1**).

2. In terms of the possible functional consequences of assembly/phosphorylation of K63 Ub chains, one would like to know whether phosphorylation of K63-linked Ub chains affects the binding of K63-linkage specific ubiquitin-binding domains?

This was an important question, and we have tested this for a well-characterised K63-specific UBD, the NZF domain of TAB2 (Kulathu et al, NSMB 2009; Sato et al EMBO J 2009). We find that TAB2 binds K63 tetraUb regardless of whether it is phosphorylated or not. Hence, at least this UBD does not distinguish between Ub and phosphoUb. Also see response above.

3. Does pS65 Ub affect the linkage type of Parkin-mediated ubiquitin chains assembled in vitro? Since Ub phosphorylation by PINK1 is key to Parkin function in mitophagy, this seems like an obvious experiment to do.

A similar question was raised by Reviewer 1, and we now show that phosphoUb indeed activates Parkin when it accounts for 20% of all Ub in the reaction. However, when phosphoUb is the sole source of ubiquitin in the reaction, we see inhibition of Parkin. This was also reported by Ordureau et al.

The latter experiment rendered a clear-cut analysis of chain types less interesting. Indeed, since we submitted our manuscript, two papers have addressed which chain types Parkin makes (Durcan et al EMBO J Sept 2014; Ordureau et al, Mol Cell, 2014). We feel that we cannot add anything further to these studies at this point.

4. Can pSer65Ub adopt the minor conformation when present in chains?

This was a great question, and preliminary data suggest that this is the case. We have used K63 chains since the spectra of this chain type are identical to monoUb as there is no interface between moieties in solution. Phosphorylation of both molecules leads to the appearance of the minor species, which appears to be still in dynamic exchange with the major conformation. We have included this result as **new Supp Fig 6**. This data needs careful follow-up studies, which is beyond the scope of this manuscript.

5. Do the authors think that E3's and DUBs will in principle be able to act on pSer65 Ub when it is in the minor conformation or does it have to flip back to the major conformation?

This is an interesting question. Since the new form has not been observed before, we only ever considered the old form in Ub-ology.
Indeed, structural studies from many labs suggest that the major conformation is a prerequisite for most Ub assembly and disassembly processes. The extended Ub C-terminus is tightly bound by E2s when in complex with RING E3s, and by HECT and RBR E3s as well. Likewise, all DUBs have to bind Ub with an extended C-terminus. Hence we believe that the minor conformation is completely inert to ubiquitination/deubiquitination processes – this is not a problem in assembly and disassembly reactions since it is in dynamic equilibrium. The fact that USP DUBs still cleave a variable mix of phosphoUb chains on cIAP suggests that phosphoUb can be recognized whilst in polyUb. Clearly the characterization of the second conformation requires further studies, and its occurrence and relevance will need to be analyzed in greater detail.

6. Were any molecular dynamics simulations carried out to study the transition between the two pS65 Ub conformations? MD simulation might also indicate if unphosphorylated Ub can also adopt this altered conformation? This could be relevant to the ability of PINK1 to phosphorylate S65, since S65 is better exposed in the minor conformation for PINK1 phosphorylation.

We follow the reviewer, but would not go as far as saying that pSer65 is more exposed in the minor conformation. Our model is not based on MD simulations. However, hetNOE experiments suggest that in the minor conformation pSer65 is not part of a flexible loop, suggesting that it finds an alternative position on the ubiquitin surface. Rather than using simulations we are planning to design Ub mutants that stabilize the minor conformation to study its structure more directly.

In the major Ub conformation S65 does not appear to be readily accessible for phosphorylation by PINK1, which is relevant because ePK catalytic domains generally require the peptide backbone around the substrate hydroxyamino acid to be in an extended conformation.

This is true, but we are not sure of how canonical PINK1 is in recognizing substrates. A look at the kinome-tree is revealing in that it shows how PINK1 is at the 'root' of the tree, and has no close homologs. It is clear that PINK1 is unique and perhaps doesn't obey classical ePK recognition motifs. This will undoubtedly be the focus of future studies on PINK1.

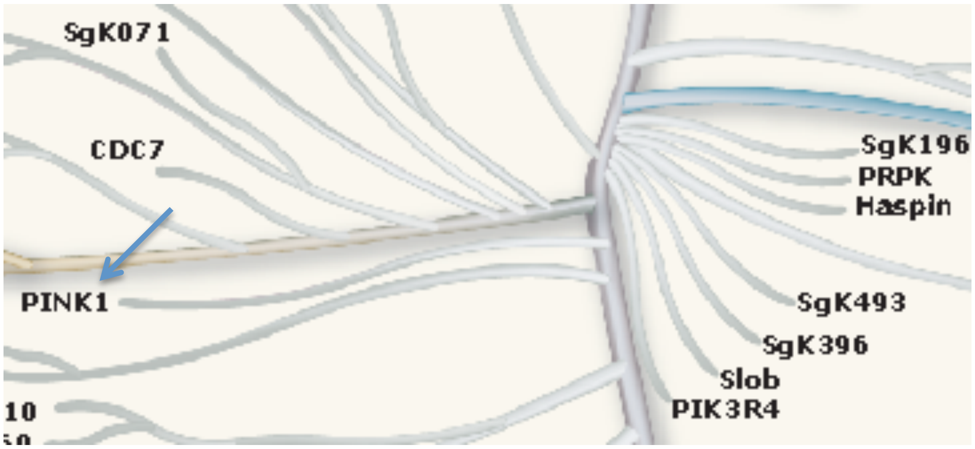

We also think that the β-strand slippage in Ub is induced only by phosphorylation. Ub is a focus of the protein folding community, the NMR community and has featured in many crystallography complexes. As far as we are aware, none of these efforts have ever encountered that the proposed β-strand-slipped conformation is present in solution in wild-type ubiquitin. There is so far no experimental evidence by NMR (i.e. NOEs or RDCs) to support wild-type Ub adopting the minor conformation. However it is now well known that ubiquitin is a highly dynamic system and might interconvert locally. A good reference point is the detailed RDC study by Griesinger and colleagues (Lange et al Science 2008 v320 p1471).

Minor points: 1. Figure 1: In this regard, the authors need to indicate exactly what pS65/K63 AQUA peptide was used and how it was made. For anything other than pS65 K63-linked Ub chains a Ser65 phosphorylated T55-K63 peptide would be the control standard, but for pS65 K63-linked Ub chains they would need a pS65-K63(GG) AQUA peptide, which is what would be obtained upon tryptic digestion. The authors need to clarify exactly what was done to quantify the extent of Ser65 phosphorylation in the experiments where they phosphorylated pre-assembled Ub chains.

We have expanded the Supplementary Material section with a more detailed description of how the AQUA mass spectrometry was performed, specifically by providing a list of AQUA peptides and fragment ions used for parallel reaction quantitation.
We have discussed our analysis with the Harper lab, who has just published similar data using the same approach for Ser65 phosphoUb quantitation. While we have independently developed this, their manuscript serves as an additional reference. For details on determining Ub chain composition, we reference Kirkpatrick et al, 2006 and Phu et al, 2010 whom initially developed this approach and provide a technically outstanding description of it. Our quantitation of chain composition is nearly identical, with the exception of instrumentation. Because of this difference we also cite Tsuchiya et al, 2013 which recently used a Q Exactive for similar studies. Taken together, this will enable researchers to easily reproduce all mass-spectrometry experiments in our paper.

2. Figure 3: How far does the K63 ε-NH2 group "move" in the pSer65 structure?

We assume that the reviewer is referring to the crystal structure?
The Lys63 side chain is fairly disordered in several of the eight molecules present in the structure, and does not adopt a single conformation. This is similar in wild-type Ub. In our structure of the major conformation, the RMSD in this region is low, <1Å for backbone atoms, but importantly, the side chain is not restricted in its movement. Hence, we are uncomfortable to put a number on a movement of a flexible side chain.

3. Figure 6: It looks as though GST-cIAP1 utilized pSer65 Ub for chain formation more efficiently than unphosphorylated Ub.

Yes, it appears that there is a minor increase, and we have seen this repeatedly. However, small changes in the levels of input monoUb could explain this. We do not think this is significant.
We also noticed that the electrophoretic running behavior of fully phosphorylated ubiquitin chains is different, even on our standard gels (4-12% gradient run in MES buffer). Phosphorylated chains seem to run at higher molecular weight (see e.g. the TAB2 experiment in **new Figure 7** as an example). This complicates the comparison of polyubiquitin ‘smears’.

4. Does pSer65 Ub compete with unphosphorylated Ub for K63 chain formation by E3 ligases such as UBE2N/UEV1A and TRAF6/UBE2D?

We have performed this experiment for UBE2N/UBE2V1, and the data is shown in **new Fig 8F**. Using Ub mutants (K63R Ub-donor, Ub/pUB ΔGG-acceptor) we monitored the assembly of K63 diUb over time, and then performed assays with increasing amounts of phosphoUb. PhosphoUb does not affect K63 diUb formation, since unphosphorylated Ub is present and can bind to UBE2V1 to serve as an acceptor.

5. With regard to the solution dynamics of the Ub fold the authors could also cite Vögeli et al. (J. Am. Chem. Soc. 131:17215).

We have included this reference.

Referee #3:

Wauer et al. present a structural and biochemical study of ubiquitin phosphorylated at Ser65 - a modification recently described as sufficient for activation of the parkin E3 ligase. In vivo, Ub is phosphorylated by the PINK1 kinase, and the authors reconstitute this activity in vitro to explore how Ub phosphorylation alters its structure via NMR and crystallography and its ability to be recognized by Ub conjugation and deconjugation machinery. While phosphoUb is recognized by the E1 enzyme, most E2 enzymes and some E3s, some E2 enzymes cannot form Ub chains using phosphoUb. Additionally, one USP DUB (USP2) is shown to have a minimal defect in processing Ub chains formed by phosphoUb. The study appears technically sound, with only a few inconsistencies (see below), and it offers an unexpected finding based on NMR that phosphoUb can adopt two conformations that are in equilibrium, a major conformation that resembles wild-type Ub and an unanticipated minor conformational wherein the last beta-strand slips into the Ub fold, effectively shortening the C-terminal tail. While these observations are of some interest, the study is largely descriptive and offers little biological insight into the role of phosphoUb or the unanticipated minor conformation. Furthermore, the biochemical data, as presented, appear somewhat preliminary, a feature of the manuscript that is somewhat offset by the mass spec analysis.

We would like to thank the reviewer for their constructive comments, which we have addressed below.

Major points:

The major/minor NMR solution structures for phosphoUb are surprising, however they are in equilibrium so it remains unclear if the minor conformation contributes to any of the associated biology/biochemistry. As no biological role is offered for the minor conformation the authors can only speculate on the existence of receptors, etc. While some E2s/E3s/DUBs can process phosphoUb, others can't, but there is no connection to whether this is due to the major/minor conformations or if the altered electrostatic surface properties of phosphoUb are responsible. As the latter case is deemed more likely the authors should augment the biochemical studies using a phosphomimetic of phosphoUb to determine if it recapitulates E1/E2/E3/DUB activities with phosphoUb.

We fully agree with all points in this assessment. We discuss this in more detail in our response to Reviewer 2.

As suggested, we have now investigated the Ub S65E mutant.
Interestingly, while this mutant is similar to the major conformation, there are no signs of a minor conformation in NMR. This is important as it suggests that a Glu at position 65 does not fully recapitulate the structural perturbations of Ub induced by phosphorylation. Phosphomimetic mutants have been used in the recent literature, and seem to have an effect on Parkin biology, although these were all overexpression studies.

Furthermore, despite not showing a minor conformation, the S65E mutant is inactive with UBE2N/UBE2V1 and significantly less active with TRAF6/UBE2D (**new Supp Fig 13**). The fact that the mutant recapitulates the effects of phosphoUb despite having only the major conformation reveals that the observed effects can be attributed to the change in surface charge, rather than an involvement of the minor Ub conformation.

Given the available toolkit of DUBs in the Komander lab, a more thorough analysis of DUBs might be expected.

This is a fair point which we have addressed.
We now show additional data for

USP8 (Parkin DUB)
USP15 (mitophagy DUB)
USP30 (mitophagy DUB)
Ataxin-3 (MJD, also linked to Parkin)
AMSH (JAMM)
OTUB1, TRABID and OTULIN (OTUs).
In assays similar to the ones before as well as in new assays against more defined substrates (tetraUb of defined linkage types). We find that USP21 and vOTU remain the only DUBs that does not show significant difference towards phospho-polyUb, while all other DUBs are significantly less active against phosphorylated Ub substrates.
Together this gives a more comprehensive picture of how DUBs process phosphoUb vs Ub chains, and hopefully satisfies the reviewer.

Additionally, USP2 (which the authors claim shows reduced cleavage of phosphUb) goes form barely any cleavage (Figure 7B) to respectable cleavage (Suppl Fig 10G) by a two-fold increase in enzyme concentration - a result that warrants further investigation/comments. Suppl Fig 10G is not included in the legend and is not referred to in the text. Perhaps it is a typo/mis-label of the panels?

Indeed, it was an oversight that we had included the USP2 panel in Figure 10G,

as this suffered from an experimental problem. It is very clear from that gel that the reaction had been started wrongly, as even at the zero time point, most of the polyUb is already gone. Hence, the time course is not correct, and the data is not sound. This explains the apparent disconnect between (old) Fig 7B (now 8B) and old Supp Fig 10G (now removed). However the result at lower concentration has been reproduced multiple times and we show this gel in Figure 8B.
We clearly state in the text that USP2 does cleave phosphoUb chains at later time points or at higher enzyme concentrations. We believe that this, together with the data we show for 4 other USPs, should provide the correct picture.

Minor comments:

Figure 1 - Why has analysis of K48 chains (a relevant chain type for parkin function) not been included? panel Figure 1B, the figure should indicate that it is a phostag gel (not only the legend).

We have now done assays on tetraUb of M1, K6, K11, K33, K48 and K63 chains, representing all chain types available to us. See comment to Reviewer 1 above. We have also clearly labelled the phostag gel in Figure 1B.

When discussing Figure 3 (page 7), there is no mention of chains G/H in the crystal structure (not utilizing the Ile44 patch). What crystal contacts are they making?

These chains are 'lifted off' the remaining Ub molecules and are breaking the symmetry of the structure. While they are involved in crystal packing, they are really not making any significant contacts that have been observed before (such as Ile44 or Ile36 contacts). We are more than happy to send the coordinates so the reviewer can evaluate this independently.

Figure 5, panel A - what does the AQUA profile reveal for Cdc34?

We thank the reviewer for this comment. As for some of the other chain assembling E2s, we had repeatedly observed a small defect for cdc34 in making diUb in vitro. Repeating the assay with a different batch of enzyme has exaggerated this effect (new panel shown in Fig 5A). Charging of cdc34 is identical, however discharging to Ub to form free K48 chains is defective. As suggested, we have also quantified this by AQUA – despite being much less active, cdc34 still makes mostly K48 linkages with phosphoUb (new panels in **Figure 5**).

Figure 6, the use of bar graphs and pie charts appears redundant in panels 6C/D and 6G/H

We don't quite agree on this. We need to show that the linkage composition is similar with Ub and phosphoUb, which is also the case for TRAF6 (although

there is a trend that Lys63 linkages are lost predominantly. As mentioned above, we have toned down our description of TRAF6. This manuscript focuses on phosphoUb.

Figure 6, what is the effect of phosphoUb on parkin activity in vitro? This might also provide insight into phosphoUb on K48 chain formation.

We have performed this assay and included this at the start of the manuscript. We found that in assays with only phosphoUb, Parkin remains inactive. This is interesting, although we do not yet have an explanation for this. Please see response to Reviewer 1.

Supplementary Figure 10F and 10G - the inputs for the DUB reaction between Ub and phosphoUb are not the same, making comparisons difficult. The position of the bands for different DUBs should be labeled on the gels.

As mentioned above, the reason why the inputs are different is that for both enzymes at this concentration the zero time point already shows significant DUB activity. We have hence removed previous Supp Fig 10G on USP2.
We now provide many additional assays for many different DUBs, and used freshly assembled substrates which appear more similar at time zero. Please also see comments to reviewer 2 above (re cIAP being apparently more active with pUb).
Moreover, We always refrain to derive quantitative statements from our silver stained gels, as the staining is not linear. We only consider this as qualitative comparison. When this is done, it is always clear that pUb is cleaved with less activity by USP2, and similarly by USP21. Other USP DUBs and also Atx3 have a more equal distribution.

Supplementary Figure 10G - the legend is missing or mislabeled.

We apologise for the lack of description for Supp Fig 10G, which was shown inadvertently – hence the lack of Figure legend. Please see above.

Discussion section (page 18, end of top paragraph). It may be relevant to discuss the activities of existing DUB-resistant Ub mutants in the context of phosphoUb.

Does the reviewer refer to the L73P mutant reported by the Salvesen / Huang labs? This is indeed a very good idea, and in fact this may stabilize the minor conformation. This is exactly the direction we want to take this project towards, with the key aim to stabilize the minor conformation. However, rather than discussing this at length here, we would prefer to discuss this in a future manuscript.

Accept letter 21 November 2014

Thank you for submitting your revised manuscript for our consideration. It has now been seen once more by two of the original referees (see comments below), and I am happy to inform you that they are both satisfied with the revisions and therefore have no further objections towards publication in The EMBO Journal.

Referee #1:

The revised manuscript is significantly improved particularly in the demonstration of inhibition of DUB activity as a novel feedforward mechanism for the induction of mitophagy.

Referee #2:

The paper has been strengthened by a large amount of new data to address the reviewers' comments, and all of my points have been satisfactorily addressed. In particular, the paper has been strengthened by the new Parkin assay data in Figure 1, which shows interestingly that pS65 Ub appears not to be a substrate for Parkin E3 ligase activity, and by the more extensive survey of the ability of different DUBs to act on pUb chains, showing that most DUBs are adversely affected by a phosphate at S65, consistent with the idea that PINK1 phosphorylation of Ub chains associated with damaged mitochondria will stabilize these chains to amplify the signal. What is left unanswered is whether there is a specific pS65 Ub chain binding domain that would selectively recognize phosphorylated Ub chains, perhaps as a consequence of the minor pUb conformation, on a damaged mitochondrion and thereby promote its elimination by mitophagy. There is a huge amount of useful data in this paper, which make an important contribution to this very fast moving field, and I recommend the paper be accepted as is.

© European Molecular Biology Organization
